# Noncanoncial signal recognition particle RNAs in a major eukaryotic phylum revealed by purification of SRP from the human pathogen *Cryptococcus neoformans*

**DOI:** 10.1093/nar/gkv819

**Published:** 2015-10-10

**Authors:** Phillip A. Dumesic, Magnus A. Rosenblad, Tore Samuelsson, Tiffany Nguyen, James J. Moresco, John R. Yates, Hiten D. Madhani

**Affiliations:** 1 Department of Biochemistry and Biophysics, University of California, San Francisco, CA 94158, USA; 2 Department of Chemistry and Molecular Biology, University of Gothenburg, Gothenburg, SE-405 30, Sweden; 3 Department of Medical Biochemistry and Cell Biology, Institute of Biomedicine, The Sahlgrenska Academy, University of Gothenburg, Gothenburg, SE-405 30, Sweden; 4 Department of Chemical Physiology, The Scripps Research Institute, La Jolla, CA 92037, USA

## Abstract

Despite conservation of the signal recognition particle (SRP) from bacteria to man, computational approaches have failed to identify SRP components from genomes of many lower eukaryotes, raising the possibility that they have been lost or altered in those lineages. We report purification and analysis of SRP in the human pathogen *Cryptococcus neoformans*, providing the first description of SRP in basidiomycetous yeast. The *C. neoformans* SRP RNA displays a predicted structure in which the universally conserved helix 8 contains an unprecedented stem-loop insertion. Guided by this sequence, we computationally identified 152 SRP RNAs throughout the phylum Basidiomycota. This analysis revealed additional helix 8 alterations including single and double stem-loop insertions as well as loop diminutions affecting RNA structural elements that are otherwise conserved from bacteria to man. Strikingly, these SRP RNA features in Basidiomycota are accompanied by phylum-specific alterations in the RNA-binding domain of Srp54, the SRP protein subunit that directly interacts with helix 8. Our findings reveal unexpected fungal SRP diversity and suggest coevolution of the two most conserved SRP features—SRP RNA helix 8 and Srp54—in basidiomycetes. Because members of this phylum include important human and plant pathogens, these noncanonical features provide new targets for antifungal compound development.

## INTRODUCTION

The signal recognition particle (SRP) is a ribonucleoprotein that directs protein sorting in all three domains of life (1), targeting substrates to the ER membrane in eukaryotes and to the plasma membrane in bacteria and archaea. The mammalian SRP comprises a single RNA molecule and six proteins: SRP9, SRP14, SRP19, SRP54, SRP68 and SRP72 (2). The RNA is largely double-stranded, and folds into a Y shape whose halves carry out distinct functions. The base of the Y constitutes the Alu domain: it binds to the SRP9/14 heterodimer and is responsible for translational arrest of SRP-bound ribosomes (3). The forked half of the RNA constitutes the S domain. This region binds the remaining SRP proteins and functions in signal sequence recognition and docking with the SRP receptor (3). The SRP binds translating ribosomes such that the Alu domain is near the ribosome's elongation factor binding site and the S domain is near the nascent peptide exit site (4).

A typical archaeal genome encodes an SRP RNA—with a secondary structure similar to that of mammalian SRP RNA—as well as two SRP protein components: SRP19 and SRP54. In contrast, most bacterial genomes, including that of *E. coli*, encode an even more simplified SRP consisting of a minimal SRP RNA (4.5S RNA) and a single protein: Ffh, a homolog of SRP54 (1). This SRP represents the universal core of SRP, since SRPs in all domains of life contain an SRP54 homolog and the hairpin region of the SRP RNA to which it binds. SRP54 binds to signal peptides displayed at the ribosome via its C-terminal M (methionine-rich) domain (5). It also mediates GTP-dependent complex formation with the SRP receptor via its central G (GTPase) domain (6). These functions of SRP54 are regulated by a hairpin stem-loop of the SRP RNA called helix 8, which binds to the SRP54 M domain and is conserved in all SRP RNAs (1,7). It promotes the interaction between SRP54 and the SRP receptor and provides a scaffold for the protein rearrangements that initiate translocation (8).

Despite the remarkable conservation of SRP, there is a paucity of identified SRP components in the lower eukaryotes, particularly fungi (1,9). For example, in the phylum Basidiomycota, which includes numerous pathogenic fungi, SRP RNAs have not been described in any species despite the availability of over 100 genome sequences. Here we describe the purification and analysis of SRP from the basidiomycetous yeast *Cryptococcus neoformans*. It includes the six canonical SRP proteins as well as a single SRP RNA. The RNA is predicted to form an unusual secondary structure in which the Alu domain resembles that of ascomycetous fungi, whereas the S domain contains a dramatic stem-loop insertion within the universally conserved helix 8. The unusual structural features of this SRP RNA explain why it was not previously identified by computational approaches, and its sequence enabled us to identify SRP RNA genes in other basidiomycetes, revealing a series of unprecedented helix 8 alterations. Intriguingly, these features impact a region of helix 8 known to interact with the SRP subunit Srp54. Correspondingly, we find that Srp54 in basidiomycetes contains unusual amino acid sequence alterations within its highly conserved RNA-binding domain. These findings expand our understanding of the SRP components in fungi and provide evidence in this lineage for coevolution of the most conserved SRP components.

## MATERIALS AND METHODS

### Yeast strains and techniques

*C. neoformans* strains used in this study were derived from strain H99 and established by standard procedures (Supplementary Table S1) (10). Genes were identified using Broad Institute (Cambridge, MA) annotations of the *var. grubii* H99 genome, where genes are named ‘CNAG_#.’ Strains were grown in YPAD medium (1% yeast extract, 2% Bacto-peptone, 2% glucose, 0.015% L-tryptophan, 0.004% adenine).

### Tandem affinity protein purification

*C. neoformans* strains encoding CBP-2xFLAG-tagged proteins expressed from their endogenous promoters were grown in YPAD media, harvested, snap frozen and lysed using a coffee grinder. Proteins of interest were purified using anti-FLAG M2 resin (Sigma) and calmodulin resin (Stratagene), then analyzed by mass spectrometry as described in Supplementary Table S2 and elsewhere (11).

### RNA immunoprecipitation and detection of protein-associated transcripts

Epitope-tagged proteins were purified using anti-FLAG resin as described above, then eluted with 3xFLAG peptide (Sigma). Protein-associated RNA was isolated by phenol-chloroform extraction as described previously (11), then treated with DNaseI (DNA-free, Ambion). To detect all RNA species, the protein-associated RNA was dephosphorylated using Calf Intestine Alkaline Phosphatase, then 5′ end radiolabeled using T4 polynucleotide kinase (KinaseMax, Ambion). Labeled RNA was resolved alongside a single-stranded RNA ladder (New England Biolabs) in a 6% polyacrylamide gel with 7 M urea. To quantitatively detect specific transcripts, cDNA was generated from protein-associated RNA according to manufacturer's instructions using SuperScript III reverse transcriptase (Invitrogen) with oligo-dT_20_N (38 ng/μl) and random 9-mers (10 ng/μl) as primers. The levels of particular transcripts were determined by qPCR and normalized to transcript abundance in the input fraction, as well as to transcript abundance in an immunoprecipitation from a wild-type strain lacking an epitope-tagged protein. Primers used for PCR are listed in Supplementary Table S3.

### RNA isolation and northern blot

Total RNA was isolated using TRIzol (Invitrogen) and resolved alongside a single-stranded RNA ladder (New England Biolabs) in a 6% polyacrylamide gel containing 7 M urea. The RNA was transferred to a Hybond-XL nylon membrane (Amersham) and crosslinked by UV irradiation (120 mJ; UV Stratalinker 2400, Stratagene). Crosslinked membranes were blocked with ULTRAhyb-Oligo solution (Ambion) for 30 min at 42°C, then incubated overnight at 47°C with DNA probes complementary to the SRP RNA (Supplementary Table S3), which had been 5′ end radiolabeled according to manufacturer's instructions (KinaseMax, Ambion). Hybridized membranes were washed four times, 30 min each (2x SSC, 0.5% SDS), then imaged using a storage phosphor screen (Amersham).

### RNA cloning

To clone the SRP RNA, SRP-associated RNA (450 ng) was purified as described above and ligated to a 3′ linker (400 pmol Linker-1 oligo, Integrated DNA Technologies) using T4 RNA ligase I (20 U, New England Biolabs) (12). The reaction was purified over an RNeasy spin column (Qiagen) and used as a template for SuperScript II reverse transcriptase (Invitrogen). cDNA synthesis was primed by an oligonucleotide complementary to the 3′ linker (275 nM) and terminated with the addition of a 5′ linker via reverse transcriptase template switch to an oligonucleotide substrate (275 nM) (13). The resulting cDNA was amplified by PCR using primers complementary to the linker sequences, which yielded a single product of approximately 300 nt. The product was subsequently gel purified and cloned into the TOPO 2.1 vector (Invitrogen) according to manufacturer's instructions. Six clones were sequenced, which all corresponded to the same RNA sequence—the presumptive SRP RNA—and terminated in the same poly-U tract, consistent with synthesis by RNA Pol III. The SRP RNA 5′ end was verified by 5′ RACE, as described below. All cloning primers are listed in Supplementary Table S3.

### 5′ RACE

To validate the SRP RNA 5′ end, cDNA was generated from 250 ng SRP-associated RNA using SuperScript III reverse transcriptase (Invitrogen) primed by an oligonucleotide complementary to the SRP RNA. The cDNA was purified using a Microcon-50kDa centrifugal filter unit (Millipore) and poly-A tailed with terminal transferase (New England Biolabs). The tailed product was amplified by PCR using standard methods (14), gel purified, and cloned into the TOPO 2.1 vector (Invitrogen) according to the manufacturer's instructions. Five clones were sequenced, which all corresponded to the same SRP RNA transcription initiation site. Primers used for cloning are listed in Supplementary Table S3.

### Acquisition and alignment of SRP protein sequences

Orthologs of SRP9/21, SRP54 and SRP72 in Basidiomycota were obtained using two different methods. First, they were identified in BLAST searches against the NCBI protein database. Second, they were predicted with GeneWise (Wise2) (15), where genomic sequences were searched with hmmer models (http://hmmer.janelia.org) based on sequences obtained by the first method. Ascomycete sequences were obtained from the SRP database (16), and chloroplast sequences were obtained from (17). Protein sequences were aligned using Clustal Omega (18). Prediction of SRP54 ortholog residues corresponding to M domain alpha helices was based on (19,20). The structural location of inserted residues in *C. neoformans* Srp54 was predicted using Phyre 2 (21) modeling based on a structure of its ortholog in *M. jannaschii* (PBD ID: 2V3C) (22).

### Identification of SRP RNA sequences

Prediction of SRP RNA was carried out using the Infernal software (23). An alignment was first created using cmalign based on the SRP RNA model for fungi in the Rfam database (RF01502) (24) as well as a selection of eukaryotic SRP RNA sequences including that of *C. neoformans*. This alignment was used to create a covariance model using cmbuild. A search using cmsearch among basidiomycete genomes then identified a range of novel SRP RNA homologs. An iterative procedure was then used in which all new sequences identified in a search were added to create a new model with the aid of cmalign and cmbuild. This procedure was repeated until no more novel sequences could be identified. RNA secondary structures were predicted by UNAfold (25) and described using the nomenclature of (7). Phylogenetic trees for comparison of SRP RNA sequences were adapted from (26).

## RESULTS AND DISCUSSION

### Purification of the *C. neoformans* SRP and identification of its RNA subunit

Although the SRP RNA is universally conserved, computational approaches have failed to identify this noncoding RNA in the yeast phylum Basidiomycota (9). Thus, the SRP RNA may exhibit unexpected diversity in the fungal kingdom, diverging from its canonical structure. We therefore sought to experimentally identify the SRP RNA in *C. neoformans*, a basidiomycetous yeast and human fungal pathogen.

**Figure 1.**
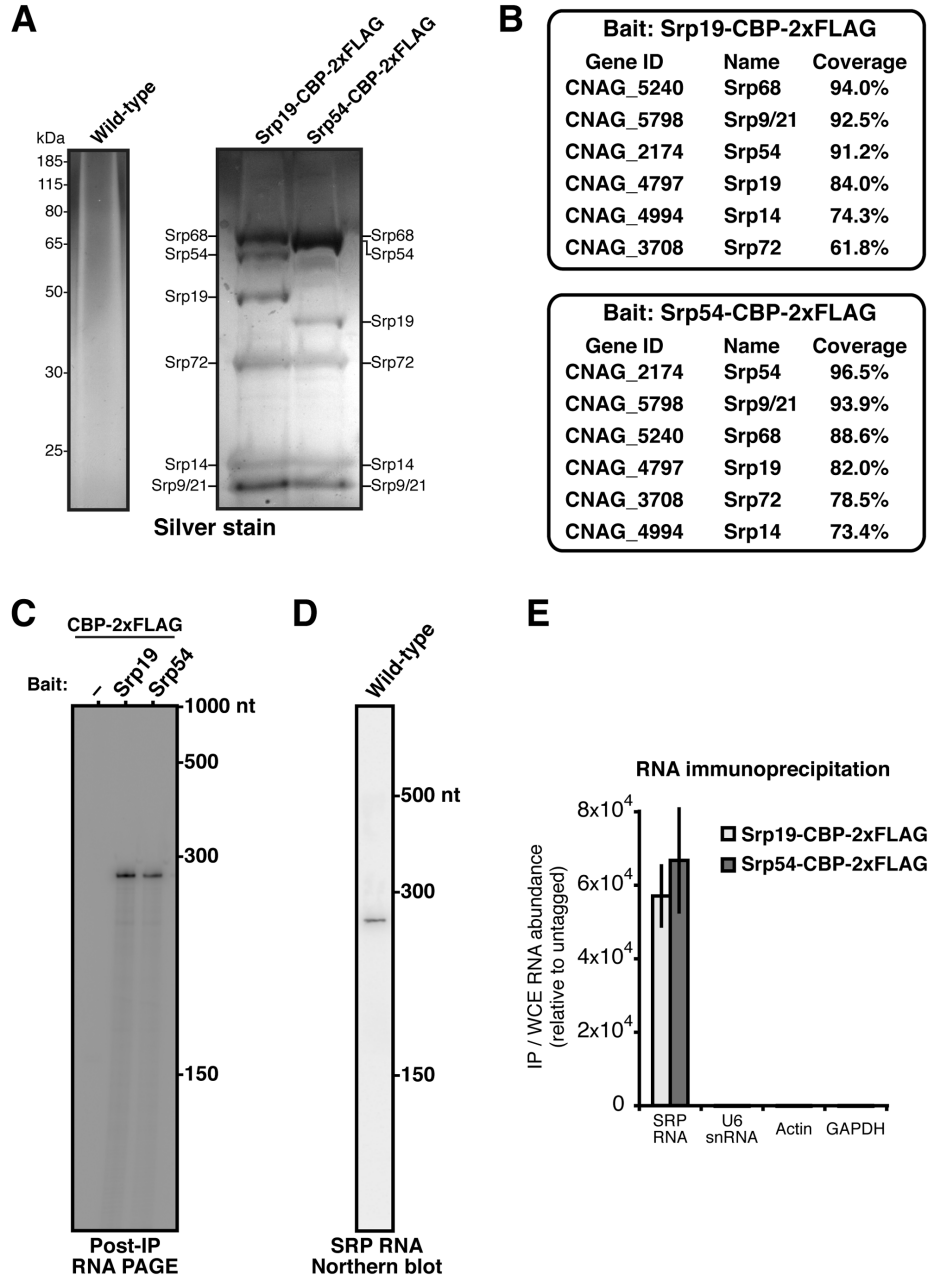
Biochemical purification of the *C. neoformans* SRP and cloning of its RNA subunit. (**A**) Silver stain of protein isolated upon tandem affinity purification from *C. neoformans* strains that expressed epitope-tagged SRP subunits. As a control, an equivalent purification was performed from wild-type, untagged cells. (**B**) Proteins associated with Srp19 or Srp54 by tandem affinity purification, as identified by mass spectrometry. Percent sequence coverage is calculated as the average of two replicate purifications. Likely contaminants and proteins with <10% sequence coverage have been excluded. (**C**) RNA associated with Srp19 or Srp54 by tandem affinity purification. After each indicated protein purification, associated RNA was isolated, end labeled with ^32^P, and resolved by denaturing PAGE. The single detected RNA species—the putative SRP RNA—was cloned to determine its sequence (see text). Size markers correspond to a ssRNA ladder. (**D**) Northern blot to detect SRP RNA in total cellular RNA from *C. neoformans*. A radiolabeled probe was designed to target the RNA species cloned in (**C**). Size markers correspond to a ssRNA ladder resolved by denaturing PAGE. (**E**) Interaction of Srp19 and Srp54 with the SRP RNA in cells. Levels of individual RNAs co-immunoprecipitated with Srp19 or Srp54 were assessed by RT-qPCR and normalized to their abundance in whole-cell extract. Transcript level is relative to that obtained in purifications from wild-type (untagged) lysates. Error bars represent SD.

Examination of the *C. neoformans* genome revealed orthologs of all six SRP protein components. We constructed strains in which the endogenous Srp19 or Srp54 was tagged with a CBP-2xFLAG epitope. When either tagged protein was isolated by tandem affinity purification, it co-precipitated with five additional proteins, which were not obtained from an untagged strain (Figure 1A). Mass spectrometry identified these proteins as the remaining five SRP protein subunits (Figure 1B). The annotated open reading frames of all SRP protein components (*C. neoformans var. grubii* H99 sequencing project, Broad Institute) were confirmed by cDNA sequencing.

To identify RNA associated with the *C. neoformans* SRP, we isolated nucleic acid from purified SRP complexes, treated it with DNase, and radioactively end-labeled the remaining nucleic acid. When resolved by denaturing PAGE, one RNA species of approximately 300 nt was detected (Figure 1C). Equivalent purifications from untagged strains did not yield this RNA. To determine the identity of this putative SRP RNA, we ligated it to a 3′ linker, which was then used as a primer-binding site for first strand cDNA synthesis. A 5′ linker was subsequently added by reverse transcriptase template switch, which enabled PCR amplification, cloning and sequencing of the cDNA (13). The resulting sequence was used to design probes for Northern hybridization, which demonstrated that the ∼300 nt SRP RNA observed in SRP purifications is present at the same size in total cellular RNA (Figure 1D). To confirm the identity of this RNA, we examined SRP subunits by RNA immunoprecipitation. In this assay, Srp19 or Srp54 was purified from cells and its associated RNA was examined by RT-qPCR. Each protein associated with the SRP RNA, but not with other high-abundance nuclear or cytoplasmic transcripts (Figure 1E).

Sanger sequencing demonstrated that the cloned RNA is 280 nt and possesses features of canonical SRP RNAs. First, its 5′ end, which was independently confirmed by 5′ RACE, begins with a purine, as is usually the case for eukaryotic SRP RNAs (1). Its 3′ end is a poly-U tract, the typical termination sequence for SRP RNAs, which are synthesized by RNA Pol III. Furthermore, the RNA maps to a region of the genome distinct from annotated coding regions, without evidence of introns. Multiple attempts to knock out this genomic locus were unsuccessful, suggesting that it is essential for viability.

### The *C. neoformans* SRP RNA exhibits unprecedented predicted structural features

**Figure 2.**
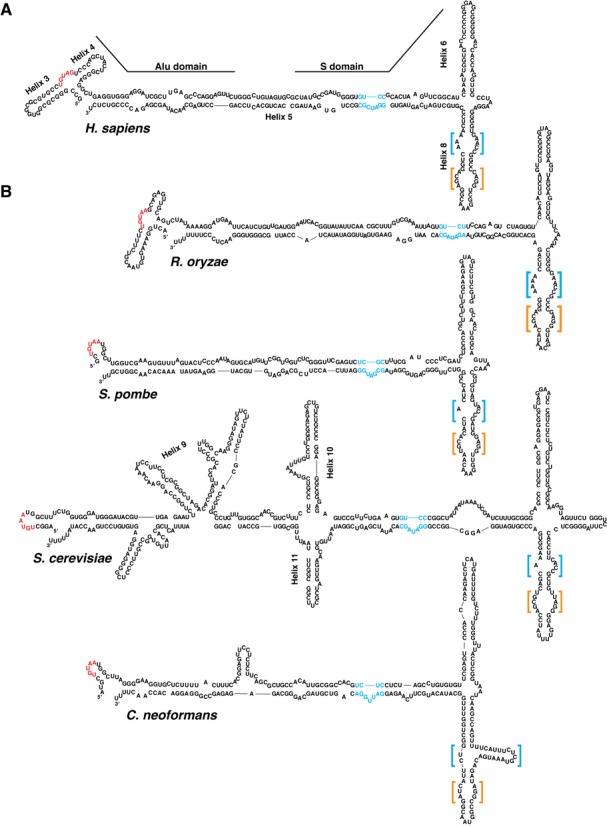
Predicted secondary structures of SRP RNAs in mammals and yeast. (**A**) The secondary structure of the human SRP RNA. In the Alu domain, the UGUNR motif is colored in red. Helices 3 and 4, which bind to SRP9/14, are labeled. In the S domain, blue text indicates the conserved 5e motif, which binds SRP72. Helix 6, which binds SRP19, and helix 8, which binds SRP54, are labeled. Orange and blue brackets indicate the symmetric and asymmetric loops, respectively, of helix 8. (**B**) The secondary structure of fungal SRP RNAs. Included species are the ascomycetes *S. pombe* and *S. cerevisiae*, the zygomycete *R. oryzae*, and the basidiomycete *C. neoformans*. Conserved structural features are indicated as in (**A**).

We compared the secondary structure of the *C. neoformans* SRP RNA to that of other eukaryotic SRP RNAs. The canonical eukaryotic SRP RNA is that of mammals; its two halves correspond to the Alu and S domains (Figure 2A). The Alu domain encodes two helices (helices 3 and 4) whose loops take part in a tertiary interaction. Between these two helices resides the sequence motif UGUNR (where N is any base and R is a purine), which may be important in establishing the folded shape of the Alu domain for binding to the SRP9/14 heterodimer. Conserved motifs in the S domain include helix 5e—a 3 nt asymmetric loop that mediates binding to SRP72—and helices 6 and 8, which bind to SRP19 and SRP54, respectively (1).

The predicted fold of the *C. neoformans* SRP RNA Alu domain, unlike that of mammals, lacks helices 3 and 4, and in this regard resembles Alu domains of ascomycetous yeast such as *S. pombe* and *S. cerevisiae* (Figure 2B). In contrast, the basal fungus *R. oryzae*, which resides in the phylum Zygomycota, encodes a metazoan-like Alu domain (27). Despite these differences, the fungal Alu domains universally contain UGUNR motifs, which tend to reside in loop regions, as in metazoan SRP RNAs (Figure 2A and B).

Near the boundary between the Alu and S domains, the *C. neoformans* SRP RNA exhibits a small helix 10, but no evidence of helix 11 (Figure 2B). These two helices, which are widespread in fungal SRP RNAs, are individually dispensable in *S. cerevisiae* but exhibit genetic interactions with other, more conserved segments of the SRP RNA (28). As expected, the *C. neoformans* SRP RNA lacks helix 9, which is an insertion specific to the *Saccharomyces* lineage (27,29) (Figure 2B).

Whereas the Alu domain contains several fungal-specific elaborations, the S domain is more highly conserved across eukaryotes. Indeed, the *C. neoformans* SRP RNA shows evidence for the three most conserved helices in this domain: 5, 6 and 8 (Figure 2A and B). Helix 5 connects the Alu and S domains and is the longest SRP RNA helix. Within it, a small asymmetric loop—the 5e motif—introduces a kink to the helix and mediates a physical interaction with SRP72 (30,31). Although the structure of this loop is diverse in the fungal lineage, it appears with a canonical 3 nt size in *C. neoformans*. Helices 6 and 8, the conserved binding sites of SRP19 and SRP54, respectively, are also observed in the *C. neoformans* SRP RNA. The apical loops of these helices contain conserved adenosines—at the third loop position in helix 6 and the fourth loop position in helix 8—that mediate a tertiary interaction conserved in all eukaryotic and archaeal SRP RNAs (7).

**Figure 3.**
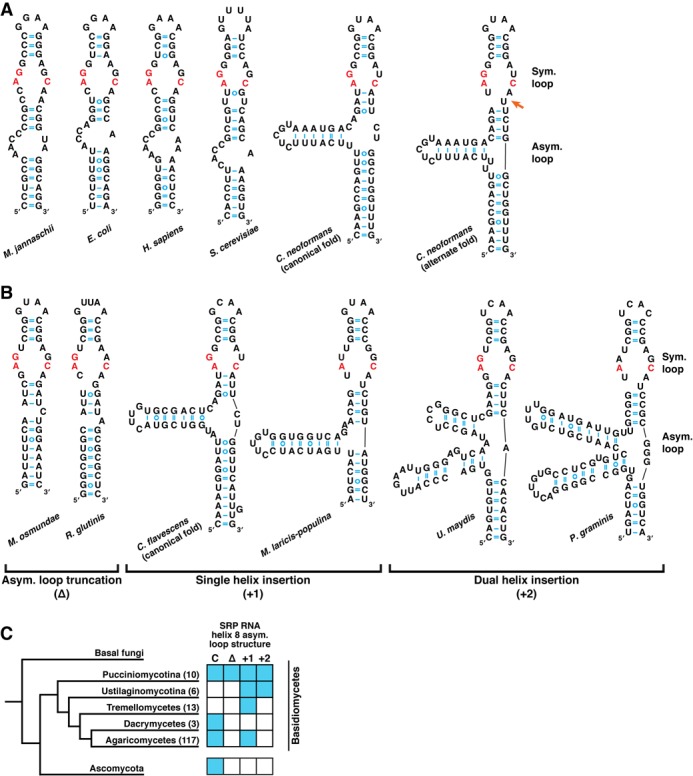
Novel features alter helix 8 of the SRP RNA in basidiomycetous yeast. (**A**) SRP RNA helix 8 secondary structures from archaea (*M. jannaschii*), bacteria (*E. coli*), metazoa (*H. sapiens*), ascomycetous yeast (*S. cerevisiae*), and basidiomycetous yeast (*C. neoformans*). The symmetric loop contains two universally conserved noncanonical base pairs: an A-C pair and an adjacent G-G/A pair, both of which interact with SRP54. The invariant bases are colored in red. The asymmetric loop interacts with SRP54 and mediates a tertiary interaction with SRP RNA helix 6. Two potential folds of the symmetric loop are shown for *C. neoformans*, one of which breaks the loop's symmetry (orange arrow). The latter fold is supported by secondary structure-based alignments of the newly identified SRP RNAs (see text for details). (**B**) Types of novel asymmetric loop alterations observed in basidiomycete SRP RNAs. Truncation alterations result in an asymmetric loop whose 5′ strand, instead of the 3′ strand, is the shorter of the two strands. Insertions of either one or two separate stem-loops into the asymmetric loop 5′ strand are also observed. (**C**) Distribution of the helix 8 asymmetric loop alterations throughout Basidiomycota. SRP RNAs were identified within the subphyla Pucciniomycotina and Ustilaginomycotina, and the classes Tremellomycetes, Dacrymycetes and Agaricomycetes, with the number of RNAs in each listed in parentheses. The grid indicates the presence of species encoding each class of asymmetric loop structure: canonical structure (**C**), 5′ strand truncation (Δ), single helix insertion (+1) and dual helix insertion (+2). The phylogenetic tree was adapted from (26).

The most conserved region of the S domain is helix 8, which is present in all domains of life. It contains two internal loops: a symmetric loop near the apex of the helix and an asymmetric loop near the base of the helix (Figure 3A). The symmetric loop consists of contiguous noncanonical base pairs, including an A-C pair that is invariant in known SRP RNAs (7). Adjacent to this pair, on the 5′ strand of the loop, is an invariant guanosine that also participates in noncanonical base-pairing interactions (Figure 3A). These neighboring pairs form a surface for binding SRP54 (19). In addition, SRP54 interacts with the asymmetric loop of helix 8. In this loop, protein interaction induces a change in RNA structure: the bases in the loop's long strand stack against each other and interact with SRP54, whereas the bases in the short strand turn out away from the helix and participate in a tertiary interaction with helix 6 (7,32).

The predicted secondary structure of the *C. neoformans* helix 8 exhibits alterations from the canonical structure. Typically, the asymmetric loop's 5′ strand is 2–3 nt longer than its 3′ strand. In *C. neoformans*, however, the 5′ strand contains an insertion predicted to form a 17 nt stem-loop (Figure 3A). This unusual feature raises the possibility that the asymmetric loop is improperly assigned in this predicted structure. Arguing against this, however, is the asymmetric loop's proximity to a putative symmetric loop that contains several expected features, such as an A-C base pair and contiguous guanosine (Figure 3A). Furthermore, the fact that the loop sequence was present in SRP RNA cloned from mature, purified SRP complexes argues against the idea that the loop insertion represents an intronic sequence removed during SRP RNA maturation.

The extensive alterations of its helix 8 secondary structure, as well as its low sequence similarity compared to ascomycete SRP RNAs, explain why the *Cryptococcus* SRP RNA had not been previously identified by computational approaches, since these approaches are focused on the canonical helix 8 secondary structure (9).

### Identification of SRP RNAs throughout Basidiomycota reveals a range of helix 8 structures

We utilized the *Cryptococcus* SRP RNA sequence to modify our computational model of SRP RNA, which we subsequently used to search other basidiomycete genomes. We took an iterative approach so that newly identified SRP RNA homologs would be included in the model. This strategy yielded 152 putative RNAs (Supplementary Dataset S1). These include SRP RNAs in the three subphyla of Basidiomycota: Pucciniomycotina, Ustilaginomycotina and Agaricomycotina.

In order to assess conserved structural features of the SRP RNAs in Basidiomycota, secondary structure predictions were generated using UNAfold (25). Like the SRP RNA of *C. neoformans*, the basidiomycete SRP RNAs broadly lack the Alu domain helices 3 and 4. They also lack the *Saccharyomyces*-specific helix 9. Their Alu domains thus appear equivalent to that of ascomycete SRP RNAs outside the *Saccharyomyces* lineage. Consistent with this similarity, both ascomycetes and basidiomycetes exhibit alterations in the SRP protein components that bind to this region: they lack Srp9 and instead encode the structurally-related but larger protein Srp21 (27).

In the S domain, the basidiomycete SRP RNAs, like other yeast SRP RNAs, contain the 5e motif. However, in contrast to the ascomycetes, which encode SRP72 orthologs of the standard size, the basidiomycetes, including *C. neoformans*, encode SRP72 orthologs that are drastically reduced in size. These proteins nevertheless retain the C-terminal RNA-binding domain thought to interact with the 5e motif, consistent with the idea that this domain is important for SRP72 assembly into the SRP (Supplementary Figure S1) (30).

Whereas their overall architecture is similar, the basidiomycete SRP RNAs encode a striking variety of helix 8 structures, ranging from canonical folds to folds with alterations in both the asymmetric and symmetric loops. As described above, the *C. neoformans* SRP RNA is predicted to contain a helical insertion within the 5′ strand of the helix 8 asymmetric loop (Figure 3A). Similar insertions were found in closely related yeast: all tremellomycete SRP RNAs contained an inserted helix (Supplementary Table S4). In several such organisms, such as *Cryptococcus flavescens*, the insertion sequence varies and yet its base pairing is retained, arguing for the evolutionary conservation, and potential functional importance, of its unusual secondary structure (Figure 3B). More distantly related basidiomycetes encode a variety of predicted asymmetric loop structures. For instance, *U. maydis*, of Ustilaginomycotina, and *P. graminis*, of Pucciniomycotina, contain larger insertions in the 5′ strand that are predicted to encode two helices (Figure 3B). In contrast, the Pucciniomycotina members *M. osmundae* and *R. glutinis* are predicted to encode a truncation of the asymmetric loop 5′ strand such that this strand, which is typically the longer strand, is instead shorter than its partner 3′ strand (Figure 3B). Still other species, especially among the Agaricomycetes, encode canonical asymmetric loop structures that do not contain an insertion. Arguing against the idea that the helix 8 asymmetric loop insertions in Basidiomycota represent intronic sequences, the insertions are present in SRP RNA sequences found in *U. maydis* and *M. violaceum* EST databases, with no evidence of a spliced alterative form.

Unusual alterations among the basidiomycete SRP RNAs were observed not only in the helix 8 asymmetric loop, but also in the symmetric loop, which forms a flat minor RNA groove that interacts directly with SRP54 (19). All newly identified SRP RNAs contain the invariant noncanonical A-C base pair. However, the neighboring invariant guanosine is altered to a uridine in *M. laricis-populina, P. striiformis* and *D. cryoxerica*, and to an adenosine in *P. graminis* (Figure 3B).

Secondary structure-based alignments of the newly identified SRP RNAs provide additional evidence for significant alterations of the symmetric loop's structure in basidiomycetes. These alignments predict that multiple SRP RNAs, particularly among Pucciniomycotina and the tremellomycetes, contain a 1 nt insertion within the symmetric loop 3′ strand, thereby making it asymmetric (Supplementary Table S4). In one such species, *C. neoformans*, there are two alternative fold structures of similar stability—a ‘canonical fold’ in which the symmetric loop remains symmetric and an ‘alternate fold’ in which this loop's symmetry is broken (Figure 3A). In other such species, such as *P. graminis* and *R. glutinis*, the ‘alternate fold’ represents the single most preferred structure, thereby providing stronger evidence for a significant alteration of symmetric loop folding in these species (Figure 3B).

Together, these observations expand our understanding of the SRP RNA in dikarya and reveal unexpected diversity of this noncoding RNA. Members of both phyla within the dikarya—Ascomycota and Basidiomycota—encode SRP RNAs with yeast-specific Alu domains that lack helices 3 and 4. Because basal fungi whose SRP RNAs have been identified, such as those in the phyla Zygomycota and Chytridiomycota, encode metazoan-like Alu domains (16,27), our findings suggest that a reduction of the Alu domain took place within a common ancestor of the dikarya. These changes are likely associated with alterations in the mode of RNA-protein binding in this region, since the *S. cerevisiae* ortholog of SRP14 does not bind the metazoan SRP RNA (33).

In contrast to the yeast-specific Alu domain features observed in basidiomycete SRP RNAs, the S domain alterations in this phylum likely occurred later, since they are present only in restricted lineages within Basidiomycota, and not at all within Ascomycota (Figure 3C). The insertions and deletions within helix 8 are present in a complex distribution suggestive of multiple gain and loss events during the evolution of Basidiomycota. Importantly, these alterations occur in the most conserved regions of the SRP RNA—the asymmetric and symmetric loops of helix 8—and sometimes affect bases previously thought to be invariant across SRP RNAs. Our newly identified sequences should therefore increase the sensitivity of structure-based SRP RNA searches, thereby allowing discovery and comparative analysis of this noncoding RNA in the heretofore unexplored fungal lineage, and perhaps in other eukaryotic lineages as well.

### SRP RNA alterations in basidiomycetes are associated with alterations in the RNA-binding domain of Srp54

**Figure 4.**
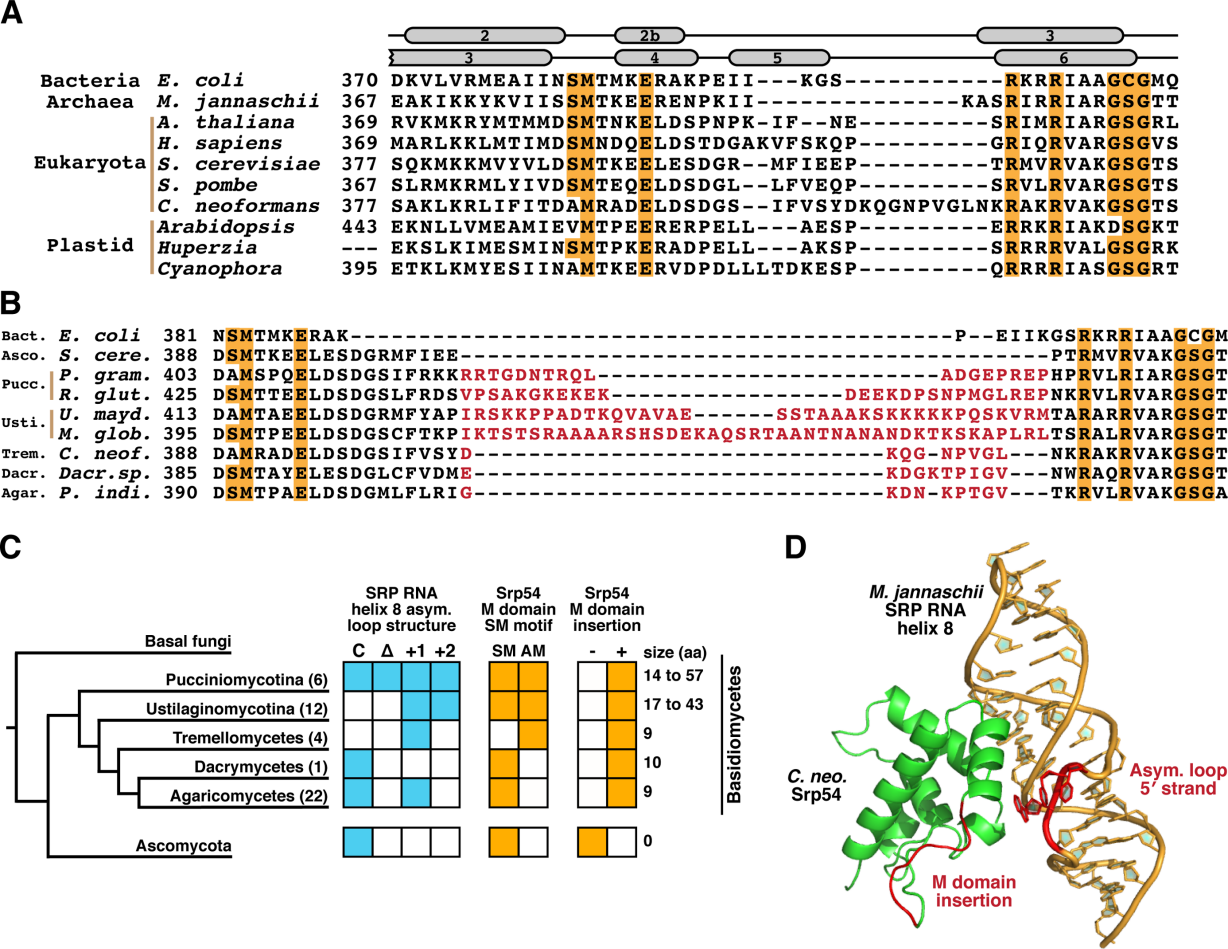
SRP RNA helix 8 alterations in Basidiomycota are accompanied by alterations in its protein binding partner, Srp54. (**A**) Alignment of SRP54/Ffh M-domain sequences from archaea, bacteria, eukaryotes and chloroplasts. Highly conserved residues involved in binding SRP RNA helix 8 are indicated in orange. Residues corresponding to alpha helices in the *E. coli* protein structure (19) are indicated above, top line. Residues corresponding to alpha helices in the human protein structure (20) are indicated above, bottom line. (**B**) Alignment of Srp54 M-domain sequences from basidiomycetous yeast. Highly conserved residues involved in binding SRP RNA helix 8 are indicated in orange. Residues inserted between the SM and GSG motifs in basidiomycetes, as compared to ascomycetes, are indicated in red. (**C**) Presence across Basidiomycota of SM motif mutations and M-domain insertions in Srp54. The number of inserted residues between the SM and GSG motifs relative to the *S. cerevisiae* Srp54 sequence is indicated at right. The number of full-length Srp54 sequences analyzed per subphylum and class is indicated in parentheses. The phylogenetic tree was adapted from (26). (**D**) Location of M-domain insertion in *C. neoformans* Srp54 relative to the asymmetric loop of SRP RNA helix 8. *C. neoformans* Srp54 was modeled on a structure of *M. jannaschii* SRP54 bound to SRP RNA helix 8 (PDB ID: 2V3C) (22). Red bases indicate the 5′ strand of the asymmetric loop, where the inserted helix in the *C. neoformans* SRP RNA would emerge. The red protein region indicates the site of inserted residues in *C. neoformans* Srp54.

Because the unusual features of the *C. neoformans* SRP RNA lie in a region that physically interacts with SRP54, we sought to examine this protein in Basidiomycota. SRP54 is the most conserved protein subunit of the SRP, and binds to SRP RNA helix 8 via its M domain. The structural basis for this interaction is similar in all domains of life (19,32,34), which explains the importance of several highly conserved residues in the M domain (Figure 4A).

Sequence alignment of the *C. neoformans* Srp54 with its archaeal, bacterial, chloroplast and other eukaryotic orthologs revealed several surprising M domain alterations (Figure 4A). First, the *C. neoformans* Srp54 contains an insertion in a region surrounded by *E. coli* Ffh helices 2b and 3. This region differs between eukaryotes and bacteria, with eukaryotes generally containing ∼5 additional residues that form a small helix (32). The *C. neoformans* protein contains an even larger insertion, to an extent not previously described in eukaryotes. To examine the phylogenetic distribution of this insertion in Basidiomycota, we computationally identified 46 additional *SRP54* genes throughout this phylum (Supplementary Dataset S2 and Figure 4B). Remarkably, all proteins had M domain insertions, ranging from 9 residues (*C. neoformans*, as compared to *S. cerevisiae*) to 57 residues (*M. violaceum*) (Supplementary Table S4 and Figure 4B and C).

A second striking feature of the *C. neoformans* Srp54 is the alteration of position 389 from serine to alanine (Figure 4A). This highly conserved residue contacts the SRP RNA, and mutations have not been observed in eukaryotic SRP54. However, alterations to alanine or valine have been observed in chloroplast SRP54 (Figure 4A) (17). Interestingly, mutations at this residue are sufficient to disrupt binding of the chloroplast SRP54 to the chloroplast SRP RNA (35), and tend to be found in plant species whose chloroplasts have lost the SRP RNA, suggesting a coevolution between the RNA and SRP54 in plastids (17,36–37). In this context it is notable that every basidiomycete encoding the S389A mutation was found to have a noncanonical SRP RNA asymmetric loop structure, raising the possibility that this mutation's presence requires compensatory RNA changes to preserve RNA binding. Furthermore, the S389A mutation is less widespread in Basidiomycota than is the M domain insertion, suggesting that its presence is not solely to compensate for the insertion's effects on RNA binding, or vice versa (Figure 4C).

Thus, two major alterations in the *C. neoformans* Srp54 are predicted to affect its binding to SRP RNA helix 8. Residue 389 is expected to directly contact bases of the symmetric loop, whereas the M domain insertion is expected to abut the SRP RNA near the 5′ strand of the asymmetric loop. In fact, a model of the *C. neoformans* Srp54 based on its *M. jannaschii* ortholog (22) predicts that the M domain insertion faces the SRP RNA in the region from which the novel helix 8 RNA insertion emerges (Figure 4D). This structural concordance supports the idea that the unusual features in Srp54 and the SRP RNA—which we observe widely in basidiomycetes but not in ascomycetes—arose in co-evolution with each other. Among the subphyla of Basidiomycota, however, there is no strict correlation between the Srp54 M domain insertion, the Srp54 S389A mutation, and the SRP RNA helix 8 asymmetric loop alterations. Thus, the co-evolution between these features may be complex or affected by additional, yet to be discovered variations of the SRP protein components in Basidiomycota.

Our results highlight the potential for a novel co-evolution in the fungal lineage between the most conserved components of SRP: Srp54 and SRP RNA helix 8. Although the forces affecting this co-evolution remain unclear, it is possible that an insertion in the SRP RNA helix 8 drove the acquisition of Srp54 protein alterations that accommodate it. We note that highly conserved RNA folds can be exploited as targets of toxins. The prokaryotic VapC toxin cleaves initiator tRNA at the anticodon loop (38), whereas Shiga and ricin family toxins target the 28S rRNA at one of its most conserved regions, present from bacteria to human (39,40). Given that basidiomycete life cycles typically take place in a soil environment (41), in the context of many other microorganisms, exposure to an unknown environmental toxin targeting the universally conserved SRP RNA helix 8 could conceivably have driven alterations in this region, leading to the unprecedented SRP RNA and protein changes observed in Basidiomycota. Alternatively, alterations of Srp54 that affect SRP activity, such as the M domain insertion, may have arisen in the basidiomycete common ancestor and subsequently driven changes in the SRP RNA that utilize the novel protein surface. This latter scenario may be consistent with the observation that the Srp54 M domain insertions are phylogenetically more widespread than are the SRP RNA helix 8 mutations. Further evaluation of these and additional scenarios awaits structural studies of the *C. neoformans* SRP as well as the construction of mutants that disentangle the effects of each individual basidiomycete SRP alteration on SRP function and RNA binding.

Finally, we note that the phylum Basidiomycota includes a number of human and plant pathogens, with *C. neoformans* alone causing over 650 000 world-wide deaths per year (42). Current antifungals against *Cryptococcus* are highly toxic and difficult to administer, and development of new agents has been slow, with only one new drug class—the echinocandins—introduced over the last 30 years (43). Our finding of structural SRP RNA features specific to Basidiomycota points to the SRP as a plausible new target for antifungal compound development. This approach is supported by previous success in pharmacologic targeting of bacterial-specific RNA structural elements in two other essential ribonucleoproteins: RNase P and the ribosome (44–47).

## Supplementary Material

SUPPLEMENTARY DATA
