# Supplementary material for: Noncanoncial signal recognition particle RNAs in a major eukaryotic phylum revealed by purification of SRP from the human pathogen Cryptococcus neoformans
Source: Nucleic Acids Res. 2015 Oct 10;43(18):9017–27. doi: 10.1093/nar/gkv819 (PMC4605306; doi:10.1093/nar/gkv819)
Supplement: SUPPLEMENTARY DATA [file supp_43_18_9017__index.html]

Noncanoncial signal recognition particle RNAs in a major eukaryotic phylum revealed by purification of SRP from the human pathogen Cryptococcus neoformans — Noncanoncial signal recognition particle RNAs in a major eukaryotic phylum revealed by purification of SRP from the human pathogen Cryptococcus neoformans — SUPPLEMENTARY DATA 

# Noncanoncial signal recognition particle RNAs in a major eukaryotic phylum revealed by purification of SRP from the human pathogen *Cryptococcus neoformans*

## SUPPLEMENTARY DATA

- SUPPLEMENTARY DATA
- SUPPLEMENTARY DATA
- SUPPLEMENTARY DATA
