# Supplementary material for: Noncanoncial signal recognition particle RNAs in a major eukaryotic phylum revealed by purification of SRP from the human pathogen Cryptococcus neoformans
Source: Nucleic Acids Res. 2015 Oct 10;43(18):9017–27. doi: 10.1093/nar/gkv819 (PMC4605306; doi:10.1093/nar/gkv819)
Supplement: SUPPLEMENTARY DATA [file supp_gkv819_nar-01320-r-2015-File006.pdf]

Supplementary Material for:

Noncanoncial signal recognition particle RNAs in a major eukaryotic phylum revealed by purification of SRP from the human pathogen *Cryptococcus neoformans*

Phillip A. Dumesic[1], Magnus A. Rosenblad[2], Tore Samuelsson[3], Tiffany Nguyen[1], James J. Moresco[4], John R. Yates III[4], and Hiten D. Madhani[1,*]

The Supplementary Material contains one Supplementary Figure and four Supplementary Tables.

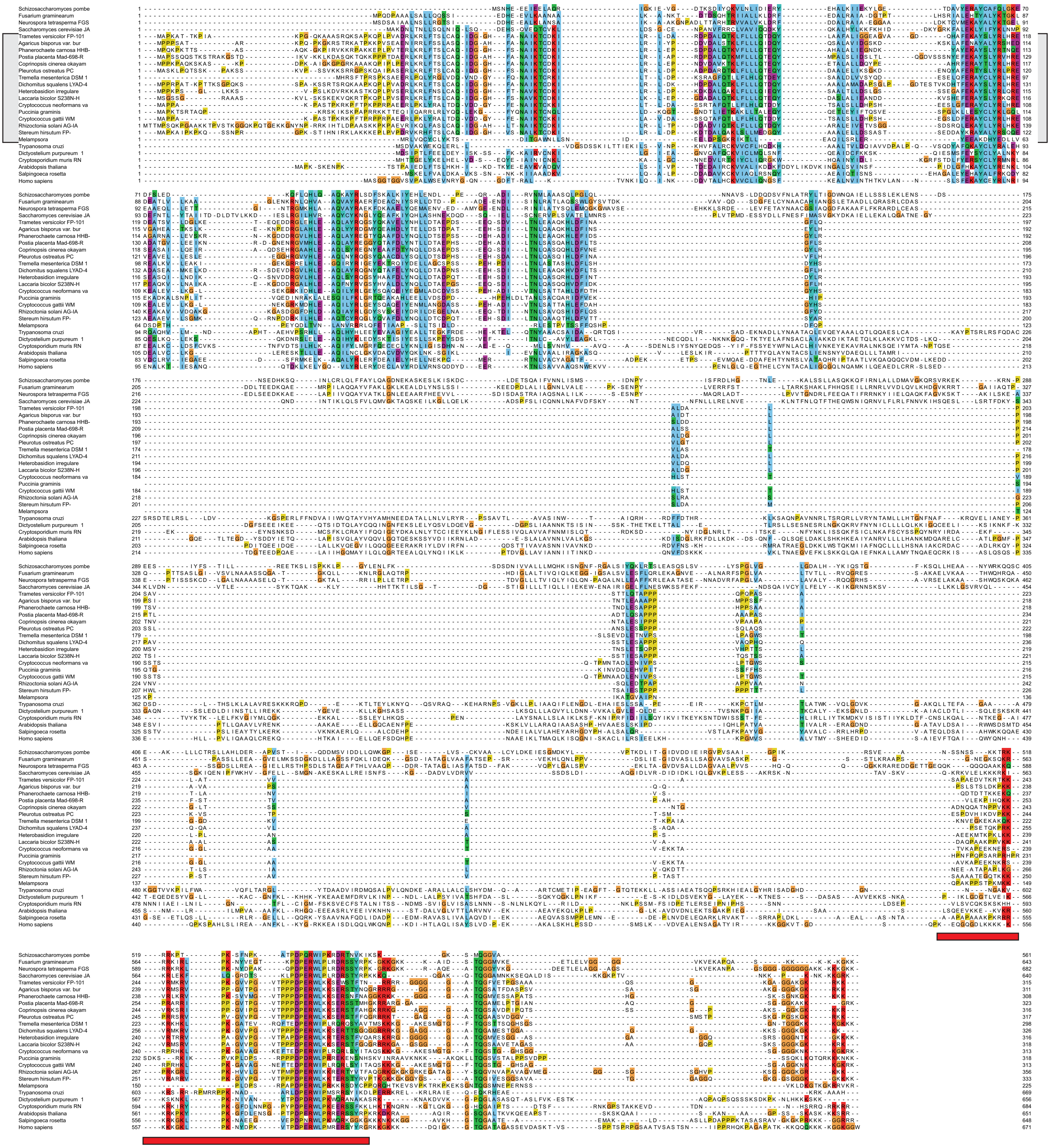
Schizosaccharomyces pombe
Fusarium graminearum
Neurospora tetrasperma FGS
Saccharomyces cerevisiae JA
Trametes versicolor FP-101
Agaricus bisporus var. bur
Phanerochaete carnosa HHB-
Postia placenta Mad-698-R
Coprinopsis cinerea okayam
Pleurotus ostreatus PC
Tremella mesenterica DSM 1
Dichomitus squalens LYAD-4
Heterobasidion irregulare
Laccaria bicolor S238N-H
Cryptococcus neoformans va
Puccinia graminis
Cryptococcus gattii WM
Rhizoctonia solani AG-IA
Stereum hirsutum FP-
Melampsora
Trypanosoma cruzi
Dictyostelium purpureum 1
Cryptosporidium muris RN
Arabidopsis thaliana
Salpingoeca rosetta
Homo sapiens

SUPPLEMENTARY FIGURE LEGENDS

Supplementary Figure S1. Alignment of eukaryotic SRP72 sequences

An alignment of SRP72 proteins from selected eukaryotes shows that a significant middle portion is missing in basidiomycetous yeast (gray box) as compared to all other groups. The C-terminal RNA-binding domain characteristic of SRP72 is highlighted with a red rectangle.

Supplementary Table S1. Strains used in this study

PAD#	CM#	Species	Genotype	Parental strain	Source
PAD2	CM229	*C. neo.*	H99 (wild-type)	-	1
PAD379	CM530	*C. neo.*	*srp19-CBP-2xFLAG-NatR*	PAD2	2
PAD382	CM533	*C. neo.*	*srp54-CBP-2xFLAG-NatR*	PAD2	2

1= Gift of J. Lodge
2= This study

Supplementary Table S2. Mass spectrometry results and background filtering criteria

Protein hits were excluded if they met at least one of the following criteria:
1) Identified by sequence coverage <10% (not shown) or spectrum count <20
2) Structural components of the ribosome
3) Proteins identified in purifications from a wild-type (untagged) strain
4) Proteins likely to be abundant, such as cytoskeletal proteins, metabolic proteins, chaperones, and mitochondrial proteins
The remaining proteins, which passed the filtering criteria, are highlighted.

Bait: WT (untagged)

Gene ID	Name	Spectrum count	% coverage	Exclusion Reason
CNAG_04994	Srp14	8	38.6	1
CNAG_00703	Rpl31a	3	31.1	1, 2
CNAG_04799	Rpl14a	2	24.5	1, 2
CNAG_04068	Rpl28a	3	24.4	1, 2
CNAG_01170	Rps17b	2	16.1	1, 2
CNAG_00656	Rpl7a	2	15.2	1, 2
CNAG_02578	Pch2	21	15.2	3
CNAG_04004	Rps1a	3	12.7	1, 2
CNAG_02174	Srp54	7	11.2	1
CNAG_04762	Rpl4a	5	11	1, 2

Bait: Srp19-CBP-2xFLAG (replicate 1)

Gene ID	Name	Spectrum count	% coverage	Exclusion Reason
CNAG_05240	Srp68	525	97.7	
CNAG_02174	Srp54	709	96.4	
CNAG_04797	Srp19	275	92.1	
CNAG_05798	Srp9/21	154	89.3	
CNAG_04994	Srp14	370	80.1	
CNAG_03708	Srp72	183	70.6	
CNAG_04799	Rpl14a	7	46.0	1, 2
CNAG_01976	Rpl23a	5	37.7	1, 2
CNAG_04068	Rpl28a	6	32.7	1, 2
CNAG_05556	Rpl38	3	31.8	1, 2
CNAG_02578	Pch2	20	30.1	3
CNAG_01170	Rps17b	2	29.4	1, 2
CNAG_06447	Rpl17a	5	22.0	1, 2
CNAG_04114	Rps0a	5	19.9	1, 2
CNAG_00703	Rpl31a	2	19.3	1, 2
CNAG_04004	Rps1a	3	18.5	1, 2
CNAG_01727	Hsp71	9	17.0	1, 2
CNAG_00656	Rpl7a	2	15.2	1, 2
CNAG_05232	Rml2	2	12.5	1, 2

CNAG_01332	Rps24a	2	11.9	1, 2
CNAG_03053	Rpl25	2	11.7	1, 2
CNAG_01927	Hsp40	7	11.7	1, 4
CNAG_01750	Hsp72	7	10.9	1, 4

Bait: Srp19-CBP-2xFLAG (replicate 2)

Gene ID	Name	Spectrum count	% coverage	Exclusion Reason
CNAG_05798	Srp9/21	178	95.7	
CNAG_05240	Srp68	854	90.3	
CNAG_02174	Srp54	807	85.9	
CNAG_04797	Srp19	325	75.8	
CNAG_04994	Srp14	416	68.4	
CNAG_03708	Srp72	172	53	
CNAG_04799	Rpl14a	10	46.0	1, 2
CNAG_02578	Pch2	26	41.5	3
CNAG_06447	Rpl17a	9	39.0	1, 2
CNAG_01727	Hsp71	18	38.4	1, 2
CNAG_01976	Rpl23a	7	33.3	1, 2
CNAG_04068	Rpl28a	9	32.7	1, 2
CNAG_01152	Rps6a	6	31.6	1, 2
CNAG_05232	Rml2	7	27.1	1, 2
CNAG_01750	Hsp72	9	23.5	1, 4
CNAG_01884	Rpl3	7	22.6	1, 2
CNAG_04114	Rps0a	5	22.6	1, 2
CNAG_00656	Rpl7a	4	22.0	1, 2
CNAG_04004	Rps1a	5	20.8	1, 2
CNAG_04011	Rpl43a	3	20.7	1, 2
CNAG_02359	Rps25a	2	20.6	1, 2
CNAG_00116	Rps3	3	19.8	1, 2
CNAG_04762	Rpl4a	5	18.5	1, 2
CNAG_03747	Rpl28	3	17.6	1, 2
CNAG_03780	Rps16a	3	17.1	1, 2
CNAG_04021	Rpl26a	3	15.8	1, 2
CNAG_00703	Rpl31a	2	14.3	1, 2
CNAG_00370	Rpl40a	2	14.0	1, 2
CNAG_06125	Tef1	3	13.9	1, 4
CNAG_03739	Rpl10	2	13.0	1, 2
CNAG_04448	Rpl19a	3	11.3	1, 2

Bait: Srp54-CBP-2xFLAG (replicate 1)

Gene ID	Name	Spectrum count	% coverage	Exclusion Reason
CNAG_02174	Srp54	605	98.1	
CNAG_05798	Srp9/21	90	92.1	

CNAG_05240	Srp68	226	86	
CNAG_04797	Srp19	133	84.5	
CNAG_04994	Srp14	162	78.9	
CNAG_03708	Srp72	93	64.5	
CNAG_05232	Rml2	6	22.0	1, 2
CNAG_06447	Rpl17a	9	21.4	1, 2
CNAG_01976	Rpl23a	2	21.0	1, 2
CNAG_03780	Rps16a	3	20.0	1, 2
CNAG_02578	Pch2	16	17.6	1, 3
CNAG_00116	Rps3	2	15.9	1, 2
CNAG_01727	Hsp71	8	14.8	1, 4
CNAG_00703	Rpl31a	9	14.3	1, 2
CNAG_04883	Rps18a	2	11.0	1, 2
CNAG_04004	Rps1a	6	10.8	1, 2
CNAG_01750	Hsp72	5	10.3	1, 4

Bait: Srp54-CBP-2xFLAG (replicate 2)

Gene ID	Name	Spectrum count	% coverage	Exclusion Reason
CNAG_05798	Srp9/21	130	95.7	
CNAG_02174	Srp54	1548	94.9	
CNAG_05240	Srp68	595	91.2	
CNAG_04797	Srp19	225	79.4	
CNAG_04994	Srp14	415	67.8	
CNAG_01727	Hsp71	60	66.4	4
CNAG_03708	Srp72	165	63.9	
CNAG_03747	Rpl28	6	48.6	1, 2
CNAG_06447	Rpl17a	11	42.9	1, 2
CNAG_01884	Rpl3	20	42.8	2
CNAG_00656	Rpl7a	11	42.8	1, 2
CNAG_01750	Hsp72	41	40.8	4
CNAG_04004	Rps1a	10	39.8	1, 2
CNAG_01152	Rps6a	13	39.7	1, 2
CNAG_02578	Pch2	30	38.8	3
CNAG_00703	Rpl31a	8	37.8	1, 2
CNAG_04114	Rps0a	12	37.7	1, 2
CNAG_01170	Rps17b	5	36.4	1, 2
CNAG_04762	Rpl4a	12	32.5	1, 2
CNAG_04799	Rpl14a	14	30.2	1, 2
CNAG_05556	Rpl38	6	29.9	1, 2
CNAG_00640	Rps4a	6	26.6	1, 2
CNAG_00116	Rps3	4	25.8	1, 2
CNAG_05232	Rml2	10	25.5	1, 2
CNAG_04021	Rpl26a	3	24.8	1, 2
CNAG_06633	Rsm19	2	24.7	1, 2

CNAG_03198	Rps8a	3	24.5	1, 2
CNAG_00334	Hsp75	12	23.6	1, 4
CNAG_03944	Ydj1	6	23.4	1, 4
CNAG_00771	Rpl35a	3	22.8	1, 2
CNAG_03053	Rpl25	6	20.8	1, 2
CNAG_03739	Rpl10	3	20.4	1, 2
CNAG_05525	Rps26a	4	18.9	1, 2
CNAG_04726	Rpl20a	2	18.6	1, 2
CNAG_03283	Rpl24a	2	18.0	1, 2
CNAG_03510	Rpl36a	2	18.0	1, 2
CNAG_04068	Rpl28a	8	17.9	1, 2
CNAG_03787	Tub1	4	16.3	1, 4
CNAG_06125	Tef1	8	15.9	1, 4
CNAG_06605	Rps2	3	15.2	1, 2
CNAG_05800	Rpl33a	2	15.2	1, 2
CNAG_02331	Rps9a	2	15.0	1, 2
CNAG_04448	Rpl19a	2	14.9	1, 2
CNAG_06150	Hsp82	6	14.3	1, 4
CNAG_01976	Rpl23a	4	13.8	1, 2
CNAG_00672	Rps11a	2	13.7	1, 2
CNAG_06231	Rpl16a	2	11.6	1, 2
CNAG_04445	Rps7a	2	11.6	1, 2
CNAG_06919	Nop1	2	11.5	1, 4
CNAG_01896	Ypr1	2	10.8	1, 4

Supplementary Table S3. Primers used in this study

Name	Sequence	Target	Location	Application
Linker-1	(5rApp)CTGTAGGCACCATCAAT(3ddC)*	-	-	3' linker
PAD2114	ATTGATGGTGCCTACAG	-	-	RNA cloning RT primer
PAD2115	TCTACAGTCCGACGATCrGrGrG**	-	-	RNA cloning template switch
PAD2114	ATTGATGGTGCCTACAG			
PAD2116	GTTCTACAGTCCGACGATC	-	-	RNA cloning PCR
PAD2269	CGTTACCGGCCTATCTGTC	*SRP RNA*	-	5' RACE RT primer
PAD2287	GACTCGAGTCGACATCGATTTTTTTTTTTTTTTTT	-	-	5' RACE adaptor
PAD2288	GACTCGAGTCGACATCG	-	-	5' RACE PCR primer
PAD2269	CGTTACCGGCCTATCTGTC	*SRP RNA*	-	5' RACE PCR primer
PAD913	ACCTTCGGGTCGGCATAAGA	*U6 snRNA*	-	RNA-IP qPCR
PAD914	TCCTCTCTGCTCGAGTTTGTC			
PAD1765	CCAGATCATGTTCGAGACTTTC	*CNAG_0483 (actin)*	exon 5	RNA-IP qPCR
PAD1766	CCAGAGTCAAGAACGATACCG			
PAD1761	GCAAGGTCATTCCTTCCCTTA	*CNAG_6699 (GAPDH)*	exon 10	RNA-IP qPCR
PAD1762	ACCCTTCTCAATGCGACAAAC			
PAD2265	GGGGAAGGTGCTCTTTTAC	*SRP RNA*	-	RNA-IP qPCR
PAD2267	CATGAGATCTTGGTGGGACTC			
PAD2205	GTATGCCAAACCAGCCAGAATGATCCGTTACCGGCC	*SRP RNA*	-	Northern probe

*(5rApp) = 5'adenylation
*(3ddC) = 3'dideoxycytosine
**rG = riboguanosine

Supplementary Table S4. Predicted structural features of SRP RNA and Srp54 in Basidiomycota

Species for which SRP RNA or Srp54 sequences were analyzed for this study are listed. Predicted Srp54 protein sequences were aligned (SI Dataset S2) and assessed for alterations in the M domain including mutation of the SM motif and insertions between the SM and GSG motifs. The M domain insert size is relative to the *S. cerevisiae* sequence. For some species, genomic sequences corresponding to individual M domain motifs could be identified and assessed despite the fact that full-length Srp54 sequences could not be identified. For the *T. asahii* Srp54, two values are reported for M domain insert length since the Srp54 gene model was ambiguous between two sequences: one with a 9-residue insertion and one with an 8-residue deletion. SRP RNA structures were predicted using UNAFold and assessed for the presence of helix 8 asymmetric loop features including canonical structure (0), single helix insertion (1), dual helix insertion (2), or deletion in the 5′ strand such that the 3′ asymmetric loop strand becomes the long strand (Δ). Organisms in which the helix 8 symmetric loop contains a 1 nt insertion in the 3′ strand, thereby rendering it asymmetric, are noted. The genomes of some species encoded multiple putative SRP RNA genes, as indicated in parentheses.

Species	Full-length Srp54 assessed	Srp54 SM motif	Srp54 M domain indel	SRP RNA assessed	Asym. loop helices	Sym. loop 1 nt insert
Ustilaginomycotina						
Ustilago_maydis	+	AM	37	+	2	-
Sporisorium_reilianum	+	AM	38	+	1	-
Pseudozyma_antarctica	+	AM	39	+	2	-
Malassezia_globosa	+	SM	43	+	1	-
Malassezia_sympodialis	+	SM	17	+	1	-
Tilletiaria_anomala	+	SM	41	+	1	-
Pleurozyma_flocculosa	+	AM	42	-	-	-
Melanopsichium_pennsylvanicum	+	AM	38	-	-	-
Pseudozyma_brasiliensis	+	AM	37	-	-	-
Ustilago_hordei	+	AM	39	-	-	-
Pseudozyma_aphidis	+	AM	39	-	-	-
Pseudozyma_hubeiensis	+	AM	38	-	-	-

Pucciniomycotina						
Puccinia_striiformis	-	-	18	+	2	+
Puccinia_graminis	+	AM	18	+	2	+
Melampsora_laricis-populina	+	AM	18	+	1	+
Mixia_osmundae	+	AM	14	+	Δ	+
Rhodosporidium_toruloides	+	SM	28	+	Δ	-
Rhodotorula_glutinis	+	SM	26	+	Δ	+
Microbotryum_violaceum	+	SM	57	+	1	+
Heterogastridium_pycnidioideum	-	-	29	+	0	+
Naiadella_fluitans	-	-	26	+	0	-
Agaricostilbum_hyphaenes	-	-	27	+	0	-
Basidiomycota incertae sedis						
Wallemia_sebi	+	SM	9	+	0	-
Wallemia_ichthyophaga	+	SM	9	+	0	-
Tremellomycetes						
Kwoniella_mangrovensis	-	AM	9	+	1	+
Tremella_mesenterica	+	AM	9	+	1	+
Cryptococcus_neoformans	+	AM	9	+	1	+
Cryptococcus_gattii	+	AM	9	+	1	+
Trichosporon_oleaginosus	-	AM		+ (2)	1	+
Trichosporon_asahii	+	AM	9/-8	+	1	+
Dioszegia_cryoxerica	-	AM	28	+ (2)	1	-
Cryptococcus_vishniacii	-	AM	9	+	1	-
Cryptococcus_pinus	-	AM	9	+	1	+
Cryptococcus_dejecticola	-	AM	9	+	1	+
Cryptococcus_bestiolae	-	AM	9	+	1	+
Cryptococcus_flavescens	-	-	-	+	1	+

Dacrymycetes						
Calocera_viscosa	-	-	-	+	0	-
Calocera_cornea	-	-	-	+	0	-
Dacryopinax_sp	+	SM	10	+	0	-
Agaricomycetes						
Sistotremastrum_suecicum	-	-	-	+ (2)	0	-
Sistotremastrum_niveocremeum	-	-	-	+ (2)	0	-
Sebacina_vermifera	-	-	-	+	0	-
Piriformospora_indica	+	SM	9	+	1	-
Stereum_hirsutum	+	SM	9	+	0	-
Heterobasidion_irregulare	+	SM	9	+	0	-
Heterobasidion_annosum	-	-	-	+	0	-
Wolfiporia_cocos	-	-	-	+	0	-
Trametes_versicolor	+	SM	9	+	0	-
Trametes_ljubarskyi	-	-	-	+	0	-
Postia_placenta	-	-	-	+	0	-
Trichaptum_abietinum	-	-	-	+	0	-
Pycnoporus_sanguineus	-	-	-	+	0	-
Pycnoporus_coccineus	-	-	-	+ (2)	0	-
Pycnoporus_cinnabarinus	-	-	-	+	0	-
Polyporus_arcularius	-	-	-	+	0	-
Lignosus_rhinocerotis	-	-	-	+	0	-
Fibroporia_radiculosa	-	-	-	+	0	-
Dichomitus_squalens	+	SM	9	+	0	-
Cerrena_unicolor	-	-	-	+ (2)	0	-
Phlebiopsis_gigantea	-	-	-	+	0	-
Obba_rivulosa	-	-	-	+	0	-
Ceriporiopsis_subvermispora	+	SM	9	+	0	-
Bjerkandera_adusta	-	-	-	+	0	-

Lentinus_tigrinus	-	-	-	+	0	-
Laetiporus_sulphureus	-	-	-	+	0	-
Ganoderma_lucidum	-	-	-	+	0	-
Fomitopsis_pinicola	+	SM	9	+	0	-
Daedalea_quercina	-	-	-	+ (2)	0	-
Clavicorona_pyxidata	-	-	-	+ (2)	0	-
Antrodia_sinuosa	-	-	-	+ (2)	0	-
Ramaria_rubella	-	-	-	+ (2)	0	-
Sphaerobolus_stellatus	-	-	-	+ (3)	0	-
Phellinus_noxius	-	-	-	+	0	-
Fomitiporia_mediterranea	+	SM	9	+	0	-
Neolentinus_lepideus	-	-	-	+	0	-
Gloeophyllum_trabeum	+	SM	9	+	0	-
Punctularia_strigosozonata	+	SM	9	+	0	-
Phlebia_brevispora	-	-	-	+	0	-
Phanerochaete_chrysosporium	-	-	-	+	0	-
Phanerochaete_carnosa	+	SM	9	+	0	-
Schizopora_paradoxa	-	-	-	+	0	-
Dendrothele_bispora	-	-	-	+	0	-
Cylindrobasidium_torrendii	-	-	-	+ (3)	0	-
Tulasnella_calospora	-	-	11	+	0	-
Rhizoctonia_solani	3	SM	9	+	0	-
Botryobasidium_botryosum	-	-	-	+	0	-
Auricularia_subglabra	-	-	-	+	0	-
Jaapia_argillacea	+	SM	9	+	0	-
Suillus_luteus	-	-	-	+	0	-
Suillus_brevipes	-	-	-	+	0	-
Rhizopogon_vinicolor	-	-	-	+	0	-
Scleroderma_citrinum	-	-	-	+	0	-
Pisolithus_tinctorius	-	-	-	+ (2)	0	-
Pisolithus_microcarpus	-	-	-	+	0	-
Hydnomerulius_pinastri	-	-	-	+	0	-
Paxillus_rubicundulus	-	-	-	+	0	-

Paxillus_involutus	-	-	-	+ (2)	0	-
Gyrodon_lividus	-	-	-	+	0	-
Serpula_lacrymans	2	SM	9	+	0	-
Coniophora_puteana	+	SM	9	+	0	-
Boletus_edulis	-	-	-	+	0	-
Leucogyrophana_mollusca	-	-	-	+	0	-
Piloderma_croceum	-	-	-	+ (2)	0	-
Tricholoma_matsutake	-	-	-	+	0	-
Rickenella_mellea	-	-	-	+	0	-
Panellus_stipticus	-	-	-	+	0	-
Laccaria_bicolor	+	SM	9	+	0	-
Laccaria_amethystina	-	-	-	+ (2)	0	-
Gymnopus_luxurians	-	-	-	+ (2)	0	-
Gymnopus_androsaceus	-	-	-	+ (3)	0	-
Hypholoma_sublateritium	-	-	-	+	0	-
Galerina_marginata	+	SM	9	+	0	-
Schizophyllum_commune	+	SM	9	+	0	-
Coprinopsis_cinerea	+	SM	9	+	0	-
Volvariella_volvacea	-	-	-	+	0	-
Pleurotus_ostreatus	+	SM	9	+	0	-
Guyanagaster_necrorhiza	-	-	-	+	0	-
Flammulina_velutipes	-	-	-	+	0	-
Armillaria_mellea	-	-	-	+ (2)	0	-
Omphalotus_olearius	-	-	-	+	0	-
Moniliophthora_roreri	+	SM	9	+	0	-
Moniliophthora_perniciosa	-	-	-	+	0	-
Fistulina_hepatica	-	-	-	+	0	-
Hebeloma_cylindrosporum	-	-	-	+	0	-
Gymnopilus_chrysopellus	-	-	-	+ (2)	0	-
Cortinarius_glaucopus	-	-	-	+	0	-
Amanita_thiersii	-	-	-	+	0	-
Amanita_muscaria	-	-	-	+	0	-
Amanita_jacksonii	-	-	-	+	0	-

Plicaturopsis_crispa	-	-	-	+	0	-
Macrolepiota_fuliginosa	-	-	-	+	0	-
Leucoagaricus_gongylophorus	-	-	-	+	0	-
Agaricus_bisporus	2	SM	9	+	0	-
Kwoniella_heveanensis	-	-	-	+	1	-
Artolenzites_elegans	-	-	-	+	0	-
