# Supplementary material for: Noncanoncial signal recognition particle RNAs in a major eukaryotic phylum revealed by purification of SRP from the human pathogen Cryptococcus neoformans
Source: Nucleic Acids Res. 2015 Oct 10;43(18):9017–27. doi: 10.1093/nar/gkv819 (PMC4605306; doi:10.1093/nar/gkv819)
Supplement: SUPPLEMENTARY DATA [file supp_gkv819_nar-01320-r-2015-File007.rtf]

Supplementary Dataset S1.  Basidiomycete SRP RNA genes identified in this study>Cryptococcus_bestiolae DB=GCA_000512585.1_Cryp_best_CBS10118_V1_genomic.fna ACC=KI894021.1 REGION=1094376-1094681 TAX=Agaricomycotina; Tremellomycetes; Tremellales; mitosporic Tremellales; CryptococcusACGCTGTAATGGCTTTCGGGAAGGTGTTTATTTATCCTCACGGAACTCCTCTCCTTAGCGCTCCATCATTACGTTATATATGTCTCCTCTAGCATCAGTGTTCGAGTGCCACCAAAGTCTCATGACTTTGCGTATTTATGGCTCTTACTCGGTAATCAAGTTAGTTTTACAGATGATCCTCAAAAGGGATTCGTCTGGCGAATAGGCCGGTGACGGATCAATTCGACTAATCTTGGCATTGATACTACCAGAGGATTGGACGGACGTAGGTTAGCGCAGGGAGAGCCGGGTGAACACCAACTTTTT>Cryptococcus_flavescens DB=GCA_000442785.1_Cf_30_300r_Split10plusN_genomic.fna ACC=CAUG01000100.1 REGION=5028-5192 TAX=Agaricomycotina; Tremellomycetes; Tremellales; Tremellales incertae sedis; CryptococcusAAGCTGTAATGGCTTTTGAGAAGATGTCCACTTACTACCACGGATCAACCCTCATCAGCGCTCCATCATTACACTTTCAGTCTCCTCTAGCACGAGTGTTCGAGCCCCACCTTACTCTCAAGAGTGAGCTTGTTGCTTCGCTCGGTGATCAAATGGATTATGGTCGTACTTGTGCGACTCAGATAGGCCGGCAACGGATCATTCTGGTTCATGTTGGCATTGGTGCTTCAAGAGGATGGGACGGGTGTAGGCAGCGCAGTGAGGTCCGGGTGGGGATCGACTTTT>Cryptococcus_gattii DB=GCA_000149475.1_ASM14947v1_genomic.fna ACC=CH408163.1 REGION=719660-719940 TAX=Agaricomycotina; Tremellomycetes; Tremellales; Tremellaceae; Filobasidiella; Filobasidiella/Cryptococcus neoformans species complexATGCTGTAATGGCTTAGGGGAAGGTGCCCTTTTATTTTCACGGAGTTCCTCTCTTCAGCGCTGCCACATTGCGGCCGCGTCTCCTCTAGCCCGTGTGTTCGAGTCCCACCAAGATCTCATGATTTTGTCTTTGGGTTTACTCGGTAATCAAGCCAGTTCTCATTTCTCGTAAATGACAGATAGGCCGGTAACGGATCATTCTGGCTGGTTTGGCATACATGCTTCAAGGGGATTGGACAGTCGTAGCAGCGCAGAGAGAGCCGGGAGGACACCAACTTTTT>Cryptococcus_pinus DB=GCA_000512605.1_Cryp_pinu_CBS10737_V1_genomic.fna ACC=KI894011.1 REGION=1277793-1278111 TAX=Agaricomycotina; Tremellomycetes; Tremellales; mitosporic Tremellales; CryptococcusACGCTGTAATGGCTTTTGGGAAGGTGTTAATTTGTTATCACGGATTTCCTCTCTTTAGCGCTCCATCATTACGTTATTTAAGTCTCCTCTAGCATCAGTGTTCGAGTCCCATCATAATCTCATGATTGTGTCTCCCTTTAATGGGAATTAAATGGCTCTTACTCGGTAATCAAGTTAGTAATTTAATACTTCTTATGATGTATTACGAATAGGCCGGTAACGGATCAATTCGACTATATTTGACATTGAGGCTACCAGAGGATAGGACAAACGTAGGAAGCGTAAGGAGTGCCGGGTCAACACCAACTTTTTGACTTTT>Cryptococcus_neoformans DB=GCA_000149245.3_CNA3_genomic.fna ACC=CP003820.1 REGION=801198-801479 TAX=Agaricomycotina; Tremellomycetes; Tremellales; Tremellaceae; Filobasidiella; Filobasidiella/Cryptococcus neoformans species complexATGCTGTAATGGCTTAGGGGAAGGTGCTCTTTTACTTTCACGGAGTTCCTCTCTTCAGCGCTGCCACATTGCGGCCACGTCTCCTCTAGCCTGTGTGTTCGAGTCCCACCAAGATCTCATGATTTTGTCTTTGGGTTTACTCGGTAATCAAGCCAGTTTTCATTTCTCGTAAATGACAGATAGGCCGGTAACGGATCATTCTGGCTGGTTTGGCATACATGCTTCAAGAGGATTGGACAGTCGTAGCAGGGCAGAGAGAGCCGGGAGGACACCAACTTTTTT>Kwoniella_heveanensis DB=GCA_000507425.2_Cryp_heve_CBS569_V2_genomic.fna ACC=ASQC01000111.1 REGION=100416-100728 TAX=Agaricomycotina; Agaricomycetes; Agaricomycetes incertae sedis; Polyporales; Coriolaceae; TrametesACGCTGTAATGGCTTTTGGGAAGGTGTTGATTTACTCTTACGGACCTCCTCTCTTCAGCGCTCCATCATTACGTACCTATGTCTCCTCTAGCATCAGTGTTCGAGTCCCAGCAGAGTCTCATGACTTTGTGAAGTATTATGCTTCGTAAATGGTTTAAACACTTGGTGATTAGGTCAGTATTACTCCAGCCTGGCTGGAGTCAGATAGGCCGGCAACGGATCAATCTGGCTGATTTAGCTCTGGTGCTTATCGAGGATTGGACAGGCGTAGGCATGCGCAGAGAGAGCCGGGTTGACACCGACTTGTTTTTTT>Cryptococcus_dejecticola DB=GCA_000512565.1_Cryp_deje_CBS10117_V1_genomic.fna ACC=KI894031.1 REGION=752223-752531 TAX=Agaricomycotina; Tremellomycetes; Tremellales; mitosporic Tremellales; CryptococcusACGCTGTAATGGCTTTTGGGAAGGTGTTAATTTGTTATCACGGATCTCCTCTCTTTAGCGCTCCATCATTACGTTATATGTCTCCTCTAGCATCAGTGTTCGAGTCCCAGCATGATCTCATGATTGTGTATCCCTTCCGGGAATTTAAATGGCTCTCTTTACTCGGTAATCAAGTTAGTAATTTATCGTCGTTCGACGATAGCGAATAGGCCGGCAACGGATCAATTCGACTATACTTGACATTGAGGCTACCAGAGGATAGGACAGGCGTAGGAAGCGCAGAGAGTGCCGGGTTGACACCAACTTTTT>Kwoniella_mangrovensis DB=GCA_000507885.1_Kwon_mang_CBS10435_V2_genomic.fna ACC=KI669459.1 REGION=765282-765596 TAX=Agaricomycotina; Tremellomycetes; Tremellales; Tremellales incertae sedis; KwoniellaACGCTGTAATGGCTTTTGGGAAGGTGTTTATTTATCCTCACGGAACTCCTCTCTTTAGCGCTCCATCATTACGTTTCAAAGTCTCCTCTAGCATCAGTGTTCGAGTCCCACCAAAGTCTCATGATTTTGTGTCCCCCACGGGATGTTATGGCTCTTGCTCGGTAATCAAGTTAGTTCTACAAATCACTTCCAAAAGAGGTGGTCTGGCGAATAGGCCGGCAACGGATCAATTCGACTAATCTTGGCATTGATACTACCAGAGGATAGGACAGACGTAGGTTAGCGCAGGGAGAGCCGGGTGAACACCAACTTTTT>Trichosporon_asahii DB=GCA_000293215.1_Trichosporon_asahii_1_genomic.fna ACC=JH977607.1 REGION=1745641-1745912 TAX=Agaricomycotina; Tremellomycetes; Tremellales; mitosporic Tremellales; TrichosporonCAACTGTAATGGTTTAGGGAAGGTACGAGTTTTCATCACGGACTTCCTTGCTCCAGCGCGCCCTTTGTACAATTTGTCTCCACTAGCACCGGTGTTCGAACCCCACCTGAGTCTCATGACTTAGCGTTGGTTATCGTTTCGGTGATCATGCTAGTATGCCTCTGGCACTGACAGGCCGGCAACGGATCAGTCAGGCTAGTGTGGCATCGGTGCTCCAAGTGGATAGGATGGTTGTAGGCGCGCAGGGCAAGCCGGGCCCGTACCAACTTTTT>Tremella_mesenterica DB=GCA_000271645.1_Treme1_genomic.fna ACC=JH711530.1 REGION=83230-83516 TAX=Agaricomycotina; Tremellomycetes; Tremellales; Tremellaceae; TremellaCCGCTGTAGCGGCTTTCGGGAAGGTGTTTATCTAACTCACGGACTTACACTCGTCAGCGCTCCAATTAGACAATTACGTCTCCTCTAGCACCAGTGTTCGAACTCCACCGTATCATCATTGATACGTCTGTTGGTCTCACGTTCGGTGATCAGCTCGGTTTACACCCTGCATTGGGTGGCAGGCAGGCCGGTAACGGAGCAGCCTGGCCGACGTTGGCATTGGTGCTTCAAGAGGATAGGACGGTTGTCGGTAGCGCAGCGAGTGCCGGGTGGGCACCAACTCTTTT>Agaricus_bisporus DB=Abisporus_varburnettii.v2.unmasked.fasta ACC=scaffold_8 REGION=551321-551586 TAX=Agaricomycotina; Agaricomycetes; Agaricomycetidae; Agaricales; Agaricaceae; AgaricusCGGCTGTAGCGGCCCAATCCGGAATGAGCGGGGTTCCTCTCAGCGGGTTAAGCCATCAGTGCGCTGGTTCAAGACTGTCCCCTCCAGAAAACTTGGTTTCCACAGACCCTCGCTCCGTAAGGAGTGATCCGGACTTGGAATAATTTGTCCTTGACGGGCCAGGGAAGCTAATTCCGCAACCTGCAGGACTTGCCTAGTTAGTCTTCGGAGATCAGGGCATGTCGTCAGTCGCGCAATGGCTCGCGCCTCGCTCAAACCTTTTTACT>Amanita_jacksonii DB=GCA_000497225.1_AmaJack1.0_genomic.fna ACC=KI547035.1 REGION=265776-266038 TAX=Agaricomycotina; Agaricomycetes; Agaricomycetidae; Agaricales; Amanitaceae; AmanitaCGGTTGTGACGGCCGCAATTCGGGAATGCGTGGGATTCTTCTCATCGACCCCAACCGCAAGTGCGCTGGTTCAAGACTGTCCCCTCCAGAAAACTTGGTTTCCACAGTTCCTCGCTCCGAAAGGAATGATCCGAACTTGGAATGATTTGTCCTTGACAGGCCAGGGAAGCAATTCCGCAACCTGCTGGACTCGCCGAGTTAGTCTTCGGAGACCAGGACAGTTCGTCAGTCGCGCAGCGGTTCGAGTTCCTCGCAAACCTTTTT>Botryobasidium_botryosum DB=Botbo1_AssemblyScaffolds.fasta ACC=scaffold_5 REGION=1046569-1046833 TAX=Agaricomycotina; Agaricomycetes; Cantharellales; Botryobasidiaceae; BotryobasidiumGGGCTGTAACGGCCCTCACTCGGGAATGTGTCGGGTTCACCCTAGCGCCTATTCTACGCGCGCAAGTTCGAGGCTGCCCCCTTGCGACTTCTTGGTTTCAACTTTCACTCGGGCCGCAAGGCCTGCTGCTGAGATTGAATAATCGGTACTCCGCGAGCCAGGGAAGCAATTCCGCAACTCAGAGTACTTGCCGAGATCGTCTTCAAAGACAAGGGCATGTCCGCTGTCGCGCGGGTCAAGCGCGCTCGTCACAAACTCACCTCTT>Ceriporiopsis_subvermispora DB=Ceriporiopsis_subvermispora.unmasked.fasta ACC=scaffold_13 REGION=595591-595864 TAX=Agaricomycotina; Agaricomycetes; Polyporales; Meruliaceae; CeriporiopsisTGGCTGTAGCGGCCCTAATCCGGGAATGCGAGGTGGTCTAAAGCCCCAACGGATAAACCCGTCTGTGCGCTGGTTCAAGACCGTCCCCTCCAGAAAACTTGGTAGCCTTCGGCTGTCCGCCGCGCAAGTGGTGGTATGGCCTGGCGTAATCTGTCCTGCACGGGCCAGGGAAGTAATTCCGCAACCCACAGGATAGGCCGAGTTAGTCTTAGGAGATCAGGGCGAGTCGTCTGATCGCGCGGATGGGCCGTGCCCTTCGTAAACCTTTTGCATT>Coniophora_puteana DB=Conpu1_AssemblyScaffolds.fasta ACC=scaffold_2 REGION=2768275-2768540 TAX=Agaricomycotina; Agaricomycetes; Agaricomycetidae; Boletales; Coniophorineae; Coniophoraceae; ConiophoraCGGCTGTAATGGCCCTACAGCGGGAATGCGAGGTGGTTCTTCCAGCGGCCAACTCTGCGAGTGCGCTGTTTCAAGTCTGCCCCCTCCAGAAAACTTGGTCTCCACAGACTCTCACGCCGCAAGGCATGATCGGTCTTGGAATAATTGGTTCTAGACTCGCCAGGGAAGCAATTCCGCAACGGGCAGAGCCTGCCGAGTTAGTCGTAGGAGATAAGGGTAGTGCTGCAGTCGCGCAGCAGGGCGTGCCTCTCGCAAACCTTTTGCTT>Coprinopsis_cinerea DB=Coprinopsis_cinerea.unmasked.fasta ACC=Chr_2 REGION=2773747-2774009 TAX=Agaricomycotina; Agaricomycetes; Agaricomycetidae; Agaricales; Psathyrellaceae; CoprinopsisCGGCTGTCATGGCCGCAACTCGGGAATGCGGACGCTTTATCTCAGCGGGTTCTCCATCAGTGCGCTGGTTCAAGACTGTCCCCTCCAGAAAACTTGGTTTCCACAGACCCTCGCGCCGTAAGGTGTGATTTGGTCTTGGAATAATTTGTCCTTGACTGGCCAGGGAAGCAATTCCGCAACCTGCAGGACTTGCCGAGTTAGTCTTCGGAGATCAGGGCATGTCGTCAGTCGCGCAGTGGAACGCGTCGTCCGTAAACCTTTTT>Dichomitus_squalens DB=Dicsq1_AssemblyScaffolds.fasta ACC=scaffold_9 REGION=836381-836652 TAX=Agaricomycotina; Agaricomycetes; Polyporales; Polyporaceae; DichomitusTGGCTGTAATGGCCCTCAACCGGGAATGTGAGGTGGTCTAAAGTCCCAACGGAACAAGTCGTTCGTGCGCTGGTTCAAGACCGTCCCCTCCAGAAAACTTGGTTACCTATCGTCGCCCCTACCGTAAGGCGGGGATTGACCCGGTATAATACGTCCTTGACTGGCCAGGGAAGCAATTCCGCAACCTGCAGGACGTGCCGAGTTAGTCTTAGGAGATCAGGACGTGTCGTCTGTTCGCGCGAATGACCCGTGCCCCTCATAAACCCTTACTT>Fibroporia_radiculosa DB=Fibra1_AssemblyScaffolds.fasta ACC=scaffold_22166 REGION=55344-55617 TAX=Agaricomycotina; Agaricomycetes; Polyporales; Polyporaceae; FibroporiaTGGCTGTAGCGGCCCTATTCCGGGAATGTGAGGTGGTCTAAAGCTTCAACGGGTTCACTCGTTTGTGCGCTGGTTCAAGACCGTCCCCTCCAGAAAACTTGGTGACTTTGACTGTCCACCGCGGAAGCGGAGACATGGTCTGGTGTAATCCGCCCTTGACTGGCCAGGGAAGCAATTCCGCAACCTGCAGGGTGTGCCGAGTTAGTCTTAGGAGATAAGGACGAGTCGTCTGTTCGCGCAAATGGGCCGTGCTCTTCACAAACCTTTTTGGCTT>Flammulina_velutipes DB=GCA_000633125.1_Fv1.0_genomic.fna ACC=CM002703.1 REGION=863335-863597 TAX=Agaricomycotina; Agaricomycetes; Agaricomycetidae; Agaricales; Physalacriaceae; FlammulinaCGGCTGTAATGGCTCAACCGGGAATGTACTGGGTTATGTCAGCGAATCACTTCGCCTGTGCGCTGGTTCAAGACTGTCTCCTCCAGAAAACTTGGTGTCCACAGACTCCCGCAGCGTAAGCCGTGGGTCGGTCTTGGAATAATCAGTCCATGACGGGCCAGGGAAGCAATTCTGCAACCCGCTGGACTGGCCGAGTTAGTCGTAGGAGATTGGGACACGTCGTCAGACGCGCTACGGATCGCGCCTGGTACAAACCTTTTGCC>Fomitiporia_mediterranea DB=Fomme1_AssemblyScaffolds.fasta ACC=scaffold_3 REGION=3808786-3809055 TAX=Agaricomycotina; Agaricomycetes; Hymenochaetales; Hymenochaetaceae; FomitiporiaAGGCTGTAATGGCCCACTCAAGGGAATGCGAGGTGGTGACTTCCAGCGGCTCCAAATGCCGGTGCGCCGAGTTCGATCCGCCCTCTCGCGAAAACTTGGAGTCCATTTGCCCAGCGCTGCGGAAGCAGTTGCTTACGGCATGGAGTAATGTGACGATGACAGGCCAGGGAAGCAATTCTGCAACCTGCTCGTCCATCCGAGTTAGTCGTATGAGATCGGGGCAGAGAGTCTGTACGCGCTGCGTTTCGTGCCCCTCGCAAACCTTTTATT>Fomitopsis_pinicola DB=Fompi3_AssemblyScaffolds.fasta ACC=scaffold_182 REGION=2685-2957 TAX=Agaricomycotina; Agaricomycetes; Polyporales; FomitopsisTGGCTGTAGCGGCCCTACATCGGGAATGTGAGGTGGTCTAACCAATCAACGGGCTCATTCGTCTGTGCGCTGGTTCAAGGCCGTCCCCTCCAGAAAACTTGGTTGCTTTGGCTGTCCACCGCGGAAGCGGAGGCACGGCCTGGCGTAAACTGTCCTTGACTGGCCAGGGAAGCAATTCCGCAACCTGCAGGACGCGCCGAGTTAGTCTTAGGAGACAAGGACGTGCCGTCTGTTCGCGTAGATGATCCGTGCCCTTCACAAACCCTTTTGCTT>Galerina_marginata DB=Galma1_AssemblyScaffolds.fasta ACC=scaffold_4 REGION=1417449-1417715 TAX=Agaricomycotina; Agaricomycetes; Agaricomycetidae; Agaricales; Strophariaceae; GalerinaCGGTTGTAACGGCCGCAAACCGGGAATGCGGGTGTCCCCTTTCAGCGAATTAAGCTATCAGCGCGCTGTTTCAAGGCTAGGCCCTCCAGAAAACTTGGTTTCCACAGTTCCTCCCACCTTAAGGTGAGGATTGGGCTTGGAATAATCTGTCCTTGTCAGGCCAGGGAAGCAATTCCGCAACCTGCAGGACTCGCCGAGTTAGTCTTAGGAGATCAGACTAGACCGTCAGTTCGCGCAGTGGCTCGCGGTATCCGCAAACCTTTCTTT>Phanerochaete_carnosa DB=Phaca1_AssemblyScaffolds.fasta ACC=scaffold_5 REGION=3346152-3346425 TAX=Agaricomycotina; Agaricomycetes; Corticiales; Corticiaceae; PhanerochaeteTGGCTGTAACGGCCCTAAACCGGGAATGTGAGGTGGTCTAACGCTCCAACGGATTTCCCCGTCTGTGCGCTGGTTCAAGACCGTCCCCTCCAGAAAACTTGGTGCCCCGTTTACCGCCCGCGGCGTAAGCCGTGGATTTGGTTCGGGAGAGTCTGTCCTTGACTGGCCAGGGAAGCAATTCCGCAACCTGCAGGACACACCGAGTTAGTCGTCGGAGACAAGGACGAGTCGTCTGTTCGCGCGGATGGGCCGTGCCCCTTGCAAACCTTCTTTT>Phanerochaete_chrysosporium DB=Phchr2_AssemblyScaffolds.fasta ACC=scaffold_1 REGION=2943014-2943286 TAX=Agaricomycotina; Agaricomycetes; Corticiales; Corticiaceae; PhanerochaeteTGGCTGTAGCGGCCCTAAACCGGGAATGTGAGGTGGTCTAACGCTCCAACGGGTTCACTCGTCTGTGCGCTGGTTCAAGACCGTCCCCTCCAGAAAACTTGGTGCCCCGTTTACCGCCCGCAGCGTAAGCCGTGGGCCTGGTTCGGGAGAATCCGTCCTTGACTGGCCAGGGAAGCAATTCCGCAACCTGCAGGACGCGCCGAGTTAGTCGTCGGAGACAAGGACGAGTCGTCTGTCCGCGCAGATGGGCCGTGCCCCTCGCAAACCTTTTTT>Ganoderma_lucidum DB=GCA_000262775.1_GanLuc1.0_genomic.fna ACC=JH660704.1 REGION=194387-194658 TAX=Agaricomycotina; Agaricomycetes; Polyporales; Ganodermataceae; GanodermaTGGCTGTAATGGCCCTAAACCGGGAATGTGAGGTGGTCTAAAGCTCCAACGGATTTAGTCGTCTGTGCGCTGGTTCAAGACCGTCCCCTCCAGAAAACTTGGTTACCTCGAGTCACCCGTCTCGTAAGAAATGGGTTGACCTGGTATAATATGTCCTTGACTGGCCAGGGAAGCAATTCCGCAACCTGCAGGACGTGCCGAGTTAGTCTTAGGAGATCAGGACGAGTCGTCTGTTCGCGCGGATGGCCCGTGCCCTTCACAAACCCTTACTT>Trametes_versicolor DB=Trave1_AssemblyScaffolds.fasta ACC=scaffold_4 REGION=2698174-2698445 TAX=Agaricomycotina; Agaricomycetes; Polyporales; TrametesTGGCTGTAACGGCCCTAAACCGGGAATGTGAGGTGGTCTAACGATCCAACGGGCTCAGTCGTCTGTGCGCTGGTTCAAGACCGTCCCCTCCAGAAAACTTGGTTACCTCGCACCGCCCGCTCCGTAAGGAGTGGGTTGGGCTGGTATAATACGACCGTGACTGGCCAGGGAAGCAATTCCGCAACCTGCCGGACGTGCCGAGTTAGTCTTAGGAGATCAGGACGAGTCGTCTGTTCGCGCAAATGACCCGTGCCCTTCACAAACCCTTCCTT>Heterobasidion_irregulare DB=Heterobasidion_annosum.AssembledScaffolds.fasta ACC=scaffold_02 REGION=376925-377190 TAX=Agaricomycotina; Agaricomycetes; Russulales; Bondarzewiaceae; Heterobasidion; Heterobasidion annosum species complexTGGCTGTAACGGCCCAAGCCGGGATGTGGAGCGGTGTTATCCAACGGACTCACTCTTGCGTGCGCAAGTTCAAGACTGTCCCCTCCAGAAAACTTTGGTGTCCATCCTTTCCCGCGGCGCAAGCCGTGGCTGAACATGGAATAATTGGACCTTGTCTGGCCAGGGAAGCAATTCCGCAGCCGGCAGGACCCGCCGAGTTAGTCGTCGGAGATCAGGGCATGTCGTCTGCCGCGCGCGAGGGCCGTGCCCTTCACAGACCTTTTATT>Gloeophyllum_trabeum DB=Glotr1_1_AssemblyScaffolds.fasta ACC=scaffold_00003 REGION=3039321-3039592 TAX=Agaricomycotina; Agaricomycetes; Gloeophyllales; Gloeophyllaceae; GloeophyllumCGGCTGTAGCGGCCCAAACCGGGAATGTGAGGAGGTCTGAAGTCACAACGGGTTTCCCCGCTCGTGCGCCGAGTTCAAGACCGTCCCCTCCAGAAAACTTGGTGACCATCACTCGCCGCGGCGGAAGCCGTGGCTCAGTATGGAATAATCGGTCCATGACAGGCCAGGGAAGCAATTCTGCAACCTGCTGGACCCGCCGAGTTAGTCTTAGGAGACCAGGACGAGTCGTCTGTCGTGCGAGTGGGCCGTGCCCCTCACAAACCCTTTTGCTT>Jaapia_argillacea DB=GCA_000697665.1_Jaaar1_genomic.fna ACC=KL197715.1 REGION=695295-695566 TAX=Agaricomycotina; Agaricomycetes; Agaricomycetidae; Jaapiales; Jaapiaceae; JaapiaCGGCTGTAGCGGCCAAATCATGGGAATGTGAGGTGGTCTAACGACTCAACGGAATTACTCGCGCATGCGCTGGTTCAAGACCGTCCCCTCCAGAAAACTTGGTCACCACAACTCTCCGATCCTGAAGGACTGGAAAGAGTTTGGAATAATGGTTCCTTGACGGGCTAGGGAAGCAATTCTGCAACCTTCTGGAGCCGCCGAGTTAGTCGTAGGAGATCAGGACGAGTTGTCTGTTCGCGTGCGTGAGCCGTGCCCTTCATAAACCTTTTGCT>Laccaria_bicolor DB=GCA_000143565.1_V1.0_genomic.fna ACC=DS547096.1 REGION=1247054-1247320 TAX=Agaricomycotina; Agaricomycetes; Agaricomycetidae; Agaricales; Tricholomataceae; LaccariaAGGCTGTAATGGCCCTAAACCGGGAATGCGGGTGGTTTCTTTCATCGGCTAACTCCACGAGTGCGCTGGTTCAAGACTGTCCCCTCCAGAAAACTTGGTTACCACAGACTCTCACCCTGTAAGGTGTGATCTGGTCTTGGAATAATCGGTCCTTGACAGGCCAGGGAAGCAATTCCGCAACCTGCAGGACTCGCCGAGTTAGTCTTCGGAGATCAGGGCATGTCGTCAGTCGCGCAGTGGAGCGTGCCTCCCGCAAACCTTTTTCAT>Leucoagaricus_gongylophorus DB=Leugo1_AssemblyScaffolds.fasta ACC=contig_03497 REGION=1887-2152 TAX=Agaricomycotina; Agaricomycetes; Agaricomycetidae; Agaricales; Agaricaceae; LeucoagaricusCGGTTGTAATGGCCCAACCCGGAATGAGTAGGGTTTCTCCCAACGGTTTCAAGCCATCAGTGCGCTAGTTCAAGACTGTCCCCTCCAGAAAACTTGGTTTCCACAGATCCTCACTCTGTAAGGAGTGACTTGGGCTTGGAATAATTTGCCCTTGACAGGCCAGGGAAGACAATTCCGCAACCTGCAGGACTCGCCGAGTTAGTCTTCGGAGATCAGGGCGTGTCGGCAGTCGTGCTGTGGCTCGTGCCTTGCTCATACCTTTTTGT>Lignosus_rhinocerotis DB=GCA_000743315.1_LigRhi1.0_genomic.fna ACC=AXZM01000930.1 REGION=30652-30923 TAX=Agaricomycotina; Agaricomycetes; Polyporales; Polyporaceae; LignosusTGGCTGTAATGGCTCTAAACCGGGAATGTGAGGTGGTCTAACGTCCCAACGGGTTCAGTCGTCTGTGCGCTGGTTCAAGACCGTCCCCTCCAGAAAACTTGGTTACTTCGAGTCGCCCGCTCCGTAAGGAGTGGATTGGCCTGGTATAATATGTCCTTGACTGGCCAGGGAAGCAATTCCGCAACCTGCAGGACGTGCCGAGTTAGTCTTAGGAGATCAGGACGAGTCGTCTGTTCGCGCAGATGGCCCGTGCCCCTCACAAACCCTTCCTT>Moniliophthora_perniciosa DB=GCA_000183025.1_ASM18302v1_genomic.fna ACC=ABRE01006476.1 REGION=1160-1426 TAX=Agaricomycotina; Agaricomycetes; Agaricomycetidae; Agaricales; Marasmiaceae; mitosporic Marasmiaceae; MoniliophthoraTGGCTGTAGTGGTCCGACTCGGGAATGTGAATGGGTTCATGTCAGCGAATCAAGTCCCGAGTGCGCTGGTTCGAGACTGCCCCCTCTGGAAAACTTGGTGTCCACAACCTCCCGTGCCGCAAGGTATGGCCTCTGGGTTTGGATTAAATTGGACTTGTCAGACTAGGGAAGCAATTCCGCAACCTGCAGTTCGCGCCGAGTTAGTCTCAAGGGATCAGGGTAGTCGTCAGTCGCGCTGAGGCTCGTGCCTTTCACAAACCTTTTTTT>Moniliophthora_roreri DB=GCA_000488995.1_M_roreri_MCA_2997_v1_genomic.fna ACC=AWSO01000135.1 REGION=191341-191604 TAX=Agaricomycotina; Agaricomycetes; Agaricomycetidae; Agaricales; Marasmiaceae; mitosporic Marasmiaceae; MoniliophthoraTGGTTGTAATGGCCCGAACCTGGGAATGTGAGTGGTTCATGTCAGCGAATCAGACCTCGAGTGCGCTGGTTCGAGACTGTCCCCTCCGGAAAACTTGGTGTCCACAGCCTCCCGTGCCGTAAGGTATGGTTCTGGGCTTGGATTAATCAGGACTAGTCAGGCCAGGGAAGCAATTCCGCAACCTGCAGCCCTCTCCGAGTTAGTCTTAGGAGATTAGGACAATCTTCAGTCGCGCTGGGGTCCGTGCCTCTCACAAACCTTTTT>Omphalotus_olearius DB=Ompol1_AssemblyScaffolds.fasta ACC=scaffold_15 REGION=353251-353519 TAX=Agaricomycotina; Agaricomycetes; Agaricomycetidae; Agaricales; Omphalotaceae; OmphalotusTGGCTGTAATGGCCCGAACGTGGGAATGTGAGGTGGTTTACATCAGCGAACAATACGATTCGCGCGCTGGTTCAAGACTGTCCCCTCCAGAAAACTTGGTGTTCATTGCCTCCCGTTCTGTAAAGAATGGTCTGGGCTTGGAATAATATGCACTTGACGGTCCAGGGAAGCAATTCCGCAATCCGCAGTGCTGGCCGAGTTAGTCTTAGGAGATCAGGGTGTGTCGGCAGTCGCGCGGTCTGTACGCGCCTCTCACAAACCTTTTTCTT>Phellinus_noxius DB=GCA_000507345.1_OVT-YTM_97_Newbler_2.8_genomic.fna ACC=AYOR01000052.1 REGION=37047-37319 TAX=Agaricomycotina; Agaricomycetes; Hymenochaetales; Hymenochaetaceae; PhellinusCGGCTGTAATGGCCCTAAATTGGGAATGCGAGATGGTAACTTTCAGTGGGTTACTTGTCAAGCGCGCTGGTTCAAGACTGACCCCTCCAGAAAACTTGGACGCCAAATTGTCTATCGTTGCGTAAGCATTGATTTAGACTTGGAATAGTCTGTCGGTGACAGGCCAGGGAAGCAATTCTGCAACCTGCCCGACAGTCCGAGTTAGTCTTAGGAGATCAGGTCAGTTCGTCTGTCCGCGCAGGCAATCACGCCTCTCGTAAACCTTTTCCAATT>Piriformospora_indica DB=Pirin1_AssemblyScaffolds.fasta ACC=PIRI_contig_0055 REGION=40792-41070 TAX=Agaricomycotina; Agaricomycetes; Sebacinales; Sebacinaceae; PiriformosporaAGGCTGTAATGGCCCTAACTCGGGAATGCGAGCTGTTTCCCTTGGCGGAGTTTCTCATTCGTGCGCTAAGTTCAAAGCTCTGCCCTCCAGAAAATCTTGTAGCGACTTTTCAGCCGCCGCGGCGTAAGCCATGGCGGGCGGTCGCGTAGCTGATGCTTCGACGTTTGTCAATTGGCCAGGGGAGCAATTCCGCAGCCAGGAGTGTCAACCGGATTAGTCTTAGGAGACCAGGAGATGCTTTCAGTCGCGCGGTGGGTCCGTGCAGCTCGCAAACTTTTT>Pleurotus_ostreatus DB=PleosPC15_2_Assembly_scaffolds.fasta ACC=scaffold_01 REGION=4109898-4110168 TAX=Agaricomycotina; Agaricomycetes; Agaricomycetidae; Agaricales; Pleurotaceae; PleurotusTGGCTGTAACGGCCCCAAACCGGGAATGCGAGGTGGTTGCTCCCAGCGGCTAAATCCATCAGCGCGCTGGTTCGAAACCGTCCCCTCCAGAAAACTTTGGTGTTCTCACTCCCCTCGCGTCGTAAGACGCGGCCCGGGCGTTTGAGTAATCCGACCTTGTCAGGCCAGGGAAGCAATTTCGCAACCTGCAGGTCACGCCGAGTTAGTCTTCGGAGATCAGGATGCGTCTACAGAACGCGCAGTGGATCGTGCCCCTCGTAAACCTTTTCTT>Postia_placenta DB=GCA_000006255.1_Postia_placenta_V1.0_genomic.fna ACC=EQ966324.1 REGION=154995-155265 TAX=Agaricomycotina; Agaricomycetes; Polyporales; PostiaTGGCTGTAACGGTCCTATTCCGGGAATGTGAGGTGGTCTAAAGCTCCAACGGGTTCACTCATCTGTGCGCTGGTTCAAGACCGTCCCCTCCAGAAAACTTGGTTACCTTGTCCGACCACCGCGAAAGCGGAGGCTTGGTTCGGTGTAATTTGCCCTTGACTGGCCAGGGAAGTAATTCCGCAACCTGCAGGGCATGCCGAGTTAGTCTTAGGAGATCAGGACGAGTCGTCTGTTCGCGTAGATGGGCCGTGCCTCTCACAAACCTTTCTTT>Punctularia_strigosozonata DB=Punst1_AssemblyScaffolds.fasta ACC=scaffold_8 REGION=1041951-1042215 TAX=Agaricomycotina; Agaricomycetes; Corticiales; Punctulariaceae; PunctulariaTGGCTGTAACGGCCCCAACTCGGGAATGCGAGGTGGTTCTTATCAGCGGTCCAGTTCCGCAGTGCGCTGGTTCAAGACCGTCCCCTCGAGAAAATTTGGTGACCATCGCTTCACGCGGCGTAAGCTGTGAGCAAGCATGGAATAATCTGTCCTTGACTGGCCAGGAAAGCAATTTCGCAACCAGCAGGACTCGCCGAATTAGTCTTCCGAGACCAGGACGTGTCGTCAGTTCGCGCTCGGGCTCGTGCCCTTCGCAGACCTTTTT>Schizophyllum_commune DB=Schco3_AssemblyScaffolds.fasta ACC=scaffold_1 REGION=135715-135975 TAX=Agaricomycotina; Agaricomycetes; Agaricomycetidae; Agaricales; Schizophyllaceae; SchizophyllumATGCTGTAATGGCCTAACGGGAATGCGGCGGGTTAATCACCGGTCAAAGTCTCGAGCGCGCTGTTTCAAGACCGTCCCCTCCAGAAAATTTGGTGTCCACAGTTTCCCGGCGCGCAAGCGCTGGTTCGAACTTGGAATAATCTGCCCTTGTCAGGCCAGGGAAGCAATTCTGCAACCTGCAGGGCCCGCCGAATTAGTCTTCGGAGACCAGGACGTGTCGTCAGACGCGCTGAGGCTCGGGCCTGCTGCAAACTTTTTGTG>Serpula_lacrymans DB=Serpula_lacrymans_S7_3_v2.unmasked.fasta ACC=scaffold_11 REGION=1336474-1336741 TAX=Agaricomycotina; Agaricomycetes; Agaricomycetidae; Boletales; Coniophorineae; Serpulaceae; SerpulaTGGCTGTAATGGCCCTAAACCGGGAATGTGAGGTGGTTCTTTACAGCGGTTAAATCCACGAGTGCGCTGGTTCGAAACTGTCCCCTCCGGAAAACTTGGTTGCCACAGTTTGTCGCGTCGTAAGGCGTGAAGTGTAACTTGGAATAATCGGCTCTTGTCAGGCCAGGGAAGCAATTCCGCAACCTGCAGAGCTCGCCGAGTTAGTCTTAGGAGATCAGGACCGTTGGCAGTCGCGCAGTGGGTCGCGCTTCTCACAAACCTTTTGCTT>Stereum_hirsutum DB=Stehi1_AssemblyScaffolds.fasta ACC=scaffold_15 REGION=518170-518449 TAX=Agaricomycotina; Agaricomycetes; Russulales; Stereaceae; StereumTGGCTGTAGCGGCCCAAAGCCGGGAATGCGAGGTGGTGTCAGCACTCAACGGAATCACTTTCACGTGCGCCGAGTTCAAGACCGTCCCCTCCAGAAAACTTTGGCGTCCACACCTCTCTCGCCGCGGAAGCGCGTGGGAAGAGTTGGAGTAATCGGACCTTGATTGGCCAGGGAAGCAATTCCGCAGCCGGCAGCTTGTCCCGCCGAGTTCGTCGTAGGAGATCAGGGCGATGTCGTCTGTTCGCGCGTGGGGGCCGTGCCTCTCGCAAACCTTTTGCTT>Wolfiporia_cocos DB=GCA_000344635.1_Wolco1_genomic.fna ACC=KB467942.1 REGION=2237382-2237653 TAX=Agaricomycotina; Agaricomycetes; Polyporales; WolfiporiaTGGCTGTAATGGCCCTATTCCGGGAATGTGAGGTGGTCTAAAGTTACAACGGGTTTACTCGTCTGTGCGCTGGTTCAAGACCGTCCCCTCCAGAAAACTTGGTCACCTTGGCTGTCCACCGCGGAAGCGGAGGCACGGCCCGGTATAATCCGTCCTTGACTGGTCAGGGAAGCAATTCCGCAACCTGCAGGACGCGCCGAGTTAGTCTTAGGAGACAAGGACGAGTCGTCTGTTCGCGTAGATGGGCCGTGCCCTTCACAAACCTTTTTATT>Volvariella_volvacea DB=GCA_000349905.1_VVO_genomic.fna ACC=KB722738.1 REGION=315184-315449 TAX=Agaricomycotina; Agaricomycetes; Agaricomycetidae; Agaricales; Pluteaceae; VolvariellaCGGTTGTAACGGCCGTCACTCGGGAATGCGATGGGTTCTTCTCACCGGCTAACACCGTCAGTGCGCTGGTTCAAGACTGTCCCCTCCAGAAAACTTGGTTTCCACAATCTCTCGCCCTGTAAAGGGTGACCTCGGATTTGGAATAATTTGTCCTTGACATGCCAGGGAAGCAATTCCGCAACATGCAGGACTCGCCGAGTTAGTCTTCGGAGATCAGGGCATTGTCGTCAGTCGCGCAGCGGTGCGGGCTTGTCGCAAACCTTTTT>Mixia_osmundae DB=GCA_000708205.1_Mixia_osmundae_v1.0_genomic.fna ACC=KL411554.1 REGION=227681-227940 TAX=Pucciniomycotina; Mixiomycetes; Mixiales; Mixiaceae; MixiaCGGCTGTAATGGCCCAACCTTTGGAATGTCCACGTCAGAATTCGTGGAATCACACCAAATTTGCGCACTTCTTTCTTTCGGGCGTCCTAGCTTGAGCTCTCGGACAAGATCGCAAGCCCCTTCTGCAGAGATGGGTGCTTCGTCTTGATGACTAGATTTTCAATCGAGTCCGGTAACGGAGCAGATCTGGAAATCTTTCCGGGAACTCTTGATGGCAGGATCGCGCTCGCTGGGCTTCGGAAGTGGATTTGCCTCAAGTC>Rhodotorula_glutinis DB=GCA_000222205.2_R.glutinis2_genomic.fna ACC=AEVR02000018.1 REGION=201413-201708 TAX=Pucciniomycotina; Microbotryomycetes; Sporidiobolales; mitosporic Sporidiobolales; RhodotorulaTCGCTGTAATGGCTCTTTTGTGGGAATTGCACGACGAATACACAAGGATTACACGGCCGTTGGCTCGCCAAACTACCAAAAGCTCGATGTCTGCCCGTGAACACCCGGTCAAGTCGACCCGCCTGCGCAAGTGGACGGGGTATCAGACTCGTTGAATGGGCGTGCTATTCAGTCGGGTTAACCGAACAGGATAGCGCGCTCAGCCGGGGGTTCTTGTCGGGCAGAGGACGTCGAGCTGGTGAGAGACTTCTCTTCAAGCAGCCGCCGCGCCGCCTCGTCGCGCGAAACCCACTTTT>Dacryopinax_sp DB=Dacsp1_AssemblyScaffolds.fasta ACC=scaffold_5 REGION=318755-319026 TAX=Agaricomycotina; Dacrymycetes; Dacrymycetales; DacrymycetaceaeCGGCTGTAATGGCCGCAATGAGGGAATGCGGTGGGTAATCATGCACAACGGATTACCGAAAGTGCGCTGGATCGAGCCCCGCCCCCCACGACACTTTGGGCCTTTATGGCGCAGGACGCAAGTCCCGTGGCCAATGAAAGGATAATGGATGGTGGTATTCCAGCCAGGGAAGAGATTCCGCAACTGGACGCCTCCTCCGAGGTCGTCTTCTGGGACACGGTGGGTAGCTGTAGTTCGCGCTGGAGGCCCGTGCTTGCCGTAAACCTTTTGCT>Rhizoctonia_solani DB=GCA_000334115.1_Rhisol_AG1IA_1.0_genomic.fna ACC=KB317700.1 REGION=914160-914424 TAX=Agaricomycotina; Agaricomycetes; Cantharellales; Ceratobasidiaceae; ThanatephorusACGCTGTAATGGCGCAATCGGGAATGCACAGTGGTCCATTTCAACGGATTACTCAGATGTGCGCTGGTTCGAAACCCTCCCCTCGCAGACGTCTTGGTCTGAATTTCCACCGCACGGCAACGCATGGTCGGACTTTCGGTAATCATTCCGCGTTCTTGTCAGGGAGTCAACTCCGCAGCACCGCAGGGTGACCGAGGCTGTCCACCGAGACAAGGAGGTGTTTCCAGACGCGCTACTGGGCCGTGCCCGGTGCAAACCTTTTGTC>Wallemia_sebi DB=GCA_000263375.1_Wallemia_sebi_v1.0_genomic.fna ACC=JH668231.1 REGION=272006-272299 TAX=Basidiomycota incertae sedis; Wallemiomycetes; Wallemiales; Wallemiales incertae sedis; WallemiaCAGCTGTAATGGCATCACGGAAGTTGATGAATAAGCTAAAGCTAAGGAGGATTAGCGATGAGCGCTGTCACGCACATCTCCGTTGAAGTCCTGGGTTCGAGGAGCGATTACTGAGCAATTAGTATTTTCGTTTCCTCGATTAAGAATCGTCTACACACAATTCGTAGTCAGGCAACTGCACACGATTAGACGATTTACCTGGATATTCATTAATGGATCGGATGTGAGAACGTACAGACGCGCAACGAGACTCCTGTTCTTCAACGACTATTCTTATTATCATTTTCTTTTTTT>Wallemia_ichthyophaga DB=GCA_000400465.1_Wallemia_ichthyophaga_version_1.0_genomic.fna ACC=KE007245.1 REGION=162696-162968 TAX=Basidiomycota incertae sedis; Wallemiomycetes; Wallemiales; Wallemiales incertae sedis; WallemiaCAGCTGTAATGGCTCAACAGGAAGTTGGTGAGATAAAACTTAAGAGGGATTAGCAATGAGCGCTGGTACGACCATCTCCATTGAAGTCGCGGGTTCGAGTTGCGACCAGGTAGTAATATCTGTCCTCGTTCACTTGAGTAAAGGCGAATCGTGTACACCCACAGTCAGGCAACTGCACATGGGCGGTTCGTTTCCCGCGATATTCTAAAGTGGATTTGATGGTCTAATGGCTAGACGCGCTTTGAGACTTCTGTCTTCCAACGACTTTTGTCT>Heterobasidion_annosum DB=GCA_000633895.1_Ha2.0_genomic.fna ACC=AOSL01000163.1 REGION=30811-31076 TAX=Agaricomycotina; Agaricomycetes; Russulales; Bondarzewiaceae; Heterobasidion; Heterobasidion annosum species complexCGGCTGTAACGGCCCAAGCCGGGATATGGAGCGGTGTTATCCAACGGACTCACCCTTATGTGCGCAAGTTCAAGACTGTCCCCTCCAGAAAACTTTGGTGTCCATCCTTTCCCGCGGCGCAAGCCGTGGCTAAACATGGAATAATTGGACCTTGTCTGGCCAGGGAAGCAATTCCGCAGCCGGCAGGACCCGCCGAGTTAGTCGTCGGAGACCAGGGCATGTCGTCTGCCGCGTGCGAGGGCCGTGCCCTTCATAGACCTTTTATT>Rhodosporidium_toruloides DB=GCA_000320785.2_RHOziaDV1.0_genomic.fna ACC=KB722667.1 REGION=87507-87802 TAX=Pucciniomycotina; Microbotryomycetes; Sporidiobolales; RhodosporidiumTCGCTGTAATGGCTCTTTTGTGGGAATTGCGCGACGAATACACTAGGATTACACGGTCGGTGGCTCGCCAGATTACCAAAGGCTTGGTGTCTGCTCGTGAACACCCGGTCGAGTCGACCCACCTGCGCAAGTGGATGGGGCAATAGACTCGTTGAATGGGCGTGCTATTCAGTTAGGTTAACTGAACAAGATAGCGCGTCCAGCCGGGGGTTCTTGTCGGGCAGAGGACGCCGAGCTGGTGAGAGACTTCTCTTCAAGCAGCCGCCGCGCCGCCTCGTCGCGCGAAACCCACTTTT>Puccinia_graminis DB=Puccinia_graminis.unmasked.fasta ACC=supercontig_2_13 REGION=896336-896690 TAX=Pucciniomycotina; Pucciniomycetes; Pucciniales; Pucciniaceae; PucciniaAAGCTGTAATGGCACAACCGGGGGATAGGCCAGTGTGAAGTGACAAACTGAACCAGGTATATCGAGCGCACTGCGTCCGGCGAGGTGTCCGAGACGAGAAGGCCAGGTCCTGGGTTCGAAGCCCGCTCTCCCGCAGGGGTCAGTGGGCTGCCCGGCTAAGTTGATCAGTGGCCGGGGACTTGTGCCTCGTGTCCAATCGTCTGAAAGGATGATTGTTGCGGTAATGGGTCACCCCAGCATCCGCGGGTGGTCAGCCTGGTTCCTTCTCTTCGTCCGGGTCGCTCCTTGTCTCAGTCTTCATCTCTGATGTGTAAGACGCTTGAGTATCTCAGTACCTTGTCTAAACCCTATCTTT>Puccinia_striiformis DB=GCA_000474995.1_Cy321.0_genomic.fna ACC=KI517231.1 REGION=95830-96185 TAX=Pucciniomycotina; Pucciniomycetes; Pucciniales; Pucciniaceae; PucciniaAAGCTGTAATGGCACAACCGGGGGATAGGCCTGTGTAACCTAAAACTGATCCAGGGGCATCGAGCGCACTGAAGCCTCATAGACGGGGTGTCCGTGCCGAGATGACCAGGTCACGGGTTCGAAGCGTGCTCTCCCGCAAGGAGGAGCGTGCTACCTGACTAAGTCGGCCAGTGGCCGGGGCCTCGTGCCCCGCGTCCGATCTTCTCTCGAGGATTGTCGTGGTATTGGGGCAACCCAGCATCCACGGGTGGTCAGCCTGGTCTCATCTGTTCGGCCGGTTCGCAGCTCGTCTCAGTCTTTCCATCGAATGTTTAAGACGCTTGGACACCTCAGTACCTGGCCTAAACCCCATTATT>Heterogastridium_pycnidioideum DB=Hetpy1_AssemblyScaffolds.fasta ACC=scaffold_8 REGION=161580-161862 TAX=Pucciniomycotina; Microbotryomycetes; Heterogastridiales; Heterogastridiaceae; HeterogastridiumTCGCTGTAATGGCCTGTTGGGATTTGTTGGGGCACTTAGTGAATCCGCGTTTGCTGATCGCCACAAGAACGCTCGTCTTTCAATGAACTTTGGGGCTCTTTGTCTGACTGGCGCAAGTCTGTTGGTAGCAGGGAGATAATTAGGGGTGTTATTCTGTTAGACTGGCTTCACAGGGCAAACAGGCACTCCTACCCGGAAGTTCTTGTTTGGAAGATCGCGGGCGTTTGGTGAGCTCATCTCTTCAAGATTGGCTTGCGTACACCCCCAGCAAAGACCAACTTTT>Tilletiaria_anomala DB=GCA_000711695.1_Tilletiaria_anomala_UBC_951_v1.0_genomic.fna ACC=JMSN01000031.1 REGION=49461-49762 TAX=Ustilaginomycotina; Exobasidiomycetes; Georgefischeriales; Tilletiariaceae; TilletiariaTCGCTGTCATGGCACTCTTTCAGGGATGCGGTCCCTGTGGCGCGCCCCGCACTAGTTGGGCTGCCGTGCAAGGACTGAGGTTGCACCGAACGGCGCGTCTGCAAAGCGTGTTTAGTTCGAGCTGCATAAATGGCTTGGTATCAATTTAGGTCTAAATCCTATCTTTGGAGGAGGTGGTCACCCGCGCACTCCACCACGGCCGACCTCAATCGAACGTCTCGTGCGGATCACAGTTTCCATGCTTGGGGACACTTGTCTGAGAGCGCGCTACAGTCTCACAAGATCGCAAACTTCCTTTCTTT>Melampsora_laricis-populina DB=Melampsora_laricis_populina.AssembledScaffolds.fasta ACC=scaffold_102 REGION=115890-116271 TAX=Pucciniomycotina; Pucciniomycetes; Pucciniales; MelampsoraceaeAAGCTGTAATGGCAAAATCGGAGGAATCTCTCGCACTTCCCAATAAAACTGGGTCAAACCATCCCAAGCGCACTGAGTTCGAATCTCACATGATGTCCATCACGAACCAAGGCAATCCAGGTACCTTTGTCCACTTCCTCCCGCAAGGGTAGTTGTAACATAGGTTAAAGGCAGTCATTGATCATCCTTGTGGTGGTCAGAACAGTATTGGGGTAACCCGGCATCTGTAAATGGCTCACCTGGCTCGGTTGATCGGGTCGATTTGTCTCTCCATGTTTAGGAGTTTGTTGTTCGTCTCGGATCGCATCGTGTGGGGAAATGAGTCAGTCTGAATATGATAAGCCGCATGGGTTGGTCCAGGGTGCTTGAGAAACTTCCACTT>Ustilago_maydis DB=GCA_000328475.1_ASM32847v1_genomic.fna ACC=AACP01000131.1 REGION=47908-48230 TAX=Ustilaginomycotina; Ustilaginomycetes; Ustilaginales; Ustilaginaceae; UstilagoTACCTGTTACGGGTGTAACCTTGGAATGAAGTCTCAGCCGAAGCGCGCCCCATAAGTGTTAGCCTTTCCGTCGGATGGTTGAGGATTTGCTGTTTCATGCACACGTCCAGCAGTGGATGTCTGCTCAGCAAGTAAGCCAGTGTGGTGACCCATTGAATTGGGAGTCAATAGCCTCCGGGCTCGAAGGAGTCGGTCAACCGAGCACTTCACACACTGATCCTCAGCTGCACGTATCCGTCGGGCTACAAGGTGGATAGGGAGCATTCACTTCAATTGGAGTTGCTTGAAAGCTGCGCATCTGGGATCACTTCAAACCTTTTTCT>Pseudozyma_antarctica DB=GCA_000747765.1_ASM74776v1_genomic.fna ACC=DF830078.1 REGION=575201-575524 TAX=Ustilaginomycotina; Ustilaginomycetes; Ustilaginales; Ustilaginaceae; mitosporic Ustilaginaceae; PseudozymaCACCTGTAATGGGTGCACCTCCGGAATGAGGTCTCTGCCAAAGCGCGCCCCACAAATGTTAGCCTTTCCGTCGGATGGTTGAGGATCTGCCGTTCAGCGCGCGTCCAGCAGTGGATGCCCGCTCGGCAAGTAAGCCAGTGTGGCGACCCATTGAGTTGGGGATTAATAGCCTTCGGGCTCGAGGGAGTCGGTCGCCCGAGCACCTCACACACTGAACCTCAGCTGCACGTATCCGACGGGGTCCAAGGCGGATAGGGAGCATCTACTTCCTCGTTTGGAGTTGCTTGAAAGCTGCGCATCTGGGATCGCCTCAAACCTTCTTTT>Malassezia_sympodialis DB=GCA_000349305.2_ASM34930v2_genomic.fna ACC=HE999571.1 REGION=18940-19230 TAX=Ustilaginomycotina; Exobasidiomycetes; Malasseziales; Malasseziaceae; MalasseziaTACCTGTCATGGGTGTACCGTTGGGATGAGTCTACCTGAAGCGCGCCCCGCATAATGTTAACCGTTCCTTTGGATGATTGAGGTGGGCTAGTTTCATGCGCGTGCTTCACAGCATGCCCAGTCTAGCTGTAAATGGGAGGGTTCAAGGAATTGCATGCAATTTGTTGGATCAGTCGGTTCTCCGAGCACTCCGCCCTACCGACCTCAGTCCTACGTATCCATGGGGTTCTCGGCAAGCAGGGAGCACATGGCTTGAAAGCGCGCTCAGGCCTTATGAAGATTCAAACTTTT>Naiadella_fluitans DB=Naifl1_AssemblyScaffolds.fasta ACC=scaffold_2 REGION=585201-585494 TAX=Pucciniomycotina; Classiculomycetes; ClassiculalesAGACTGTAATGGTCCAACCGGGAATTCGTCTCTGCACCAGCCATCGGATCAAACCCCGTTCAGAGCGCTCGTTATCCCAGTCCGCCCTGTATGAGTGTCAAGTCCTCGTTCTGTTCGACCCTGCGCAAGCATCGTCGTCACCGAGTTGATACGGGCGCGTGCACCCAGGCCGGCAACGGATCATGGTGGGCGTGCGAGCTTGAGGCTCTTGAAGTACGGGATTACGGACGGTAGAGTGCGCCATTTCAGCTTGAAGCGCTGAGCAGGGCCGACGGAGACGTCAAACCTTTTTGT>Sporisorium_reilianum DB=Spore1_AssemblyScaffolds.fasta ACC=chromosome_10 REGION=585187-585507 TAX=Ustilaginomycotina; Ustilaginomycetes; Ustilaginales; Ustilaginaceae; SporisoriumCACCTGTCATGGGTGTAACCTTGGAATGAGGTCTCTGCCGAAGCGCGCCCCACTAGTGTTAGCCTTTCCGTCGGATGGTTGAGGAGCTGCTGTCCTGCACACGTCCAGCAGTGGATGTCTGCTCAGCAAGTAAGCCAGTGTGGCGGCCCATTGAATTGGGAGTCAACAGCCTTCGGGCTCGAAGGAGTCGGTCGCCCGAGCACATCACACACTGATCCTCAGCCGCACGTATCCGATGGGTTGCAAGGCGGATAGGGAGCATTCACTTCGTATGGAGTTGCTTGAAAGCTGCGCATCTGGGATCACCTCAAACCTTTTTTT>Microbotryum_violaceum DB=GCA_000166175.1_M_violaceum_V1_genomic.fna ACC=GL541759.1 REGION=49907-50278 TAX=Pucciniomycotina; Microbotryomycetes; Microbotryales; MicrobotryaceaeCGGCTGTAATGGTCGTCCCTCAGGGAACGTCGCATTGATCCATTTGGGACTCACTATCCGTTGCTCGCCAATCCTAATCCTTGGATTGGGGATTAAAAGCCTTTAGCGATCTAGTCGTGAACTCGAGGTTCCTTGAAGCCCGTCTGCGCAAGCGGGCAGGGTGATTCAGGGTTGAATTTGACGCGTATCATTGGCCCTCCAATGAACTTTCTTTAGTCCCGCCAGACCGGTTAACGGGGCAGGCGGTGCGCGCTCAGCCTTAGGTTCTTCTATCGACAAGAGCATCGTTTGGCTGGTGGTCGCTTCCTTGACTTTGTCGAGGGCTGCCTTGAAGATGCGGCGTGATGGCCCCGGTGCGATGAACTCTTTTTT>Agaricostilbum_hyphaenes DB=Agahy1_AssemblyScaffolds.fasta ACC=scaffold_5 REGION=601157-601468 TAX=Pucciniomycotina; Agaricostilbomycetes; Agaricostilbales; Agaricostilbaceae; AgaricostilbumGCACTGTAATGGTGCTAACCCAAGGAATGCGATTCTGTATCTCAAGCGGAATCAGAAGTTAGCGCGCTCCAGCTGGTCTCACTTAACATTGGTCCATTCGAATTCACGTCGGAATTGAGCTAGTAGTGAAAGCTACTGTGTTCGAATCCGTTAAATTCCAGGATGTCAAATTGTGTTAGGCGGGTTTCACCGAGCAGCACCATCCTAGAGGCGTGTCTTCATTACAATTGGATAGCCAATTGGGGCCATAAAAGCTGGACTCTATAGAGATAAGGCGCGCAAGCTTCCCGCCAGGGTTGCAAACTTTTTGTT>Amanita_muscaria DB=Amamu1_AssemblyScaffolds.fasta ACC=scaffold_7 REGION=234602-234867 TAX=Agaricomycotina; Agaricomycetes; Agaricomycetidae; Agaricales; Amanitaceae; AmanitaCGGTTGTAATGGCCGCAATTCGGGAATGCGTGGGATGCATCTCATCGGTTAAACCCGCAAGTGCGCTGGTTCAAAACTGTCCCCTCCAGAAAACTTGGTTTCCACAGTCCCTTGCTCCGTAAGGGGTAATTCGGACTTGGAATAATCTGTCCTTGACTGGCCAGGGAAGCAATTCCGCAACCTGCAGGACTAGCCGAGTTAGTCTTCGGAGATCGGGACAGTTCGTCAGACGCGCAGCGGTTCGAGTCCCGCGTAAACCTTTTTGT>Amanita_thiersii DB=Amath1_AssemblyScaffolds.fasta ACC=scaffold_5 REGION=322363-322628 TAX=Agaricomycotina; Agaricomycetes; Agaricomycetidae; Agaricales; Amanitaceae; AmanitaCGGTTGTGACGGCCGCAATTCGGGAATGCGAGGGATTCTTCTCATCGGCTATTTCCGCAAGTGCGCTGGTTCAAGACTGTCCCCTCCAGAAAACTTGGTTTCCTCAGTTCCTCGCTCCGTAAGGGGTGATCCGAATTAGGAATAATCTGTCCTTGACTGTCCAGGGAAGCAATTCCGCAACCAGCAGGACTCGCCGAGTTAGTCTTCGGAGATCAGGGCATGTCGTCAGTCGCGCAGTGGAACGTGTCCCTCGTAAACCTTTTGTT>Antrodia_sinuosa_1 DB=Antsi1_AssemblyScaffolds.fasta ACC=scaffold_2 REGION=299151-299421 TAX=Agaricomycotina; Agaricomycetes; Polyporales; AntrodiaTGGCTGTAACGGCCCTAATCCGGGAATGTGAGGTGGTCTAAACTTTCAACGGGCTCACTCGTTTGTGCGCTGGTTCAAGACCGTCCCCTCCAGAAAACTTGGTCACTTCAGCTTTCCGCAGCGCAAGCTGTGGTTTGGCTTGGTGTAATTCGTCCTAGACTTGCCAGGGAAGCAATTCCGCAACTTGCAGGACGCGCCGAGTTAGTCTTAGGAGATAAGGACGAGTCGTCTGTTCGCGCAGATGGGCCGTGCCCTTCACAAACCTTTCCTT>Antrodia_sinuosa_2 DB=Antsi1_AssemblyScaffolds.fasta ACC=scaffold_150 REGION=37907-38170 TAX=Agaricomycotina; Agaricomycetes; Polyporales; AntrodiaTGGCTGTAACGGCCCTAAACTGGGAATGCGAGGTGGTTAGTTTCAGCGTGTAAATCCATTAGTGCGCTGGTTCAGGACTGTCCCCTCCAGATAACTTGGTGTCTGCGCGAATTTCCTTCCGGAAGGTTGGATCATTCCCAGAGTAAACCGACGTTCGTGAGCCAGGGAAGCAATTCCGCAACTCAGTAGTTGGGCCGAGTCAGTCTTAGGAGATAAGGATGGCCGTCAGTTCGCGCAGTGGATCGTGCCCTTCGTAAACCTTTT>Armillaria_mellea_1 DB=Armme1_AssemblyScaffolds.fasta ACC=NODE_54521 REGION=14-269 TAX=Agaricomycotina; Agaricomycetes; Agaricomycetidae; Agaricales; Physalacriaceae; ArmillariaCCTAAACCGGGAATGTGAGTGGTTTACGTCAGTGATTCAGTCCGCTTGTGCGCTGGTTCAAGACTGTCCCCTCCAGAAAACTTGGCTTCCACAGCCTTCCGCTCTGCAAAGAGTGGTCTGGGTTTGGAATAATTTGTCCTTGATTGGCTAGGGAAGCAATTCCGCAACCTGCAGGACTGGCCGAGTTAGTCGCAGGAGATCAGGGTAAGTCGTCAGTTCGCGCTACGGACCGCGCTTCTCACAAACCTTTTTGCTT>Armillaria_mellea_2 DB=Armme1_AssemblyScaffolds.fasta ACC=NODE_73269 REGION=8626-8908 TAX=Agaricomycotina; Agaricomycetes; Agaricomycetidae; Agaricales; Physalacriaceae; ArmillariaTGGCTGTAATGGTCCTAAACCGGGAATGTGAGTGGTTTACGTCAGTGATTCAGTCCGCTTGTGCGCTGGTTCAAGACTGTCCCCTCCAGAAAACTTGGCTTCCACAGCCTTCCGCCCTGCAAAGAGTGGTCTGGGCTTGGAATAATTTGTCCTTGATTGGCTAGGGAAGCAATTCCGCAATAGGGCTGCGCACGAAACCTCCAGGGGGGTACTATACCGTTTTTTACCGGGACCCCCAAGAGGGGGGGTTTCGCTACGGTTTAGGGCTCCAACCGTTTTCATT>Artolenzites_elegans DB=Artel1_AssemblyScaffolds.fasta ACC=scaffold_20 REGION=380047-380318 TAX=Agaricomycotina; Agaricomycetes; Agaricomycetes incertae sedis; Polyporales; Coriolaceae; TrametesTGGCTATAATGGCCCTAAGCCGGGAATGTGAGGTGGTCTAACGATCCAACGGGCTCAGTCGTTTGTGCGCTGGTTCAAGACCGTCCCCTCCAGAAAACTTGGTTACTTCGCGCCGCCCGCTCCGTAAGGTCCGGGTTGGGCTGGTATAATACGACCTTGACTGGCCAGGGAAGCAATTCCGCAACCTGCAGGTCGTGCCGAGTTAGTCTTAGGAGATCAGGACGAGTCCCCTGTTCGCGCAGATGACCCGTGCCTCTCACAAACCCTTCCTT>Auricularia_subglabra DB=Aurde3_1_AssemblyScaffolds.fasta ACC=scaffold_188 REGION=37762-38033 TAX=Agaricomycotina; Agaricomycetes; Auriculariales; Auriculariaceae; AuriculariaCGGCTGTAATGGCCCACACCCAGGGAATGCATGGCGGTGTCACTTAACGGACACCTCAGCCGAGCGCTGGTTCAAGGCCCTCCCCTCCAGGCAATTTGGTCCTGCACTACCCATCGTCTGGCAGCAGACAATAGGGTTGCAGATAATTGAGGCTTTACACTCAGGCCAGGGAAGTAATTCCGCAGCCAAAGTCTCAGCCGAACCTGTCTAAGGAGACCAGGAGGCGCTGGCAGTTCGCATGGCTGAGCCGTGCCACATGCAAACCTTTTTCA>Bjerkandera_adusta DB=Bjead1_1_AssemblyScaffolds.fasta ACC=scaffold_9 REGION=569929-570202 TAX=Agaricomycotina; Agaricomycetes; Polyporales; Meruliaceae; BjerkanderaTGGCTGTAGCGGCCCTAAACCGGGAATGTGAGGTGGTCTAACGCATCAACGGGTCCACTCGTCTGTGCGCTGGTTCAAGACCGTCCCCTCCAGAAAACTTGGTGTCCCGTTTAACCTCCCGCGGCGTAGGCCGTGGATTGGTGCGGGATAATACGTCCTTGACTGGCCAGGGAAGCAATTCCGCAACCTGCAGGACGCGCCGAGTTAGTCGTCGGAGACCAGGACGAGTCGTCTGATCGCGCAGACGGGCCGTGCGTCTCGCAAACCTTTTGTT>Boletus_edulis DB=Boled1_AssemblyScaffolds.fasta ACC=scaffold_151 REGION=22356-22622 TAX=Agaricomycotina; Agaricomycetes; Agaricomycetidae; Boletales; Boletineae; Boletaceae; BoletusCGGCTGTAATGGCCGTAACCCTGGGAATGCGAGATGGTTCTACACAGCGATATCACTCACGAGTGCGCTAGTTCAAGACTGTCCCCTCCAGAAAATTTGGTCTCCACAGTTTCTCGCGCCGTAAGGTGTGACCAGACTTGGAATAATTGATCCATGATTGGCCAGGGAAGCAATTCCGCATCCGACTGGATCTGCCGAATTAGTCACAGGAGATCAGGACACGTCGTCAGCCGCGCTGTGGGTCGCGCCGCTCGCAAACCTTTTTCT>Calocera_cornea DB=Calco1_AssemblyScaffolds.fasta ACC=scaffold_23 REGION=180480-180750 TAX=Agaricomycotina; Dacrymycetes; Dacrymycetales; Dacrymycetaceae; CaloceraTGGCTGTCACGGCCCTCTCTAGGGAATGCGGTGGGTTCACCCCCCAGCGGACTCCCCGGATGCGCGCTCGATCGAGCCCCGCCCCTCACGACCCCTTGGTCCATCCCGGCGCAGGACGCAGGTCCCGCACAGCCTCTCGATGGATAATGGCCGTGCCGCGCCAGCCAGGGAAGCAATTCCGCAACTGGCCGCGGCCGCCGAGGGCGTCTTCTGGGACAAGGCGGGTGCTCTAGTCGCGCTTCCGGGCCGCGCTTGCCGCAAACCTTTTGCT>Calocera_viscosa DB=Calvi1_AssemblyScaffolds.fasta ACC=scaffold_1 REGION=754509-754778 TAX=Agaricomycotina; Dacrymycetes; Dacrymycetales; Dacrymycetaceae; CaloceraTGGCTGTAATGGCCCTCCCTCGGGGATGCGGTAGGTTTCACACCTAGCGGGTCTGCGAGAGTGCGCTCGATCGAGCCCCGCCCCCCACGACAGCTTGGTCCATTTCGGCGCAGGAAGCAATTCCCGCACTGCCACCGATGGATAATGGCCGCGTGTGTCCAGCCAGGGAAGTCATTCCGCAACTGGGCGCGGTCCCCGAGCGAGTCTTCTGGGACAAGGCGGGTGCTCTAGTCGCGCTCTCGTTCCGCGCTTGCCGCAAACCTTTTGGTT>Cerrena_unicolor_1 DB=Cerun2_AssemblyScaffolds.fasta ACC=scaffold_19 REGION=121996-122268 TAX=Agaricomycotina; Agaricomycetes; Polyporales; Polyporaceae; CerrenaTGGCTGTAGTGGCCCTAAACCGGGAATGTGAGGTGGTCTAACGCTCCAACGGGTCAACTCGTCTGTGCGCTGGTTCAAGACCGTCCCCTCCAGAAAACTTGGTGACCCAAACTCCTCTCGCTTCGTAAGAGGCGGCTTGGTATGGGATAAACCGTCCTTCACAGGCCAGGGAAGCAATTCTGCAACCTGCAGGACGCGCCGAGTTAGTCTTCGGAGATCAGGACGCGTCGTCTGTTCGCGCGGATGGGCCGTGCCTCTCATAAACCTTTTATT>Cerrena_unicolor_2 DB=Cerun2_AssemblyScaffolds.fasta ACC=scaffold_15 REGION=178532-178805 TAX=Agaricomycotina; Agaricomycetes; Polyporales; Polyporaceae; CerrenaCGGCTGTAGCGGCCCCAACCCGGGAATGCAAGGTGGTCTAACGCCCCAACGGATTCACTCGTCCGTGCGCTGGTTCAAGACCGTCCCCTCCAGAAAACTTGGCGACCCTAGTTTCCTCCTGCTGCGTAAGCGGTAGCCAGGTACGGGATAAACCGTCCTTGACAGGCCAGGGAAGCAATTCTGCAACCTGCAGGACGCGCCGAGTTAGTCTTCGGAGATCAGGACGAGTCGTCTGTTCGCGCGGATGGGCCGTGCCCCTTGTAAACCTTTCGCT>Clavicorona_pyxidata_1 DB=Clapy1_AssemblyScaffolds.fasta ACC=scaffold_10 REGION=504678-504950 TAX=Agaricomycotina; Agaricomycetes; Polyporales; Auriscalpiaceae; ClavicoronaTGGCTGTAATGGCCCCAAACCGGGAATGCGAGGTGGTGTAAACTCCAACGGGCAAACCGCACGTGCGCTGAGTTCAAGACCGTCCCCTCCAGAAAACTTTGGTGTCCAACTTCCCCCGCGGCGTAAGCTGTGGCCGGACTTGGAGTAATATGACCTTGACATACCAGGGAAGTAATTCCGCAGTCTGCAGGTCTCACCGTGTTAGTCTTAGGAGATCAGGGCGATGTCGTCTGTCGCGCGAGTGGGCCGTGTCTCTCGTAAACCTTTTGCGTT>Clavicorona_pyxidata_2 DB=Clapy1_AssemblyScaffolds.fasta ACC=scaffold_23 REGION=354045-354314 TAX=Agaricomycotina; Agaricomycetes; Polyporales; Auriscalpiaceae; ClavicoronaCGGCTGTAGCGGCCCTAAGCCGGGAATGCAAGGCCGTGTTCCACCAACGGATCACTCGCGCGTGCGCTGAGTTCAAGACCGTCCCCTCCAGAAAACTCAGGTGTCCATTATCCCCCGCGGCGCAAGCCGTGGCTGGGCCTGGAGTAATCCGTCCTTGACGGGCCAGGGAAGCAATTCCGCAGCCCGCAGGCCGCACCGTGTTAGTCTTAGGAGACCAGGGCGACGTCGACTGCCGCGCGCGCGGGCCGTGCCCCTCGCAGACCTTTTGCG>Cortinarius_glaucopus DB=Corgl3_AssemblyScaffolds.fasta ACC=scaffold_378 REGION=215272-215539 TAX=Agaricomycotina; Agaricomycetes; Agaricomycetidae; Agaricales; Cortinariaceae; CortinariusCGGTTGTAATGGCCGCAAACCGGGAATGCGGGTGTCTCCTGTCAGCGAGTCAATCCATCAGCGCGCTGGTTCAAGGTCGTTCCCTCCAGAAAACTTGGTTACCACAGTCCCTCGCTTCGGAAGGAGTGATTTGGACTTGGAATAATTAGTCCTTGTCAGGCCAGGGAAGCAATTCCGCAACCTGCAGGACTTGCCGAGTTAGTCTTAGGAGATCGGAATAAACCGTCAGTTACGCGCAGTGGATCGCGGTGTCCGCAAACCTTTTTGT>Cryptococcus_vishniacii DB=Cryvi1_AssemblyScaffolds.fasta ACC=scaffold_9 REGION=626398-626671 TAX=Agaricomycotina; Tremellomycetes; Tremellales; mitosporic Tremellales; CryptococcusATCCTGTGATGGGTTTAGGGAAGGTGTTGACTGTCCTCACGGAACTAGTCATTTCAGCGCCCAAACATCCGTCTCATCCCCTTCAGAACCCGTGTTCGACTCACACCCTCGGCTCATGCCGGAGTTTTGTGATCAGCTCGGTAATTAGCGATTGGCCTTTGTGCACGACCAGGCGGGTAACCGATCAGTCACTTTCGCTAGCATGGGTTCTAACAGAGGATAAGATGAGCGGATTGGTTTGCGTAGAATGGCCCGGGTCGGTACCAACCTTTTT>Cylindrobasidium_torrendii_1 DB=Cylto1_AssemblyScaffolds.fasta ACC=scaffold_100 REGION=68417-68679 TAX=Agaricomycotina; Agaricomycetes; Corticiales; Corticiaceae; CylindrobasidiumCGGCTGTAACGGCTCAATCGGGAATGTCCTGGGTTACTGTCAGCGAATCAGTCCATCAGCGCGCTGGTTCAAGACTGTCTCCTCCAGAAAACTTGGTGTCTTCAGACCTTCTCCTCGTAAGAGTTGTTGGGTCTTGGAGTAAACAGTTCGAATCGGGCCAGGGAAGCAATTCAGCAACCCGTCGGATTGGCCGAGTTGGTCTTCGGAGATTGGGATACGTCGTCAGCCGCGCAGTGGACCGTGCCCGGGGCAAACCTTTTGCT>Cylindrobasidium_torrendii_2 DB=Cylto1_AssemblyScaffolds.fasta ACC=scaffold_26 REGION=66042-66302 TAX=Agaricomycotina; Agaricomycetes; Corticiales; Corticiaceae; CylindrobasidiumCGGCTGTAACGGCTCAATCGGGAATGCTCTGGGTTACTGTCAGCGAACCAATCCGTCAGCGCGCTGGTTCAAGACCGTCTCCTCCAGAAAACTTGGTGTCCACAGAATTACTCCTCGCAAGAGTTGTTAGTTCTTGGAATAAACAGTTCGAAACGGGCTAGAGAAGCAATTCAGCAACCCGCCGGACTGGCCGAGTTAGTCTTCGGAGATTGGGACGCGTCGTCAGCCGCGCAGCGGGTCGCGCCTGGGGCAAACCTTTTT>Cylindrobasidium_torrendii_3 DB=Cylto1_AssemblyScaffolds.fasta ACC=scaffold_100 REGION=64170-64432 TAX=Agaricomycotina; Agaricomycetes; Corticiales; Corticiaceae; CylindrobasidiumCGGCTGTAACGGCTCAATCGGGAATGTCCTGGGTTACTGTCAGCGAATCAGTCCATCAGCGCGCTGGTTCAAGACTGTCTCCTCCAGAAAACTTGGTGTCTTCAGACCTTCTCCTCGTAAGAGTTGTTGGGTCTTGGAGTAAACAGTTCGAATCGGGCCAGGGAAGCAATTCAGCAACCCGCCGGATTGGCCGAGTTGGTCTTCGGAGATTGGGATACGTCGTCAGCCGCGCAGTGGACCGCGCCCGGGGCAAACCTTTTGCT>Daedalea_quercina_1 DB=Daequ1_AssemblyScaffolds.fasta ACC=scaffold_22 REGION=395542-395813 TAX=Agaricomycotina; Agaricomycetes; Polyporales; DaedaleaTGGCTGTAGCGGCCCTATACCGGGAATGTGAGGTGGTCTAACCAATCAACGGGCTTACTCGTCTGTGCGCTGGTTCAAGGCCGTCCCCTCCGGAAAACTTGGTTGCTTTGGCTGTCCACCGCGGAAGCGGAGGCACGGCCTGGCGTAATCTGTCCTTGACTGGCCAGGGAAGCAATTCCGCAACCTGCAGGACGCGCCGAGTTAGTCTTAGGAGATAAGGACGTGCCGTCTGTTCGCGTAGATGGGCCGTGCCCTTCACAAACCCTTTTGTT>Daedalea_quercina_2 DB=Daequ1_AssemblyScaffolds.fasta ACC=scaffold_3 REGION=221218-221486 TAX=Agaricomycotina; Agaricomycetes; Polyporales; DaedaleaTGGCTGTAGTGGCCCTACATCGGGAATGCGAGACGGTTCAACTCAGCGGCTGAGTCCATGTGTGCGCTGGTTCAAGGCCGTCCCCTCCGGAAAACTTGGTGTTCTGTTAGGTTCCTTCTCGGAAGAGAAGACAACCTTCAGAGTAAACTGACCTCCGTGGGCCAGGGAAGCAATTCCGCAACCCAGAGGTCGGGCCGAGTTAGTCTTGGGAGACAAGGACTGTCGTCAGTTCGCGCAGTGGATCGTGCCTCTCGCAAACTCTTTTGATT>Dendrothele_bispora DB=Denbi1_AssemblyScaffolds.fasta ACC=scaffold_34 REGION=272659-272924 TAX=Agaricomycotina; Agaricomycetes; Corticiales; Corticiaceae; DendrotheleTGGTTGTAACGGCCCGAAGCTGGGAATGTGGATTGTTCTATACAGCGAGCAACACCCCGAGTGCGCTGGTTCAAGACTGTCCCCTCCAGAAAACTTGGTGTTCACAGTCCTTCGCGCTGTAAAGCATGAAATGGACTTGGAATAATCGGTACTTGTCAGACCAGGGAAGCAATTCCGCAATCTGCAGTAACCCACCGAGTTAGTCTTAGGAGATCAGGGCAGGATTCAGACGCGCTGGGGTGCGCGCAGTCCATAAACCTTTTGTT>Dioszegia_cryoxerica_1 DB=Diocr1_AssemblyScaffolds.fasta ACC=scaffold_100 REGION=65211-65495 TAX=Agaricomycotina; Tremellomycetes; Tremellales; mitosporic Tremellales; DioszegiaTCGGTTTTACGCCTTTGAGAAGATGGTTCCATTTTCGTCCACGGATTTTCCTGCCCAGCGCGACAGTTGAATACCTTTTCCTCGTAGACTGACGAGTTCGCATCACGACTCTGGCACACGCCTGGAGGACTTTAGTGATTCTAGCGGTAATCAGATCAGCTTGTACTCTCGCAAGAGAGTCGGCCATGCCGGTAACGGAGCAGCCGCCTGATCTGGCTTGGAAGTCTTTGCGAGATAAGAAGGGTATTGGACGCGTAGGGAGGCCCGGGTGGCCATCAACTCTTT>Dioszegia_cryoxerica_2 DB=Diocr1_AssemblyScaffolds.fasta ACC=scaffold_59 REGION=94705-94988 TAX=Agaricomycotina; Tremellomycetes; Tremellales; mitosporic Tremellales; DioszegiaTCGGTTTTACGCCTTTGAGAAGATGGTTCCATTTTCGTCCACGGATTTTCCTGCCCAGCGCGACAGTTGAATATCTTTTCCTCGTAGACTGACGAGTTCGTATCACGACTCTGGCTCACGCCTGGAGGACTTTAGTGATCTAGCGGTAATCAGATCAGCTTGTACTCTCGCAAGAGAGTCGGCCATGCCGGCAACGGAGCAGCCGCCTGATCTGGCTTGGAAGTCCTTGCGAGATAAGAAGGGTATTGGACGCGCAGGGAGGCCCGGGTGGCCGTCAACTCTTT>Fistulina_hepatica DB=Fishe1_AssemblyScaffolds.fasta ACC=scaffold_66 REGION=160947-161209 TAX=Agaricomycotina; Agaricomycetes; Agaricomycetidae; Agaricales; Fistulinaceae; FistulinaTAGCTGTAACGGCTAAACTGGGGATGTCGTGGGAACTTTCATCGGGTGTGGTCCGAGTGCGCTGGTTCAAGACCATCCCCTCCAGATAATTTGGTTTCCGCAATTGCTCGCCTTGTAGAAGGTGGCCTCAATTCGGAATAATATGGGTTTGTCAGGCCAGGGGAGCAATCTCGCAACCTGCAGTCCTCGCCAAATTAGTCGTAGGAGATCAGGGTGTGTCGTCAGTCGCGCAGGGCTTCGAGCCTATGGCAAACCTTTTCATT>Guyanagaster_necrorhiza DB=Guyne1_AssemblyScaffolds.fasta ACC=scaffold_1 REGION=2328412-2328678 TAX=Agaricomycotina; Agaricomycetes; Agaricomycetidae; Agaricales; Physalacriaceae; GuyanagasterTGGCTGTAACGGCCCTAAAACTCGGGATGTGAGTGGTTTATATCAGCGAAGCAATCCGCTTGTGCGCTGGTTCAAGACTGTCCCCTCCAGAAAACTTGGTGTCCACAGTTTCTCGCTCTGTAAAGGGTGGTCTGGGCTTGGAATAATCAGTCCTTGATTGGCTAGGGAAGCAATTCCGCAACCAGCAGGACTAGCCGAGTTAGTCGCAGGAGATCAGGGCAAGTCGTCAGTTCGCGCTACGGACCGTGCCTCTCATAGACTTTTTTT>Gymnopus_androsaceus_1 DB=Gyman1_AssemblyScaffolds.fasta ACC=scaffold_108 REGION=100964-101231 TAX=Agaricomycotina; Agaricomycetes; Agaricomycetidae; Agaricales; Tricholomataceae; GymnopusTGGCTGTAATGGCCCGAACGTGGGAATATGGAGTGGTTTACTCAGCGAGCAAAACCATGGTGCGCGGGTTCAAGCACTGTCCCCTCCAGAAAACTTGGTGTTCATCGCCTCCCGCTCTGCAAAGGGTGGTTCAGGGCATGGAATAATGCGCACTTGTCAGGCCAGGGAAGCAATTCCGCAACCTGCAGTGCCGACCGAGTTAGTCTTAGGAGATCAGGGCAGGGCGCCAGACGCGCTGTGTGTTCGTGCCCTCCATAAACCTTCTTTC>Gymnopus_androsaceus_2 DB=Gyman1_AssemblyScaffolds.fasta ACC=scaffold_108 REGION=129126-129393 TAX=Agaricomycotina; Agaricomycetes; Agaricomycetidae; Agaricales; Tricholomataceae; GymnopusTGGCTATAGTGGCCCGAACGTGGGAATATGGAGTGGTTTACTCAGCGAGCAAAACCATGGTGCGCGGGTTCAAGCACTGTCCCCTCCAGAAAACTTGGTGTTCATCGCCTCCCGCTCTGCAAAGGGTGGTTCTGGGCATGGAATAATGTGCACTTGTCAGGCCAGGGAAGCAATTCCGCAACCTGCAGTGCCGACCGAGTTAGTCTTAGGAGATCAGGGCAGGGCGCCAGACGCGCTATGTGTTCGTGCCCTCCATAAACCTTCTTTC>Gymnopus_androsaceus_3 DB=Gyman1_AssemblyScaffolds.fasta ACC=scaffold_155 REGION=79559-79823 TAX=Agaricomycotina; Agaricomycetes; Agaricomycetidae; Agaricales; Tricholomataceae; GymnopusTGGCTGTAGTGGCCCGAGCTTGGGAATGCGAGGCGGCTTACTTCAGCGGTAACACCTCACGCGCGCTGGTTTCAATCCTGCCCCCTCCAGAAAACTTGGTGTTCATTACTCCCCGCTTTGCAAAGAGTGGTTCCGAGTTTGGAATAATGTGCGCTAGTCAGGCCAGGGAAGCAATTCCGCAACCTGCAGTGCCGACCGAGTTAGTCTTAGGAGATCAGGGTAGGTGGCAGACGCGCGGAGTGTGCGCGCCCCTCACAAACCTTTT>Gymnopilus_chrysopellus_1 DB=Gymch1_AssemblyScaffolds.fasta ACC=scaffold_118 REGION=54129-54395 TAX=Agaricomycotina; Agaricomycetes; Agaricomycetidae; Agaricales; Cortinariaceae; GymnopilusCGGCTGTAGTGGCCGCAAACCGGGAATGCGGGAATTTCCTCTCAGCGATCAAAGCTACCAGCGCGCTGTTTCAAGGCTAGGCCCTCCAGAAAACTTGGTTTCCTCAGTTCCTCGCTCCGTAAGGGGTGGCTTGAACTAGGAATAATTTGTCCTTGTCAGGTCAGGAAAGCAATTTCGCAGCCTGCAGGACTCGCCGAGTTAGTCTTCGGAGATCAGACTAGACCGTCAGTACGCGCAGTGGTTCGCGGTTTCTGCAAACCTTTTACT>Gymnopilus_chrysopellus_2 DB=Gymch1_AssemblyScaffolds.fasta ACC=scaffold_25 REGION=49892-50158 TAX=Agaricomycotina; Agaricomycetes; Agaricomycetidae; Agaricales; Cortinariaceae; GymnopilusCGGTTGTAGTGGCCGCAAACCGGGAATGTGGGAGTTTCCTTCCAGCGAATCAAGCCATCAGCGCGCTGTTTCAAGGCTAGGCCCTCCAGAAAACTTGGTTTCCACAGTCCCTCGCCCTGAAGAGGGTGACCTGGGCTTGGAATAATTTGTCCTTGACAGGCTAGGGAAGCAATTCCGCAACCTGCAGGACTTGCCGAGTTAGTCTTAGGAGATCAGACTAGACCGTCAGTTCGCGCAGTGGCTCGCGGCTCCTGCAAACCTTTTGCT>Gymnopus_luxurians_1 DB=Gymlu1_AssemblyScaffolds.fasta ACC=scaffold_11 REGION=577721-577985 TAX=Agaricomycotina; Agaricomycetes; Agaricomycetidae; Agaricales; Tricholomataceae; GymnopusTGGTTGTAATGGCCCGAACCTGGGAATGTGAGGCGGCTTACTTCAGCGAATCAACCATTCGTGCGCTGGTTCAAGACTGTCCCCTCCAGAAAACTTGGTGTCCATCACTCCCCGCTTTGTAAAAGGTGGTAATGAGGTTGGAATAATGAGTGCTTGTCAGGCCAGGGAAGCAATTCCGCAACCTGCAGCGCCGACCGAGTTAGTCTTAGGAGAACAGGGCATGTCGGCAGACGCGCGGTGTGTGCGCGCCCTTCACAAACCTTTT>Gymnopus_luxurians_2 DB=Gymlu1_AssemblyScaffolds.fasta ACC=scaffold_11 REGION=638842-639106 TAX=Agaricomycotina; Agaricomycetes; Agaricomycetidae; Agaricales; Tricholomataceae; GymnopusTGGTTGTAATGGCCCGAACCTGGGAATGTGAGGCGGCTTACTTCAGCGAATCAACCATTCGTGCGCTGGTTCAAGACTGTCCCCTCCAGAAAACTTGGTGTCCATCACTCCCCGCTTTGTAAAAGGTGGTAATGAGGTTGGAATAATGAGCGCTTGTCAGGCCAGGGAAGCAATTCCGCAACCTGCAGTGCCGACCGAGTTAGTCTTAGGAGAACAGGGCATGTCGGCAGACGCGCGGTGTGTGCGCGCCCTTCACAAACCTTTT>Gyrodon_lividus DB=Gyrli1_AssemblyScaffolds.fasta ACC=scaffold_2 REGION=949355-949621 TAX=Agaricomycotina; Agaricomycetes; Agaricomycetidae; Boletales; Paxilineae; Gyrodontaceae; GyrodonTGGCTGTAATGGCCCTAAACCGGGAATGTGAGATGGTTCTTTACAGCGAACAAATCCACGAGTGCGCTGGTTCAAGACTGTCCCCCTCCAGAAAATTTGGTGTCCACAGTTTCTCACGCTGTAAAGTGTGAACGAACTTGGAATAATTGGTCCTTGACTGGCCAGGGAAGCAATTCCGCAACCTGCAGGATCTGCCGAATTAGTCCTAGGAGATCAGGGCATGTCGTCAGACGCGCTGTCGATCGCGCCTCTCGCAAACCTTTTGTC>Hebeloma_cylindrosporum DB=Hebcy2_AssemblyScaffolds.fasta ACC=scaffold_1 REGION=2327037-2327304 TAX=Agaricomycotina; Agaricomycetes; Agaricomycetidae; Agaricales; Cortinariaceae; HebelomaCGGTTGTAATGGCCGCAAATCGGGAATGCGGGTGTCACCTTTCAGTGACCAAAGCCATCAGCGCGCTGGTTCAAGGCCCTCCCCTCCAGAAAACTTGGTTTCCACAGTCTCTCGCACCGTAAGGTGTGATTTGGACTTGGAATAATCTGTCCTTGTCAGGCCAGGGAAGCAATTCCGCAACCTGCAGGACTCGCCGAGTTAGTCTTAGGTAGATCAGGAGGGACCGTCAGTTCGCGCAGTGGCTCGCGGTGTCCGCAAACCTTTTTTG>Hydnomerulius_pinastri DB=Hydpi2_AssemblyScaffolds.fasta ACC=scaffold_17 REGION=146486-146751 TAX=Agaricomycotina; Agaricomycetes; Agaricomycetidae; Boletales; Paxilineae; Paxillineae incertae sedis; HydnomeruliusTGGCTGTAGTGGCCCTAAACCGGGAATGCGAGATGGTTCTTCACAGCGACCAAACCCACGAGTGCGCTGGTTCAAGACTGTCCCCTCCAGAAAATTTGGTGACCAACGTTTCTCGCACTGTAAAGTGTGAACAAACTTGGAATAATTGGTCCTTGACTGGCCAGGGAAGCAATTCCGCAACCTGCAGGATCTGCCGAGTTAGTCGTAGGAGATCAGGGCATGTCGTCAGACGCGCAGTGGGTCGCGCCGCTCGCAAACCTTTTGCT>Hypholoma_sublateritium DB=Hypsu1_AssemblyScaffolds.fasta ACC=scaffold_44 REGION=273296-273562 TAX=Agaricomycotina; Agaricomycetes; Agaricomycetidae; Agaricales; Strophariaceae; HypholomaCGGTTGTAATGGCCGCAAACCGGGAATGCGGGTGTCTTATGTCAGCGAACTAAGCCATCAGTGCGCAGGTTCGAGTTGGCGCCCTCCAGAAAACTTGGTTTCTACAGTTCCTCGCTCCGTAAGGGGTGGTTTGAGCTTAGAATAATATGTCCTCGTCAGGCCAGGGAAGCAATTCCGCAACCTGCTGGACTCGCCGAGTTAGTCTTAGGCAGATCAGTGCCTCACCAATGTCGCGCAGTGGCTCGCGGCTCCCGCAAACCTTTTTGT>Laccaria_amethystina_1 DB=Lacam1_AssemblyScaffolds.fasta ACC=scaffold_41 REGION=18037-18302 TAX=Agaricomycotina; Agaricomycetes; Agaricomycetidae; Agaricales; Tricholomataceae; LaccariaAGGCTGTAGTGGCCCTAAACCGGGAATGCGGGTGGTTTCTTTCATCGGTCAACTCCACGAGTGCGCTGGTTCAAGGCTGTCCCCTCCAGAAAACTTGGTTACCACAGACTCTCGCCCCGTAAGGTGCGATTCGGTCTCGGAATAATCGGTCCTTGACAGGCCAGGGAAGCAATTCCGCAACCTGCAGGACTTGCCGAGTTAGTCTTCGGAGATCAGGGCATGTCGTCAGTCGCGCATTGGAGCGGGCCGCCCGCAAACCTTTTTCA>Laccaria_amethystina_2 DB=Lacam1_AssemblyScaffolds.fasta ACC=scaffold_173 REGION=35255-35518 TAX=Agaricomycotina; Agaricomycetes; Agaricomycetidae; Agaricales; Tricholomataceae; LaccariaAGGTTGTAATGGCCTCAATCAGGAATGCAGGTGGTTCTTCTCATCGATTTCCTCCATGAGTGCGCTGGTTCAAGACTGTCCCCTCCAGAAAACTTGGTTACCACAGATCCCATTCCGTAGGGAGTGAACCGATCTTGGAATAATTCGTCCATGACAGGCCAGGGAAGCAATTCCGCAACCTGCTGGACCCGCCGAGTTAGTCTTCGGAGATCAGGGCATGTCGTCAGACGCGCAATGGAGCGTGCCTCCCGCAAACCTTTTACA>Laetiporus_sulphureus DB=Laesu1_AssemblyScaffolds.fasta ACC=scaffold_3 REGION=180794-181060 TAX=Agaricomycotina; Agaricomycetes; Polyporales; LaetiporusTGGCTGTAATGGCCCTAAACCGGGAATGCGAGGTGGTTCTTGTTGGCGGTAAAAACTCATTTGCGCGCTGGTTCAAGACTGTCCCCTCCAGAAAACTTGGTATTTGCGTGGTTTCTAGTCGGAAGACCGGATTGCCAGCAAAGTAAACCGGTCTCCTTGGGCCAGGGAAGCAATTCTGCAACCCAGAGATCGGGCCGAGTTAGTCTTAGGAGATAAGGACTGTCATCAGTTCGCGCAGTGGGTCGTGCCTCTCGCAAACCTTTTGCT>Leucogyrophana_mollusca DB=Leumo1_AssemblyScaffolds.fasta ACC=scaffold_00030 REGION=126749-127014 TAX=Agaricomycotina; Agaricomycetes; Agaricomycetidae; Boletales; Boletales incertae sedis; LeucogyrophanaCGGCTGTAATGGCCCTAAACCGGGAATGCGAGATGGTTCTTTACAGCGAATTCACCCACGAGTGCGCTGGTTCAAGACTGTCCCCTCCAGAAAACTTGGTGACCACAGTCTCTCGCGCCGTAAGGTGTGATCGGACTTGGAATAATCGGTCCTTGACAGGTCAGGGAAGCAATTCCGCACCCTGCAGGATCGGCCGAGTTAGTCGTAGGAGATCGGGGCATGTCGTCAGTCGCGCAGTGGGTCGCGCCGCTCGCAAACCTTTCTTT>Macrolepiota_fuliginosa DB=Macfu1_AssemblyScaffolds.fasta ACC=scaffold_434 REGION=17064-17328 TAX=Agaricomycotina; Agaricomycetes; Agaricomycetidae; Agaricales; Agaricaceae; MacrolepiotaCGGCTGTTGTGGCCCAATCCGGAATGAGTGGGGTTCCTCTCAGCGGGTTAAGCCATCAGTGCGCTGGTTCAAGACTGTCCCCTCCAGAAAACTTGGTTTCCACAGTCCCTCGCCCCGTAAGGAGTGATCCGGACTTGGAATAATTTGCCCTTGACAGGCCAGGGAAGATAATTCCGCAACCTGCAGGACTTGCCGAGTTAGTCTTAGGAGATCAGGGCATGTCGTCAGTCGCGCAGTGGTTCTCGCCCTGCTCAAACCTTTTACC>Neolentinus_lepideus DB=Neole1_AssemblyScaffolds.fasta ACC=scaffold_30 REGION=177732-178003 TAX=Agaricomycotina; Agaricomycetes; Gloeophyllales; Gloeophyllaceae; NeolentinusTGGCTGTAATGGCTCAAACCGGGAATGCGAGGTGGTCTAAAGTTACAACGGGTTCCCCCGCTCGTGCGCTGAGTTCAAGGCCGTCCCCTCCAGAAAACTTGGTAGCCGTCGTTCGCCGCGTCGGAAGACATGGCTGGGCTCGGAATAATCGGTCCGAGACAGGCCAGGGAAGCAATTCTGCAACCTGCCGGACCCGCCGAGTTAGTCTTAGGAGATCAGGATGCGTCGTCTGTCGCGCGAGTGGGCCGTGCCCTTCGCAAACTTTTTACGTT>Obba_rivulosa DB=Obbri1_AssemblyScaffolds.fasta ACC=scaffold_71 REGION=75833-76105 TAX=Agaricomycotina; Agaricomycetes; Polyporales; Meruliaceae; ObbaTGGCTGTAGCGGCCCTAATCCGGGAATGCGAGGTGGTCTAAAGCCCCAACGGACAAACCCGTCTGTGCGCTGGTTCAAGACCGTCCCCTCCAGAAAACTTGGTAGCCTTTGGCTGTCCGTGGCGTAGGCCTCTGGCACGGCCTGGCGTAATCTGTCCTGCACGGGCCAGGGAAGCAATTCCGCAACTCACAGGATGGGCCGAGTTAGTCTTAGGAGATAAGGACGAGTCGTCCGTTCGCGCAGATGGGCCGTGCCCTTCGCAAACCTTTTTCA>Panellus_stipticus DB=Panst_KUC8834_1_1_AssemblyScaffolds.fasta ACC=scaffold_62 REGION=13432-13698 TAX=Agaricomycotina; Agaricomycetes; Agaricomycetidae; Agaricales; Tricholomataceae; PanellusCGGCTGTAACGGCCCTAAACCGGGAATGTGATTGGTTCTTCTCATCGGCAAACATCATCTGTGCGCTGGTTCAAGACCGTCCCCTCCAGAAAACTTGGTTTCCACAATTCTCCGCCCCGGAAGGGATGGTTCGGATTTGGAATAATCTGTCCTTGTCAGGCCAGGGAAGCAATTCTGCAACCTGCAGGACTCGCCGAGTTAGTCTTCGGAGACAAGGGCTGGTCGTCAGTTCGCGCTGTGATGCGAGCCTTTCACAAACCTTTTTGC>Paxillus_involutus_1 DB=Paxin1_AssemblyScaffolds.fasta ACC=scaffold_339 REGION=6316-6582 TAX=Agaricomycotina; Agaricomycetes; Agaricomycetidae; Boletales; Paxilineae; Paxillaceae; PaxillusTGGCTGTAATGGCCCAAACCCGGGAATGCGAGATGGTTCTTTACAGCGAACAAACTCACGAGTGCGCTGGTTCAAGACTGTCCCCTCCAGAAAATTTGGTGACCACAGTTTCTCGCGCCGCAAGGTGTGAACGAACTTGGAATAATTGATCCTTGACTGGCCAGGGAAGAAATTCCGCAACCTGCAGGATCTGCCGAATTAGTCGTAGGAGATCAGGGCATGTCGTCAGACGCGCTGTCGGTCGTGCTGCTCGCAAACCTTTTGCTT>Paxillus_involutus_2 DB=Paxin1_AssemblyScaffolds.fasta ACC=scaffold_9 REGION=128037-128302 TAX=Agaricomycotina; Agaricomycetes; Agaricomycetidae; Boletales; Paxilineae; Paxillaceae; PaxillusTGGTTGTAATGGCCCTAACTCGGGAATGCGAGATGGTTCTATACAGCGAACAAACTCACGAGTGCGCTAGTTCAAGACTGTCCCCTCCAGAAAATTTGGTGTCCACAGTTTCTCGCGCCGCAAGGCATGATCGAACTTGGAATAATTGATCCTTGATTGGCCAGGGAAGAAATTCCGCAACCTGCAGGATCTGCCGAATTAGTCGTAGGAGATCAGGCCATGTCGTCAGACGCGCTGTGAGTCGCGCCCCTCGCAAACCTTTTGTT>Paxillus_rubicundulus DB=Paxru1_AssemblyScaffolds.fasta ACC=scaffold_288 REGION=16516-16781 TAX=Agaricomycotina; Agaricomycetes; Agaricomycetidae; Boletales; Paxilineae; Paxillaceae; PaxillusTGGCTGTAATGGCCCAACTCGGGAATGCGAGATGGTTCTATACAGCGAACAAACCCATGAGTGCGCTGGTTCAAGACTGTCCCCTCCAGAAAATTTGGTGACCACAGTTTCTCGCGCCGCAAGGTGTGAACGAACTTGGAATAATTGATCCTTGACTGGCCAGGGAAGAAATTCCGCAACCTGCAGGATCTGCCGAATTAGTCGCAGGAGATCAGGGCATGTCGTCAGACGCGCTGTGGGTCGTGCCGCTCGCAAACCTTTTGCTT>Phlebia_brevispora DB=Phlbr1_AssemblyScaffolds.fasta ACC=scaffold_16 REGION=718759-719030 TAX=Agaricomycotina; Agaricomycetes; Corticiales; Corticiaceae; PhlebiaTGGCTATAGCGGCCCTCTGCCGGGAATGTGAGGCGGTCTAAATTCTCAACGGAACCCCTGCTCTGTGCGCTGGTTCAAGACGGATCCCTCCAGAAAACTTGGTGACCCTTTGTCGCTCGCAACGCAAGTCGTGAGTCGACTCGGGATAATCCGTCCTTGACTGGCCAGGGAAGCAATTCCGCAACCTCCAGGACGCGCCGAGTTAGTCGTCGGAGACAAGGTCCTGTCGTCTGTTCGCGCAGCGAAGCCGTGCCCTTCATAAACCTTTGTTT>Phlebiopsis_gigantea DB=Phlgi1_AssemblyScaffolds.fasta ACC=scaffold_19 REGION=45071-45344 TAX=Agaricomycotina; Agaricomycetes; Polyporales; Phanerochaetaceae; PhlebiopsisTGGCTGTAGTGGCCCTAAACCGGGAATGTGAGGTGGTCTAAATCTCCAACGGACTTACTCGTCTGTGCGCTGGTTCAAGACCGTCCCCTCCAGAAAACTTGGTGTCCCGTTTAACCGCCCGCGGCGTAGGCTGTGGAACGGTGCGGGATAACTCGTCCTTGACTGGCCAGGGAAGTAATTCCGCAACCTGCAGGACGCGCCGAGTTAGTCGTCGGAGATAAGGATGAGTCGTCTGTTCGCACAGGTGGGCCGTGCCTCTCGCAAACCTTTTGTC>Piloderma_croceum_1 DB=Pilcr1_AssemblyScaffolds.fasta ACC=scaffold_00010 REGION=396490-396757 TAX=Agaricomycotina; Agaricomycetes; Agaricomycetidae; Atheliales; Atheliaceae; PilodermaTGGCTGTAATGGCCCCAAACCGGGAATGCGAGGCGGTTCTTCTCAGCGGTCAAACTTACAAGTGCGCTGGTTCAAGTCCCTCCCCTCCAGAAAACTTGGTTTCCACAGCTTCCCGCACCTGAAGGTGTGGTCCGAGCTTGGAATAATAGGTCCTTGACAGGCCAGGGAAGCAATTCCGCAATCTGCAGGACTCGCCGAGTTAGTCTTCGGAGACCAGGAGGAGGCGTCAGTTCGCGCTGTGGGTCGTGCTCTTCGCAAACCTTTTGCT>Piloderma_croceum_2 DB=Pilcr1_AssemblyScaffolds.fasta ACC=scaffold_00010 REGION=172993-173260 TAX=Agaricomycotina; Agaricomycetes; Agaricomycetidae; Atheliales; Atheliaceae; PilodermaTGGCTGTAATGGCCCCAAACCGGGAATGCGAGGCGGTTCTTCTCAGCGGTCAAGCCCACAAGTGCGCTGGTTCAAGTCCCTCCCCTCCAGAAAATTTGGTCTCCACAGTTTCCCGCACCTGAAGGTGTGGTCCGAGCTTGGAATAATAGGTCCTTGACAGGCCAGGGAAGCAATTCCGCAACCTGCAGGACTCGCCGAATTAGTCTTCGGAGATCAGGAGGAGGCGTCAGTTCGCGCTGTGGGTCGTGCTCTTCGCAAACCTTTTGCC>Pisolithus_microcarpus DB=Pismi1_AssemblyScaffolds.fasta ACC=scaffold_196 REGION=46025-46291 TAX=Agaricomycotina; Agaricomycetes; Agaricomycetidae; Boletales; Sclerodermatineae; Pisolithaceae; PisolithusTGGCTGTAATGGCCCACCACTTGGGAATGCGAGGTGGTTTATGTCAGCGAGCTCATCCGCGAGTGCGCTAGTTCAAGACTGTCCCCTCCAGAAAACTTGGTGATCATTTGCGTCTCGTGCTTCGTAAGAGGTACTCGGACATGGAATAATTGGACCTCGTCTGGCCAGGGAAGCAATTCCGCATCCAGCTGGTCCTGCCGAGTTAGTCGTAGGAGATCAGGACACGTCGTCAGTCGCGCAGCGGATCGCGCCCCTTGCAAACCTTTT>Pisolithus_tinctorius_1 DB=Pisti1_AssemblyScaffolds.fasta ACC=scaffold_55 REGION=358591-358858 TAX=Agaricomycotina; Agaricomycetes; Agaricomycetidae; Boletales; Sclerodermatineae; Pisolithaceae; PisolithusCGGCTGTAATGGCCCTCCATCGGGAATGCGAGGTGGTTTACGTCAGCGAACTCATCCACGAGTGCGCTAGTTCAAGACTGTCCCCTCCAGAAAACTTGGTTGTCATTTCGTCTTGTGCCTCGTAAGAGGTATTTGGACATGGAATAATTGGACCTTGTCTGGCCAGGGAAGCAATTCCGCATCCAGCAGGTCCTGCCGAGTTAGTCGTAGGAGATCAGGACACGTCGTCAGCCGCGCAGTGGATCGCGCCCTTCGCAAACCTTTTTAC>Pisolithus_tinctorius_2 DB=Pisti1_AssemblyScaffolds.fasta ACC=scaffold_309 REGION=6719-6986 TAX=Agaricomycotina; Agaricomycetes; Agaricomycetidae; Boletales; Sclerodermatineae; Pisolithaceae; PisolithusCGGCTGTAATGGCCCTCCATCGGGAATGTGAGGTGGTTTATGTCAGCGAACTCATCCACGAGTGCGCTAGTTCAAGACTGTCCCCTCCAGAAAACTTGGTTGTCATTTCGTCTTGTGCCTCGTAAGAGGTATTTGGACATGGAATAATTGGACCTTGTCTGGCCAGGGAAGCAATTCCGCATCCAGCAGGTCCTGCCGAGTTAGTCGTAGGAGATCAGGACACGTCGTCAGCCGTGCAGTGGATCGCGCCCTTCGCAAACCTTTTTAC>Plicaturopsis_crispa DB=Plicr1_AssemblyScaffolds.fasta ACC=scaffold_4 REGION=1129661-1129925 TAX=Agaricomycotina; Agaricomycetes; Agaricomycetidae; Agaricales; Agaricales incertae sedis; PlicaturopsisTGGTTGTAATGGCCCAAAGTCGGGAAGGCGTGGGAATCTCTCAGCGGTAACGCCACGAGTGCGCTGGTTCAAGACCGTCCCCTCCAGAAAACTTGGTATCCACAGTCTCCCGCTCCGCAAGGCGTGGATTGGACTTGGAATAATCGGTCCTTGACAGGCCAGGGAAGCAATTCCGCAACCTGCAGGACTCGCCGAGTTAGTCTTCGGAGATCAGGACGCGTCGTCAGTACGCGCTGTGGTTCGTGACTCGCGCCAACCTTTTGCT>Polyporus_arcularius DB=Polar1_AssemblyScaffolds.fasta ACC=scaffold_156 REGION=35809-36079 TAX=Agaricomycotina; Agaricomycetes; Polyporales; Polyporaceae; PolyporusTGGCTGTAATGGCCCTAAACCGGGAATGTGAGGTGGTCTAAAGCTCCAACGGACTCAGTCGTCTGTGCGCTGGTTCAAGACCGTCCCCTCCAGAAAACTTGGTTGCCTCGAGTCGCCCGCTCCGTAAGGAGTGGATTGACCTGGTATAATACGCCCTTGACTGGCCAGGGAAGCAATTCCGCAACCTGCAGGGCGTGCCGAGTTAGTCTTAGGAGATCAGGACGAGTCGTCAGTCGCGCAGGTGGCCCGTGCCCCTCATAAACCCTTCCTT>Pycnoporus_cinnabarinus DB=Pycci1_AssemblyScaffolds.fasta ACC=scf_185000 REGION=185044-185314 TAX=Agaricomycotina; Agaricomycetes; Polyporales; Polyporaceae; PycnoporusTGGCTGTAACGGCCCTAAACCGGGAATGTGAGGTGGTCTAACGATCCAACGGGCTCAGTCGTCTGTGCGCTGGTTCAAGACCGTCCCCTCCAGAAAACTTGGTTACCTCGCACCGCCCGCTCCGTATGGGATGGGCTGGGCTGGTATAATACGCCCTTGATTGGCCAGGGAAGCAATTCCGCAACCTGCAGGGCGTGCCGAGTTAGTCTTAGGAGATCAGGACGAGTCGTCTGTCGCGCGGATGACCCGTGCCCCTCACAAACCCTTCCTT>Pycnoporus_coccineus_1 DB=Pycco1662_1_AssemblyScaffolds.fasta ACC=scaffold_21 REGION=42969-43240 TAX=Agaricomycotina; Agaricomycetes; Polyporales; Polyporaceae; PycnoporusTGGCTGTAACGGCCCTAAACCGGGAATGTGAGGTGGTCTAACGATCCAACGGGCTCAGTCGTCTGCGCGCTGGTTCAAGACCGTCCCCTCCAGAAAACTTGGTTACCTCGCATCGCCCGCTCCGTAAGGGATGGGTTGGGCTGGTATAATACGCCCTTGACTGGCCAGGGAAGCAATTCCGCAACCTGCAGGGCGTGCCGAGTTAGTCTTAGGAGATCAGGACGAGTCCCCTGTTCGCGCAGATGACCCGTGCCCCTCACAAACCCTTTCTT>Pycnoporus_coccineus_2 DB=Pycco1_AssemblyScaffolds.fasta ACC=scaffold_22 REGION=91307-91578 TAX=Agaricomycotina; Agaricomycetes; Polyporales; Polyporaceae; PycnoporusTGGCTGTAACGGCCCTAAACCGGGAATGTGAGGTGGTCTAACGATCCAACGGGCTCAGTCGTCTGTGCGCTGGTTCAAGACCGTCCCCTCCAGAAAACTTGGTTACCTCGCACCGCCCGCTCCGTAAGGGATGGGTTGGGCTGGTATAATACGCCCTTGACTGGCCAGGGAAGCAATTCCGCAACCTGCAGGGCGTGCCGAGTTAGTCTTAGGAGATCAGGACGAGTCCCCTGTTCGCGCGGATGGCCCGTGCCCCTCACAAACCCTTTCTT>Pycnoporus_sanguineus DB=Pycsa1_AssemblyScaffolds.fasta ACC=sc_7180000650847 REGION=72879-73150 TAX=Agaricomycotina; Agaricomycetes; Polyporales; Polyporaceae; PycnoporusTGGCTGTAGCGGCCCTAAACCGGGAATGTGAGGTGGTCTAACGATCCAACGGGCTCAGTCGTCTGTGCGCTGGTTCAAGACCGTCCCCTCCAGAAAACTTGGTTACCTCGCGCCGCCCGCTCCGCAAGGAATGGGTTGGGCTGGTATAATACGCCCTTGACTGGCCAGGGAAGCAATTCCGCAACCTGCAGGGCGTGCCGAGTTAGTCTTAGGAGATCAGGACGAGTCCCCTGTTCGCGCAGATGACCCGTGCCCCTCACAAACCCTTTCTT>Ramaria_rubella_1 DB=Ramac1_AssemblyScaffolds.fasta ACC=scaffold_459 REGION=17742-18012 TAX=Agaricomycotina; Agaricomycetes; Phallomycetidae; Gomphales; Gomphaceae; RamariaTGGCTGTAATGGCCCAACTGGGAATGCGAAGCGGTGTTATCCGCAACGGACAACCGAGTCGTGCGCTGGTTCAAAGCCCTCCCCTCCAGAAAATTTGGTGTCGATATTAGTCCTCCGCACGGCAACGTGTGCCTGGACGTCGAATAATGGATTGTCGATCGGTCAGGGAAGTCATTCCGCAGCCGCGACAGTGCGCCGAGTTAGTCTCAGGAGATCAGGTGGTGTCTTCTAGTCGCGCGACTGGGCCGTGCCCTTCGCAGACTCTTTTGTT>Ramaria_rubella_2 DB=Ramac1_AssemblyScaffolds.fasta ACC=scaffold_301 REGION=108934-109205 TAX=Agaricomycotina; Agaricomycetes; Phallomycetidae; Gomphales; Gomphaceae; RamariaTGGCTGTAATGGCCCAAGTGGGAATACGAAGCGGTGTCACCCGCAACGGACAACCCAATCCGTGAGCTGGTTCGAATCCATCCCCTCCAGCAAATTTGGTATCGATATTAGTCCTTCGCGTGGCAACGTGTGGCTGGACGTGGAATAATGAATTGTCGATCGGTTAGGGAAGTCATTCCGCAGCCGCGACACTCTGCCGAGTTAGTCTCAGGAGATCAGGTGGTGTCTTCTAGTCGCCCAATTCGGCCGTGCCCTTCGCAAACTCTTTTATT>Rhizopogon_vinicolor DB=Rhivi1_AssemblyScaffolds.fasta ACC=scaffold_260 REGION=17362-17629 TAX=Agaricomycotina; Agaricomycetes; Agaricomycetidae; Boletales; Suillineae; Rhizopogonaceae; RhizopogonCGGCTGTAATGGCCCTAAACCGGGAATGCGAGATGGTTCTCTCCAGCGATCCAACCCATGAGTGCGCTGGTTCAAGACTGTCCCCTCCAGAAAACTTGGTGTCCACAGTTCCTTGCCCCGAAAGGTGTAATCGGACTTGGAATAATCGGTCCTTGACAGGCCAGGGAAGCAATTCCGCAACCTGCTGGATCTGCCGAGTTAGTCGTAGGAGATCAGGGCATGTCATCAGCCGCGCTGTGGGTCGCGCTGCTCGCAAACCTTTTTCATT>Rickenella_mellea DB=Ricme1_AssemblyScaffolds.fasta ACC=scaffold_106 REGION=26679-26949 TAX=Agaricomycotina; Agaricomycetes; Agaricomycetidae; Agaricales; Tricholomataceae; RickenellaTGGCTGTAATGGCCCTAAACCGGGAATGTGAGGTGGTGTATCCCTCAACGGTCAACGTGCTTGTGCGCTGAGTTCAAGACCGACCCCTCCAGAAAACTTGGGTGTCCTACGCTCCTCGCGGCGGAAGCCGTGGTAGAGCCCGGAGTAATCGGGACTTATCGGACCAGGGAAGCAATTCCGCAGTCTGCAGTCCCCGCCCAGTTAGTCGCAGGAGAACAGGTCGAAGTCGTCTGTCGCGCAGGTTCGCCGTGCCCCTCGCAAACCTTTTGCT>Schizopora_paradoxa DB=Schpa1_AssemblyScaffolds.fasta ACC=scaffold_00261 REGION=31285-31549 TAX=Agaricomycotina; Agaricomycetes; Corticiales; Corticiaceae; HyphodontiaCGGCTGTAATGGCCCTACTTCGGGAATGTGAGGTGGTTACATCAGCGGTATCTTTCTGTGTGCGCTGAGTTCAAGACCGTCCCCTCCAGAAAACTTGGTCGCCAATTCCCATCGGAACGGAAGTCCTGATTGGGTGTGGAATAATCCGGCATTGACTAGTCAGGGAAGCAATTCTGCAGTTAGCATGCCAGGCCGAGTTAGTCTTCGGAGATCAGGCTGGTCCGACAGCTGCGCAAGGAAGCGTGCTCCTCACAGACCTTTCTTT>Scleroderma_citrinum DB=Sclci1_AssemblyScaffolds.fasta ACC=scaffold_29 REGION=361103-361366 TAX=Agaricomycotina; Agaricomycetes; Agaricomycetidae; Boletales; Sclerodermatineae; Sclerodermataceae; SclerodermaTGGTTGTAATGGCCCTAAATTGGGAATGCGAGGCGGATTATGTCAGCGGTCTCATCCACGAGTGCGCTAGTTCAAGACTGTCCCCTCCAGAAAACTTGGTCACCAATCGTCTCTCGCTGCGTAAGCGGTGGTCGGACTTGGAATAATTGGACCTTGACTGGCCAGGGAAGCAATTCCGCATCCAGCAGGTCCTGCCGAGTTAGTCGCCGGAGACCAGGACACGTCGTCAGCCGCGCTGTGGATCGCGCCCTTCGCAGACCTTTT>Sebacina_vermifera DB=Sebve1_AssemblyScaffolds.fasta ACC=scaffold_14 REGION=273590-273872 TAX=Agaricomycotina; Agaricomycetes; Sebacinales; Sebacinaceae; SebacinaAGGCTGTAATGGCCCTAACTCGGGAATACGGGCTGTCACCTTCAGCGGAGTTACTTTACTCGTGCGCTGGGTTCGAAGCTCTCCCCTCCGGAAAATCTGGTTTACGACTTTTCAACTTCCGCGGCGTAAGCCATGGATGGTGGTCGTGTAGCCGACGTTTCAAAATTCTAAATTGGCCAGGGGAGCAATTCCGCAGCCAGGAGCGTCGACCGGATTAGTCTTAGGAGATCAGGAGGTGCTTTCAGTCGCGCGGTGAGGCCGTGCAGCTCGTAAACTTTTGCTC>Lentinus_tigrinus DB=Sisbr1_AssemblyScaffolds.fasta ACC=scaffold_20 REGION=99649-99919 TAX=Agaricomycotina; Agaricomycetes; Polyporales; Lentinaceae; LentinusTGGCTGTAATGGCCCTAAACCGGGAATGTGAGGTGGTCTAAAGCACCAACGGATTCAGTCGTCTGTGCGCTGGTTCAAGACCGTCCCCTCCAGAAAACTTGGTTACTTCGAGTCGCCCGCTCCGTAAGGAGTGGATTGACCTGGTATAATATGCCCTTGACTGGCCAGGGAAGCAATTCCGCAACCTGCTGGGCGTGCCGAGTTAGTCTTAGGAGATCAGGACGAGTCGTCTGTCGCGCAGTTGGCCCGTGCCCCTCACAAACCCCTCTTT>Sistotremastrum_niveocremeum_1 DB=Sisni1_AssemblyScaffolds.fasta ACC=scaffold_1 REGION=209287-209554 TAX=Agaricomycotina; Agaricomycetes; Trechisporales; 'Trechisporaceae'; SistotremastrumGGGCTGTAACGGCCCTCACTCGGGAATGCGAGGTGGTGTAAGCTTCAACGGACCAATAGCCTGGTGCGCTGGTTCAAGACGCTCCCCTCCGGAAAACTTGGTTTCCTCTTAACTCGCGGCGTAAGCCATGAGATCTAATTGGAGTAATGGGTTTGTAACTTGTCAGGGAAGTCATTCCGCAGCAATACAAGTCCGCCGAGTTGATCTTAGGAGATCAGGGGCGTTGTCAGACGCGCTGAGCTACCGTGCCTCTCGCAAACTTTTGACC>Sistotremastrum_niveocremeum_2 DB=Sisni1_AssemblyScaffolds.fasta ACC=scaffold_13 REGION=693054-693318 TAX=Agaricomycotina; Agaricomycetes; Trechisporales; 'Trechisporaceae'; SistotremastrumGGGCTGTAACGGCCCTAACCTAGGAATGCGAGGCGGTGTAAGCATCAACGGACTCATAGCCGAGTGCGCTGGTTCAAGACGCTCCCCTGCGGAAAATTTGGTATTCATTCGACTCGCAGCGTAAGCCGTGAGAAGCGATTGAAGTAATTGGCATGTGAATCGTCAGGGAAGTCATTCCGCAGCGACACTTGCTTGCCGAGTTGATCTTAGCAGATCGGGGGTGTTGTCAGACGCGCTTGGGTACCGTGCCTCTCGCAGACTTTTT>Sistotremastrum_suecicum_1 DB=Sissu1_AssemblyScaffolds.fasta ACC=scaffold_192 REGION=23235-23503 TAX=Agaricomycotina; Agaricomycetes; Trechisporales; 'Trechisporaceae'; SistotremastrumGGGCTGTAACGGCCCTCACTCGGGAATGCGAGGTGGTGTAAGCTTCAACGGACAAATAGCCTGGTGCGCTGGTTCAAGACGCTCCCCTCCGGAAAACTTGGTTTCCTCTTAACTCGCGGCGTAAGCCGTGAGATCTAATTGGAGTAATGGGTTTGTAACTTGTCAGGGAAGTCATTCCGCAGCAATACAAGCCCGCCGAGTTAGTCTTAGGAGATCAGGGGCGTTGTCAGACGCGCCGAGCTACCGTGCCTCTCGCAAACTTTTGCCAT>Sistotremastrum_suecicum_2 DB=Sissu1_AssemblyScaffolds.fasta ACC=scaffold_137 REGION=52466-52730 TAX=Agaricomycotina; Agaricomycetes; Trechisporales; 'Trechisporaceae'; SistotremastrumGGGCTGTAACGGCCTTAGCCTAGGAATGCGAGGCGGTGTAAGCATCAACGGACTCCTACCCGAGTGCGCTGGTTCAAGACGCTCCCCTGCGGAAAATTTGGTATTCATTCCACTCGCAGCGTAAGCCGTGAGAAGCGATTGAAGTAATTGGCATGTGAATCGTCAGGGAAGTCATTCCGCAGCGACACTTGCTTGCCGAGTTGATCTTAGCAGATCGGGGGTGTTGTCAGACGCGCTTGGGTACCGTGCCTCTCGCAGACTTTTT>Sphaerobolus_stellatus_1 DB=Sphst1_AssemblyScaffolds.fasta ACC=scaffold_4 REGION=1626370-1626639 TAX=Agaricomycotina; Agaricomycetes; Phallomycetidae; Geastrales; Sphaerobolaceae; SphaerobolusCGGCTGTAATGGCCAAACCGGGAATGTGATGATGTGTTAGCATCAACGGACAACCGAGTTGTGCGCTGGTTCAAAGCCCTCCCCTCCAGATAATTTGGTTTCGAATCTGTCCTCCGCACGGCAACGGGCGGTCGGACTTCGAATAATGGTCTGTCGACCGGTCAGGGAAGTCATTCCGCAGCCGCAACAGGTCGCCGAGTTAGTCTAAGGAGATCAGGAGGTGTCTTCTAGCCGCGCAACTGGGCCGTGCTCTTCACAAACCCATTCATT>Sphaerobolus_stellatus_2 DB=Sphst1_AssemblyScaffolds.fasta ACC=scaffold_4 REGION=1438024-1438302 TAX=Agaricomycotina; Agaricomycetes; Phallomycetidae; Geastrales; Sphaerobolaceae; SphaerobolusCGGCTGTAACGGCCAAACCGGGAATGCGATGATGTGTCAGCATCAACGGACAACCGAGTTGTGCGCCGGTTCAAAGCCCTCCCCTCCAGATAATTTGGTTTCGAATCTGTCCTCTGCACGGCAACGGGCGGCCGGACTTCGAATAATGGTCTGTTGACCGGTCAGGGAAGTCATTCCGCAGCCGCAACGGGTCGCCGAGTTAGTCTAAGGAGATCAGGAGGTGGCTTCTAGCCGCGCAACTAGGCCGTGCTCTTCGCAAACTCACTCAATCTTTTTGTT>Sphaerobolus_stellatus_3 DB=Sphst1_AssemblyScaffolds.fasta ACC=scaffold_58 REGION=38909-39173 TAX=Agaricomycotina; Agaricomycetes; Phallomycetidae; Geastrales; Sphaerobolaceae; SphaerobolusAGGCTGTAATGGCTTAACTTGGGAATACAGAGTGGTGTAAGCCGCAACGGACAACTCAGTTGTGCGCCGGTTCAAAGCCTGTCTCTCCAGATAGTTTGGTGTAGAGCTGTCCTCGGCATGGCAGCGTGCCGTCGGATATTGGGTGATGGGTCCTTGATTGATCAGGGAAGTCATTCCGCATCCACAACGGCTCGCCGAGTTAGTCTCAGGAGATGAGGCGGTGTCTTCTAGTCGCGCGGTTGGGCGAGTGCACAGTCGATCGTTT>Suillus_brevipes DB=Suibr1_AssemblyScaffolds.fasta ACC=scaffold_69 REGION=168919-169185 TAX=Agaricomycotina; Agaricomycetes; Agaricomycetidae; Boletales; Suillineae; Suillaceae; SuillusCGGCTGTAATGGCCCTAAACCGGGAATGCGAGATAGTTCTTTCCAGCGATCCAACCCATGAGTGCGCTGGTTCAAGACTGTCCCCTCCAGAAAACTTGGTGTCCACATGTCCCTTGCGCCGAAAGGTGTAATCGGACTTGGAATAATCGGTCCTTGATAGGCCAGGGAAGCAATTCCGCAACCTGCAGGATCTGCCGAGTTAGTCGTAGGAGATCAGGGCATGTCATCAGCCGCGCTGTGGGTCGTGCTGCTCGCAAACCTTTTTGC>Suillus_luteus DB=Suilu1_AssemblyScaffolds.fasta ACC=scaffold_350 REGION=2688-2954 TAX=Agaricomycotina; Agaricomycetes; Agaricomycetidae; Boletales; Suillineae; Suillaceae; SuillusCGGCTGTAATGGCCCTAAACCGGGAATGCGAGATAGTTCTTTCCAGCGATCAAACCCATGAGTGCGCTGGTTCAAGACTGTCCCCTCCAGAAAACTTGGTGTCCACATGTCCCTTGCGCCGAAAGGTGTAATCGGACTTGGAATAATCGGTCCTTGATTGGCCAGGGAAGCAATTCCGCAACCTGCAGGATCTGCCGAGTTAGTCGTAGGAGATCAGGGCATGTCATCAGCCGCGCTGTGGGTCGTGCTGCTCGCAAACCTTTTTGT>Trametes_ljubarskyi DB=Tralj1_AssemblyScaffolds.fasta ACC=scaffold_8 REGION=318610-318881 TAX=Agaricomycotina; Agaricomycetes; Polyporales; TrametesTGGCTGTAACGGCCCTAAACCGGGAATGTGAGGAGGTCTAAAGTTCCAACGGGCTCAGTCGTTTGTGCGCTGGTTCAAGACCGTCCCCTCCAGAAAACTTGGTTACCTCGCACCGCCCGCTCCGTAAGGAGTGGGAAGGGCTGGTATAATACGACCTTGACTGGCCAGGGAAGCAATTCCGCAACCTGCAGGACGTGCCGAGTTAGTCTTAGGAGATCAGGACGAGTCGTCTGTTCGTGCAAATGACCCGTGCCCCTCACAAACCCTCCTTT>Trichaptum_abietinum DB=Triab1_1_AssemblyScaffolds.fasta ACC=scaffold_63 REGION=35200-35466 TAX=Agaricomycotina; Agaricomycetes; Polyporales; Polyporaceae; TrichaptumTGGCTGTAATGGTCCTAATTCGGGAATGCGAGGCGGTAACTTCCAGCGAGCTGCCTTTACGTGCGCTGGGTTCAAGACTGTCCCCTCCAGAAAACTTGGACACTATCATTTATCGTGACGGAAGTTGTGAATAAATATAGAATAATATGCTGATGACAGGCCAGGGAAGCAATTCTGCAACCTGCTCAGCAGTCCGAGTTAGTCTTCGGAGATCAGGACAAGTCGTCAGTTCGCGCGAGAGGTCGTGCTCCTTGCAAACCTTTTATT>Tricholoma_matsutake DB=Trima3_AssemblyScaffolds.fasta ACC=scaffold_148 REGION=295615-295880 TAX=Agaricomycotina; Agaricomycetes; Agaricomycetidae; Agaricales; Tricholomataceae; TricholomaCGGCTGTAGTGGCCGCCAACCGGGAATGCGAGAGGTTGATTTCACTGACTAAACCCACAAGTGCGCTGGTTCAAGTCTGTCCCCTCCAGAAAACTTGGTTTCCACAGTTCCTCGCCCTGCAAAGAGTGACCAGTACTGGGAATAATCTGTCCTTGTCGGACCAGGGAAGCAATTCCGCAATCTGCAGGACTCGCCGAGTTAGTCTTTAGAGACAAGGGCATGTCGTCAGTCGCGCAGTGGGTCGGGCATCTCGCAAACCTTTTACT>Trichosporon_oleaginosus_1 DB=Triol1_AssemblyScaffolds.fasta ACC=scaffold_44 REGION=106878-107153 TAX=Agaricomycotina; Tremellomycetes; Tremellales; mitosporic Tremellales; TrichosporonGCACTGTAATGGTTTCGGGAAGATGTTCGTTTTCATAACGGAACTCCTCGTCTCAGTGCGCTCCTTGTACAGCCTTGTCTCCTCTAGCTCGAGTGTTCGAGCCCCACCGAGTCCTCATGGATTTGTGTTGGTACCGCCTCGGCAATCATGCCAGAATACTTCCGGGTACAGATAGGCCGGAAACGGATCAGTCTGGCTGGTGTGGCATTCGTGCTCCAAGAGGTCAGGACAGCTGTAAGCGCGCAGGGCGAGCCGGGCGGGCATCAACCTTTTGTT>Trichosporon_oleaginosus_2 DB=Triol1_AssemblyScaffolds.fasta ACC=scaffold_61 REGION=82859-83130 TAX=Agaricomycotina; Tremellomycetes; Tremellales; mitosporic Tremellales; TrichosporonTCACTGTAATGGTTATAGGGAAGCAGTTTGTAAACTCTAGGGAAATCCTCGGTTCAGCGCGCTCTCGCTACAGCCAGTCTCCTCTAGCTTGGGTGTTCGAGCCCCACCAAATCCTCATGGATTTGCGTTGGTTCAGTCTCGGTGAACATGCCAGTCGCCTCAGGGTGCAGATAGGCCGGCAACGGATCAGTCTGGCTGGTGTGGCATCCAAGCTCCAAGAGAATAGGACAGCTGTAAGCGCGCAGGCCGGGCCCGGCAGGCTGCAACTTTTT>Tulasnella_calospora DB=Tulca1_AssemblyScaffolds.fasta ACC=scaffold_19 REGION=166523-166793 TAX=Agaricomycotina; Agaricomycetes; Cantharellales; Tulasnellaceae; TulasnellaGGGCTGTAGCGGCCCTCACTCGGGAATGCGGAGATGAACTTAGCGATCCCCGTTCTGTGCGCTGGTTCGAATCCTCTCTCTGTCGACATTTGGGTAGTCCATTCTCCGTCCGCGGCGGAAGCCTGTGGATAGGTTTGACGTGATGGGCACCGGTTACCTACCGGGCAAGGGAAGCAATTCCGCAGCCTCCGGTGTCCGCCTAAATAGTCTCAACAGATAGGAGAGTGATTTCAGTCGTGCAGTCGGCCGCCGTCTCCGTAGACCTTTTTTT>Malassezia_globosa DB=Malassezia_globosa.unmasked.fasta ACC=mgl_sf_1_1 REGION=1380364-1380658 TAX=Ustilaginomycotina; Exobasidiomycetes; Malasseziales; Malasseziaceae; Malassezia CACCTGTAATGGGTGCAAGGTTCGGATGAATCTTCCTGCGGCGCGCCCCACTAAATGTTAGTCGTGCCTACGGATGATAAAGGTTTGCTGGCGCGTGCGCGTGCTTCATAGCATGCCCACCTCAGCAGTAAATGGGGGTGTTTAAGGGGCCGCCTCTCTACGGTCTGAAGGATCAGTCAGTACTTCTGAGCAATCCACTCCACCGACCTCTATCCCACGTATTCGTAGGGTCTTCGACAAGCAGGGAGCACATGGCTTGAAAGCGCGCCCAGGTCTAATGAGGGTTCAAACGTTT
