# Supplementary material for: Noncanoncial signal recognition particle RNAs in a major eukaryotic phylum revealed by purification of SRP from the human pathogen Cryptococcus neoformans
Source: Nucleic Acids Res. 2015 Oct 10;43(18):9017–27. doi: 10.1093/nar/gkv819 (PMC4605306; doi:10.1093/nar/gkv819)
Supplement: SUPPLEMENTARY DATA [file supp_gkv819_nar-01320-r-2015-File008.rtf]

Supplementary Dataset S2.  Aligned basidiomycete Srp54 protein sequences assessed in this study>Malassezia_sympodialis_ATCC_42132_64/1-578 gi|465794206|emb|CCU99036.1| unnamed protein productMVLADLGRRLNQAFADLQR-------QPTVDGA-----TVDLLLKQVCSALLASDVNVRLVQKLRQDVKAEVQTMLNEK----SHA-DSQTDAQRKHQVQKIVFEYLVKLVDAG-GSANH-------------------RLEKGRQSVIMFVGLQGSGKTTSCTKLALWYQKR------GYKTGLVCADTFRAGAFDQLKQNAAKAHLPFYGSYTETDPVAIASAGVASFKKNRFDIIIVDTSGRHKQEKDLFDEMIDISAAIKPTQTIMVLDASIGQAAEAQCRAFKEASNYGALFVTKLDGHAKGGGAISAVASTQTPILFIGTGEHIHDLEPFRARPFVSKLLGLGDLSGLMDKIEEVQMNTDVQERHKDMMKKIQEGGAYTIRDWREQITQIMSM----------------------------GPMSKMAGMIPGMGAMFNDG---ANDQMASNKFKTMLYIIDSMTPAELDSDGSCFTTPVARKEAQ----------------------------------------DDKAKPQLRLNARARRVARGSGTSMRDVEEFLLQYRTISTMVKKVGGKNSWLQQMQGG------------RGRG---APPMLPPG------MSREQVMKMQNALPADIKAQLRQ-----PGGREQLMR--QLQSGNVPP------------SLAA-----------MGGM-----------------------PGLGGLPGL-------------GGGLPDMSQMQQMMQGMGG-------LGGLMSAMRGGSS----------------------->Malassezia_globosa_CBS_7966_65/1-617 gi|164658964|ref|XP_001730607.1| hypothetical protein MGL_2403 [Malassezia globosa CBS 7966] >gi|159104503|gb|EDP43393.1| hypotMVLADLGRRLNQAFSDLQR-------QPTLDAA-----SVDQLLKDVCSALLASDVNVRLVQKLRQDVKDEVSSLLNDK----GKA-ESYTDAQRKHQVQRIVFDYLVKLVDPGDGSTKH-------------------RLDKGQQHVIMFVGLQGSGKTTSCTKLALWYQKR------GYRTGLVCADTFRAGAFDQLKQNAAKARIPFYGSYTETDPVAIASAGVTSFKKNRFDVIIVDTSGRHKQEKDLFAEMVDISRAVKPSQTIMVLDASIGQAAEAQCRAFKEAADFGAMFVTKLDGHAKGGGAISAVASTQTPIIFIGTGEHVHDLEPFRARPFISKLLGLGDLTGLMDKMEEVHMNEDVQQRQRDMMKKIQEGGTFTIRDWREQITHIQSM----------------------------GPMSKMAGMIPGMSQMMGHA---PSDATANDKFKIMLYITDSMTPEELDSDGSCFTKPIKTSTSRAAAARSHSDEKA--------------QSRTAANTNANANDKTKSKAPLRLTSRALRVARGSGTSVRDVEEFLMQYRTISQMVKKFGGKNSWLQQMKSGGGS------GAGAGRG---GMPMLPPG------MTREQAMKMQNALPADIKAQLRQ-----PGGREQLMR--QLQSGNVPP------------SLAA-----------MGGL-----------------------PGMPSFPGMG--------LGGAAGGMPDLAQMQSMMQNMGG-------LGGMMQKMMGGGMS---------------------->Tilletiaria_anomala_UBC_951_45/1-622 gi|639560347|gb|KDN38143.1| signal recognition particle proteinMVLSDLGRRINNAFSELNR-------VPTIDDK-----AVDALLKTVCAALLESDVNVRLVSSLRDRVRGSVKEQLNAK----DKNAEQMSEMQRKNVVQKAVYDHLVLLVDPGV---------------------EAFKPTKGKPNVIMFVGLQGSGKTTSCTKLALYYQKR------GFKTGLVCADTFRAGAFDQLKQNASKARIPFYGSYTETDPVAIAGAGVKSFKASKFEIIIVDTSGRHKQESELFNEMVMISDAVKPDQTIMVLDASIGQAAEAQSKAFKEAAGYGAILVTKLDGHAKGGGAISAVAATKTPIIFIGTGEHITDLEPFRAQPFISKMLGMGDMAGLMDKVQEVTMQN--PERQKEMMKKIEEGGTFSIRDWREQIGNIMSM----------------------------GPLSKLAGMIPGMGQMMGQ----GGDDEAGNKMKRMLYITDSMTREELDSDGSLFTAPIESPGASNAQVAE--KISK--------------ELTAQQQQNDKPGRKKKKQVRVRMTARALRVAKGSGTSVREVEEFLVQYKMVAGMVKKMGGKAGWLKQMQGRGGA------GGR----TSGAPPPLPPG------VSREQIMQMQNSLPPEIKAQLRQ-----PGGREKLIQ--QIQSGTMPP------------GLAD-----------LAGG----FG-----------------GGIGGFPGLGALGNIFGGG---AGGMPDMTQMQQMMQQMGG-----GGMANMMRNMMGGGGTAPL------------------->Pseudozyma_flocculosa_PF-1_61/1-657 gi|630966370|ref|XP_007879593.1| hypothetical protein PFL1_03879 [Pseudozyma flocculosa PF-1] >gi|521584854|gb|EPQ28575.1| hypoMVLSDLGRRINTAFQDLSR-------QPTVDAG-----TVDQLLKTVCSALIEADVNVKLVANLRTQVRSEVSALLNDK------ANANWSDAQRRQRVQKAVFDHLVQLVDPNHGQQKTDPESGAQVASATSGPSDVFKPRKGKPNVIMFVGLQGSGKTTSCTKLALYYQKR------GYKTGLVCADTFRAGAFDQLKQNASKNNIPFYGSYTETDPVAISSAGVASFKANRFEVIIVDTSGRHKQEQELFDEMREIDAAVTPDLTIMVLDANIGQAAEAQSRAFKDAAGYGAIIVTKLDGHAKGGGAISAVAATKTPIMFIGTGEHATDLEPFRAQPFISKLLGMGDITGLMDKVEEMQMNGG-QERQREMLKKIEEGGTFSIRDWREQLANIMGM----------------------------GPLSKIAGMIPGMGQLLGGG-GGGDDEAAGSKMKRMMYIIDAMTREELDSDGSLFTAPVRHKPTPREAGAVV-QHQE--------------ASTSGDGSKKKSKKKKKAPTKVRMTARARRVARGSGTSVREVEEFLMQYRMVARMVKQMGGKNSWMRQMSGAAAG------GGGKGKGAMPPAGMLPPG------MTREQVMQMQNSLPPEIKAQLRQ-----PGGREKLMR--DIQSGNIPP------------QLAA-----------AMGG----AG-GG-------------MGGLGGLGGLGGLGSLLGGGGAGGGGMPDMAQMQQMMQSMGG----MGGLANMMKGMMGGGGGGGR------------------->Melanopsichium_pennsylvanicum_4_51/1-637 gi|673527228|emb|CDI53563.1| probable SRP54-signal recognition particle subunitMVLSDLGRRINSAFQDLSK-------VPTVNEA-----SIDQLLKSVCNALIEADVNVKLVSNLRTKVKSQVTALLNDK------ANASWNDAQRRQRVQRAVFDHLVALVDPLHGQTKTDDESGAAVAAGNAGPGDIFKPKKGKPNVIMFVGLQGSGKTTSCTKLALYYQKR------GFKTGLVCADTFRAGAFDQLKQNASKINVPFYGSYTETDPVTISAAGVASFKQNRFEVIIVDTSGRHKQEQELFDEMREIDAAVTPDLTIMVLDANIGQAAEAQSRAFKEAAGYGAIIVTKLDGHAKGGGAISAVAATSTPIMFIGTGEHATDLEPFRAQPFISKLLGMGDMSGLMDKMEEMQMNGG-QERQREMLKKIEQGGIFSIRDWREQLGNIMGM----------------------------GPLSKIAGMIPGMGQMLSGA--GGDDEAAGGKMKRMMFITDAMTAEELDSDGRIFTTPIRSKKPPP-SESKQVTVAE--------------S----STAAGKSKKKKKPQPKVRMNARARRVARGSGTSVKEVEEFLMQYNMVANMVKKMGGKQGWLKQMQGAGA----------GGKGKMPSPGMLPPG------MSREQVMQMQNALPPEIKAQLRQ-----PGGRERLMQ--QIQSGNIPP------------ELAG-----------AMGG-----------------------GGMPGLGGLGGLASMMGGGGPGGGGMPNMAQMQQVSTKHRHWSFRLCQMVTYCTA----------------------------->Pseudozyma_brasiliensis_GHG001_56/1-639 gi|557995958|gb|EST05491.1| hypothetical protein PSEUBRA_SCAF5g02351MVLSDLGRRINSAFQDLSK-------VPTVDAA-----SIDQLLKSVCNALIEADVNVKLVANLRSQVKAQVTALLNDK------ANANWTDAQRRQRVQKAVFDHLVALVDPQHGQTKTEDDSGAVVAAGNAGPGDIFKPKKGKPNVIMFVGLQGSGKTTSCTKLALYYQKR------GFKTGLVCADTFRAGAFDQLKQNASKINVPFYGSYTETDPVAISAAGVASFKQNRFEVIIVDTSGRHKQEQELFDEMREIDAAVTPDLTIMVLDANIGQAAEAQSRAFKEAAGYGAIIVTKLDGHAKGGGAISAVAATSTPIMFIGTGEHAADLEPFRAQPFISKLLGMGDISGLMDKMEEMQMNGG-QERQQEMLKKIGEGGVFSIRDWREQLSNIMGM----------------------------GPLSKIAGMIPGMGQMLSGA--GGDDEAAGSKMKRMMFITDAMTAEELDSDGRMFTAPIRSKKPPA--ESKELAIAE--------------S----STAAGKSKKKKKPQPKMRMTARARRVARGSGTSVREVEEFLMQYRMVGGMVKRFGGKDGFLRQMGMGGP----------GAKGKMPAPGQLPPG------MSREQVMQMQNALPPEIKAQLRQ-----PGGREKLLQ--QIQSGNMPE------------GLAG-----------MGGM-----------------------GGMGG---LGGLANMMGGGGG-G--MPNMAAMQNMMSQMG--GMG--GMMNMMKGMMGGGGAGGAGGA---------------->Ustilago_hordei_59/1-646 gi|388853536|emb|CCF52935.1| probable SRP54-signal recognition particle subunitMVLSDLGRRINSAFQDLSK-------VPTVDAA-----SIDQLLKSVCNALIEADVNVKLVANLRSRVKAQVTALLNDK------ANANWTDTQRRQRVQKAVFDHLVALVDPFHGQTKTDDESGAVVPSSNAGPGDIFKPKKGKPNVIMFVGLQGSGKTTSCTKLALYYQKR------GFKTGLVCADTFRAGAFDQLKQNASKINVPFYGSYTETDPVAISAAGVASFKQNRFEVIIVDTSGRHKQEQELFDEMREIDVAVTPDLTIMVLDANIGQAAEAQSRAFKEAAGYGAIIVTKLDGHAKGGGAISAVAATSTPIMFIGTGEHAADLEPFRAQPFISKLLGMGDISGLMDKMEEMQMNGG-QERQQEMLKKIGEGGVFSIRDWREQLSNIMGM----------------------------GPLSKIAGMIPGMGQMLSGA--GGDDEAAGGKMKRMMFITDAMTAEELDSDGRLFYAPIRSKKPPAPVESKQVAIAE--------------S----STAAGRAKKKKKPLQKVRMTARARRVARGSGTSVKEVEEFLMQYRMVGSMVKKMGGKDGFLRQMGMGGA----------GGKGKMPAPGMLPPG------MSREQVMQMQNALPPDIKAQLRQ-----PGGREKLLQ--QIQSGNIPE------------GLAG-----------MGGM-----------------------GGMGGLGGLGGLANMFGGGAG-GPGGGGMPSMQQIMQGMG--GMG--GMMNMMKEMMSGGGGGGGAPP---------------->Pseudozyma_antarctica_55/1-645 gi|674220060|dbj|GAK64380.1| signal recognition particle proteinMVLSDLGRRINSAFQDLSK-------VPTVDAA-----SIDQLLKSVCNALIEADVNVKLVANLRSQVKSQVTALLNDK------ANANWSDAQRRQRVQKAVFDHLVALVDPLHGQSTTDDESGAVVPSANAGPGDIFKPKKGKPNVIMFVGLQGSGKTTSCTKLALYYQKR------GFKTGLVCADTFRAGAFDQLKQNASKINVPFYGSYTETDPVAISAAGAASFKQNRFEVIIVDTSGRHKQEQELFDEMREIDAAVTPDLTIMVLDANIGQAAEAQSRAFKDAAGYGAIIVTKLDGHAKGGGAISAVAATKTPIMFIGTGEHAADLEPFRAQPFISKLLGMGDITGLMDKMEEMQMNGG-QERQREMLKKIEEGGIFSIRDWREQLSNIMGM----------------------------GPLSKIAGMIPGMGQMLSGA--GGDDEAAGGKMKRMMFITDAMTAEELDSDGRLFYAPIRSKKPPVSADTKEVAIAE--------------S----STAAAKSKKKKKPQQKVRMTARARRVARGSGTSVKEVEEFLMQYRLVSSMVKKMGGKQGWLKQMQGLGG----------GGKGKMPAPGQLPPG------MSREQVMQMQNALPPEIKAQLRQ-----PGGREKLLQ--QIQSGNIPE------------GLAG-----------LGGM----GGLGG-------------MGGMGGMPGLGGLANMMGG-----GGMPDMAQMQKMMQSM---GGG--GMMNMMKGLMGGATPPPQH------------------>Pseudozyma_aphidis_DSM_70725_53/1-645 gi|573031513|gb|ETS64732.1| hypothetical protein PaG_00684MVLSDLGRRINSAFQDLSK-------VPTVDAA-----SIDQLLKSVCNALIEADVNVKLVANLRSQVKSQVTALLNDK------ANANWSDAQRRQRVQKAVFDHLVALVDPLHGQSTTDDESGAVVPSANAGPGDIFKPKKGKPNVIMFVGLQGSGKTTSCTKLALYYQKR------GFKTGLVCADTFRAGAFDQLKQNASKINVPFYGSYTETDPVAISAAGVASFKQNRFEVIIVDTSGRHKQEQELFDEMREIDAAVTPDLTIMVLDANIGQAAEAQSRAFKDAAGYGAIIVTKLDGHAKGGGAISAVAATKTPIMFIGTGEHAADLEPFRAQPFISKLLGMGDITGLMDKMEEMQMNGG-QERQREMLKKIEEGGIFSIRDWREQLSNIMGM----------------------------GPLSKIAGMIPGMGQMLSGA--GGDDEAAGGKMKRMMFITDAMTAEELDSDGRLFYAPIRSKKPPAPADTKEVAIAE--------------S----STAAAKSKKKKKPQQKVRMTARARRVARGSGTSVKEVEEFLMQYRLVSSMVKKMGGKQGWLKQMQGLGG----------GGKGKMPAPGQLPPG------MSREQVMQMQNALPPEIKAQLRQ-----PGGREKLLQ--QIQSGNIPE------------GLAG-----------LGGM----GGLGG-------------MGGMGGMPGLGGLANMMGG-----GGMPDMAQMQKMMQSM---GGG--GMMNMMKGLMGGATPPPQR------------------>Pseudozyma_antarctica_T-34_54/1-645 gi|443894400|dbj|GAC71748.1| signal recognition particle, subunit Srp54MVLSDLGRRINSAFQDLSK-------VPTVDAA-----SIDQLLKSVCNALIEADVNVKLVANLRSQVKSQVTALLNDK------ANANWSDAQRRQRVQKAVFDHLVALVDPLHGQSTTDDESGAVVPSANAGPGDIFKPKKGKPNVIMFVGLQGSGKTTSCTKLALYYQKR------GFKTGLVCADTFRAGAFDQLKQNASKINVPFYGSYTETDPVAISAAGVASFKQNRFEVIIVDTSGRHKQEQELFDEMREIDAAVTPDLTIMVLDANIGQAAEAQSRAFKDAAGYGAIIVTKLDGHAKGGGAISAVAATKTPIMFIGTGEHAADLEPFRAQPFISKLLGMGDITGLMDKMEEMQMNGG-QERQREMLKKIEEGGIFSIRDWREQLSNIMGM----------------------------GPLSKIAGMIPGMGQMLSGA--GGDDEAAGGKMKRMMFITDAMTAEELDSDGRLFYAPIRSKKPPVSADTKEVAIAE--------------S----STAAAKSKKKKKPQQKVRMTARARRVARGSGTSVKEVEEFLMQYRLVSSMVKKMGGKQGWLKQMQGLGG----------GGKGKMPAPGQLPPG------MSREQVMQMQNALPPEIKAQLRQ-----PGGREKLLQ--QIQSGNIPE------------GLAG-----------LGGM----GGLGG-------------MGGMGGMPGLGGLANMMGG-----GGMPDMAQMQKMMQSM---GGG--GMMNMMKGLMGGATPPPQR------------------>Ustilago_maydis_521_60/1-638 gi|71015804|ref|XP_758844.1| hypothetical protein UM02697.1 [Ustilago maydis 521] >gi|46098350|gb|EAK83583.1| hypothetical protMVLSDLGRRINSAFQDLSK-------VPTVDAA-----SIDQLLKSVCNALIEADVNVKLVANLRSQVKSQVTALLNDK------TNANWSDAQRRQRVQKAVFDHLVALVDPLHGQTKTDDDSGAVVASANAGPGDIFKPKKGKPNVIMFVGLQGSGKTTSCTKLALYYQKR------GFKTGLVCADTFRAGAFDQLKQNASKINVPFYGSYTETDPVAISAAGVASFKQNRFEVIIVDTSGRHKQEQELFDEMREIDTAVTPDLTIMVLDANIGQAAEAQSRAFKQAAGYGAIIVTKLDGHAKGGGAISAVAATKTPIMFIGTGEHAADLEPFRAQPFISKLLGMGDISGLMDKMEEMQMNGG-QERQQEMLKKIGQGGIFSIRDWREQLSNIMGM----------------------------GPLSKIAGMIPGMGQMLSGA--GGDDEAAGSKMKRMMFITDAMTAEELDSDGRMFYAPIRSKKPPA--DTKQVAVAE--------------S----STAAAKSKKKKKPQSKVRMTARARRVARGSGTSVKEVEEFLMQYRMVANMCKRVG-KAGLFKQMQG-GA----------GGKGKMPTPGMLPPG------MSREQVMQMQNALPPEIKAQLRQ-----PGGREKLLQ--QIQSGNMPE------------GLAA-----------LGGG---------------------------GLGGLGALANMMGGTG--AGGMPDMSQLASMMHGMGGAGMG--GMMNMMKNMMQGATPSPPPR----------------->Sporisorium_reilianum_SRZ2_57/1-637 gi|343429513|emb|CBQ73086.1| probable SRP54-signal recognition particle subunitMVLSDLSRRINSAFQDLSK-------VPTVDAA-----SIDQLLKSVCNALIEADVNVKLVANLRSQVKSQVTALLNDK------ANANWSDAQRRQRVQKAVFDHLVALVDPLHGQTKTDAESGAVVAAGNAGPGDIFKPKKGKPNVIMFVGLQGSGKTTSCTKLALYYQKR------GFKTGLVCADTFRAGAFDQLKQNASKVNVPFYGSYTETDPVAISAAGVASFKQNRFEVIIVDTSGRHKQEQELFDEMREIDAAVTPDLTIMVLDANIGQAAEAQSRAFKDAAGYGAIIVTKLDGHAKGGGAISAVAATSTPIMFIGTGEHAADLEPFRAQPFISKLLGMGDMSGLMDKMEEMQMNGG-QERQREMLKKIEEGGHFSIRDWREQLSNIMGM----------------------------GPLSKIAGMIPGMGQMLSGA--GGDDEAAGNKMKRMMFITDAMTAEELDSDGRIFTTPVRSKKPPA-ADTKELAVAE--------------S----STAAAKSKKKKKPQPKVRMNARARRVARGSGTSVKEVEEFLMQYRLVGGMVNRMG-KAGWMKQMQGMGA----------GGKGKMPTPGMLPPG------MSREQVMQMQNALPPEIKAQLRQ-----PGGREKLLQ--QIQSGSIPE------------GLAG-----------LGGG---------------------------GMGGLGGLANMMGGGA---GGMPNMAQMQNMMQQMG--GMG--GMMNMMKNMMGGGAGGAPPR----------------->Pseudozyma_hubeiensis_SY62_62/66-699 gi|501304198|dbj|GAC93502.1| hypothetical protein PHSY_001067MVLSDLGRRINTAFQDLSK-------VPTVDAA-----SIDQLLKSVCNALIEADVNVKLVANLRSQVKSQVTALLNDK------ANANWSDAQRRQRVQKAVFDHLVALVDPLHGQTKTDDESGAVVASGNAGPGDIFKPKKGKPNVIMFVGLQGSGKTTSCTKLALYYQKR------GFKTGLVCADTFRAGAFDQLKQNASKINVPFYGSYTETDPVAISAAGVASFKQNRFEVIIVDTSGRHKQEQELFDEMREIDAAVTPDLTIMVLDANIGQAAEAQSRAFKEAAGYGAIIVTKLDGHAKGGGAISAVAATKTPIMFIGTGEHAADLEPFRAQPFISKLLGMGDITGLMDKMEEMQMNGG-QERQQEMLKKIGEGGVFSIRDWREQLSNIMGM----------------------------GPLSKIAGMIPGMGQMLSGA--GGDDEAAGGKMKRMMFITDAMTAEELDSDGRLFYVPIRSKKPPV-ADTKEVAIAE--------------S----STAAEKSKKKKKPQPKVRMTARARRVARGSGTSVKEVEEFLMQYRMVGKMINRFGGKDGWLKQMQG-GA----------GGKGKMPTPGMLPPG------MSREQVMQMQNALPPEIKAQLRQ-----PGGREKLMQ--QIQSGNMPE------------GLAG-----------LAGG---------------------------GMGGLGGLANMFGGAG---GGAGGMPDMSQLMSQMG--GMG--GMMNMMKGMMGGGA---PPR----------------->Puccinia_graminis_f._sp._tritici_CRL_75-36-700-3_52/1-635 gi|331223009|ref|XP_003324178.1| signal recognition particle subunit SRP54 [Puccinia graminis f. sp. tritici CRL 75-36-700-3] >MVLADLGKKINAAFSELQR-------TPLIDDK-----ALDLLLKGISAALLSSDVNVTLVANLRNRVKSKLSPQLEKLIQSP---------AKQKQLVHKTVFDELVALVSPTGSASDHASSSS------RTTEYTPWQPKKGKPNVIMFVGLQGSGKTTSCTKLAVYYRRK------GFKTALVCADTFRAGAFDQLKQNATKAKIPFYGSHTETDPIAISTAGVTRFKRERFEVIIVDTSGRHRQETELFEEMKQISAGVTPNLTIMVLDGAIGQAAEAQTRAFKEAADFGAIIVTKMDGHAKGGGAISAVAAAQTPIMFIGTGEHLHDLERFAPEPFISKLLGMGDIGEFLETMQDLQSATP-SQNREEMRQRIER-GVFTIRDLRDQMSNLTQM----------------------------GSISKIASMIPGMSNMMAGL--GGDGDEMSQKMKRMVFIFDAMSPQELDSDGSIFRKKRRTGD---------------------------------------NTRQLADGEPREPHPRVLRIARGSGTSVDEVEGMLAQHAMFATMVKGAGGKRKWEQQQALKQAQQQSKAMMG---GSRGGKVKQQQVTIEQLVKMAPAQRSQVLRQAPASVRKQIED-----AGGVDAFYALSQAAQRTGGQPSA-------AARARR-------AAAAMGGAGG-GAGGGGL---G---SL------LGGLGGLGGLAG---------GQMPDPEVMRKMMEQMGMGDL-----G----SMFGAGGPGPGNY----------------->Melampsora_larici-populina_98AG31_63/1-617 gi|599368303|ref|XP_007406455.1| hypothetical protein MELLADRAFT_115576 [Melampsora larici-populina 98AG31] >gi|328861050|gb|EGMVLADLGKKINAAFADLQR-------TPLIDEK-----AVDSLLKGLATALLGSDVNVGLVSNLRNRVKSKVTPQLEKLISSP---------AKQKQLVQKTVYDELVALVNPTAASSDATSSS---------SLALPWQPKKGKPNVIMFVGLQGSGKTTSCTKLAVYYQRK------GFRTALVCADTFRAGAFDQLKQNAARAKVPFFGSHTETDPIRISAAGVTKFKRERFEVIIVDTSGRHRQETELFEEMKQISSAVQPNLTIMVLDGAIGQAAEAQTRAFKEAAGFGAIIVTKMDGHAKGGGAISAVAATQTPIMFIGTGEHIHDLERFSPGPFISKVLGMADVDGFLEKMQDLQQSTP-AHKREEMRERMER-GEFTLRDLRDQMTNVAQM----------------------------GPLSKIASMIPGMGAMF-----GGNDDDMSSKMKRMLFIFDAMSPKELDSDGQLFRAPKAQKK---------------------------------------DTNGMDEGVPREPHPRILRIARGSGTSVDEVEAMIAQHVMVATMVKTAGGKRKWEQQQALKAAQQQAAKAAGGAGAGRGGRIKQQQVTIEQLVKMNPAQRQQVLRQAPAAVRKQIED-----AGGVDAFHALSQAAQRNGGVTSS-------AASRAR-------RAAAAGAAGG-P---GGL---A---------------GMMPGLGS---------GQMPDPETMKRMMEQMGMGNL-----G----GMFGGRG----------------------->Wallemia_sebi_CBS_633.66_42/1-561 gi|588258205|ref|XP_006958263.1| signal recognition particle protein [Wallemia sebi CBS 633.66] >gi|388581255|gb|EIM21564.1| siMVLAGLGRQLNSALNSFNK-------SQVIDEK-----TLDGLLKEISKALLESDINVKLVGNLRNRVKTRALKEFKDTNAS-------GNQQ--KQIVQKAIFDELVALVDPQEE---------------------PYKPTKGKTNIIMAVGLQGAGKTTSCTKLAVHYQRK------GFKVGLVCADTFRAGAFDQLKQNATKAKIPFYGSYTETDPVVISEAGVQQFKKSKFDVIIVDTSGRHKQEEDLFEEMRMISQAVKPHMTVLVMDGAMGQAAESQASAFKESSDFGAIIVTKMDGHAKGGGAISAVAATKTPIIFIGTGENLHDIDTFRPRPFVQQLLGMGDMQGLLEHVQDVAMSDP-DK-QKELAKKFEQ-GSLSIRDWAEQIKNVTKL----------------------------GPISKIAGMIPGLNADM---IGQGGDDEASKRIKRMLFITDSMTEKELDSNGTIFLRKGKDGK------------------------------------------------FVGMSRRALRVARGSGTSVREVEELLVQYTMMKS---------GILNMMK-----GQA---GG---------MKPGQPGMPS-----RDQIQQMQKMMPPQLLQKLRS------GGGPEILQ--QLQRGQIPK-------GMDPEMMEKMAQQMGMNPNAMGG----MPQLP----------------------GMGAGGAGFN-PASMLGGM-DFSKMAGMF---GGGR----------------------------------------->Wallemia_ichthyophaga_EXF-994_43/1-580 gi|685943325|ref|XP_009266649.1| Signal recognition particle 54 kDa protein-like protein [Wallemia ichthyophaga EXF-994] >gi|50MVLAGLGRQLNTALNSFSK-------ASVVDEK-----TLDALLKEISKALLESDINVKLVGNLRNHVKTRALKEIKEANAS-------TNHQKSKQIVHKAIFDQLVALVDPQED---------------------PYKPTKGKTNIIMAVGLQGAGKTTSCTKLAVHYQKK------GFKVALVCADTFRAGAFDQLKQNATKAKIPFYGSYTETDPVAISVAGVQQFKKSKFDVIIVDTSGRHKQEEDLFEEMVMISQAVKPQMTVLVMDGAMGQAAESQASAFKQSSDFGAIIVTKMDGHAKGGGAISAVAATQTPIIFIGTGENLHDIDTFRPRPFVQQLLGMGDMQGLMEQVQDVAMSDP-DK-QKELAKKFEQ-GSLSIRDWSEQIKNVTKL----------------------------GPISKIAGMIPGLNADM---IGQGGDDEASKRIKRMLFITDSMTVKEMDSDGSIFLRKGTDGK------------------------------------------------YVGMSKRALRVARGSGTSVREVEELLVQYTMMKS---------GILNMMK-----GQAGGAGG---------MKPGQPGMPS-----RDQVQQMQKMMPPQLLQKLKS------GGGPEILQ--QLQRGQIPK-------GMDPEMMEKMAQQMGMNPNAMGGGGGGMPQLPGMPQMPQMPQMP----------GMG-GAGGFN-PASMLGGM-DFSKMAGMF---GGK------------------------------------------>Microbotryum_violaceum_p1A1_Lamole_50/1-607 gi|634348753|gb|KDE04130.1| hypothetical protein MVLG_05421MVLADLGRKLNQAISSLSG-------SSPIDET-----ALDLTLKTVCTALLESDVNVQLVKRLRDKVKAIVLPPLVELQTRDGNQTDAVAGQKGKQVIHKTVFDELVALVDPGDD------------------APPAFNPKKGRTSVIMMVGLQGAGKTTTCTKLATHYTRR------GFKTALVCADTFRAGAFDQLKQNAVKAKVPFFGSYTETDPVSIALSGVTKFRSERFEVIIVDTSGRHRQESELFEEMVQISEAVEPDMTVLVLDGAIGQAAEPQSRAFKDASNFGAIIVTKLDGHAKGGGAISAVAATKTPIIFIGTGEHLHDLEKFSPRPFISKMLGMGDLTGLVEHAQEMANANP-QR-RENLMKKLEK-GEFTIGDLKEQMATITGM----------------------------GPLSKLTSMIPGMGDMM----GGNAD-EAAKRMRRIAFIFDSMTQEELASDGSLFRPLKKGEKKKESTKTKPKSSNRATTSRSVKGKDKENASTVAKEEKKVEEKDLSEMEPREPNPRVLRIARGSGTTVDEVEELLAQHQMFSRMVKQAGGKTGWASRMRQQQ--MMAARRGG-AGAEMRGMPPMGPDGMPDLSKLTPAQQERMTQMMGQM-----------NPG---------------------------MMSQMASMMGG-------GGMPG--IDGAGGMPDMSKLMS-------------------MFG-VG---GGAR--------------------------------------------------------->Rhodotorula_glutinis_ATCC_204091_49/1-612 gi|342321672|gb|EGU13604.1| putative Signal recognition particle 54 kDa proteinMVLQDLGKRLNTAINSLTQ-------QSAIDEQ-----ALDAVLKSVCAALLESDVNVLLVKRLRDKVKGKVLPQLEEIQAKQGQDADAMQGNKAKQIIQKAIYDELVALVDPGDA------------------APPAFNPVKGKTQVIMMVGLQGAGKTTTCTKLATHYQRR------GLKVALVCADTFRAGAFDQLKQNATKAKVPFFGSYTETDPVSISLQGVTKFRSERFDVIIVDTSGRHRQESELFDEMRQIQDAVEPNMVVLVLDGAIGQAAEAQSRAFKEASNFGAIIVTKLDGHAKGGGAISAVAATNTPIIFIGTGEHLHDLEKFSPQPFISKMLGMGDMQGLVERAQEMALANP-ER-QEKMMKKLEK-GEFTVRDLKDQLATVMGMCVAGSFLRSSRSTAFELLTGKCRAYAYRGPLSNLASMIPGMSEMM----GGGGEEDVSRRMKRVAFIFDSMTTEELDSDGSLFRDSVPSAKGKEKEK----------------------D---------EEKDPSNPMGLREPNKRVLRVARGSGTSVQEVEELLAQHQMFAKMAKRMGGKNGLMGKLAARG--GAGGRPGGMPGLGGAGMPPLGPGGMPDLSKLSPSQMRAMQSMLPPGAREMMS-----RPG---------------------------AMEEMQKMMSG-------AG----------GLGGLGNLMG-------------------G---MG---GGMGD---MLKGM---MGGR----------------------------------------->Rhodosporidium_toruloides_46/1-586 gi|647398372|emb|CDR42226.1| RHTO0S06e11188g1_1MVLQDLGKRLNTAINSLTQ-------QSAIDEQ-----ALDAVLKSVCAALLESDVNVLLVKRLRDKVKGKVLPQLEEIQAKQGQDADAMQGNKAKQIIQKAIYDELVALVDPGDA------------------APPAFNPVKGKTQVIMMVGLQGAGKTTTCTKLATHYQRR------GLKVALVCADTFRAGAFDQLKQNATKAKVPFFGSYTETDPVSISLQGVTKFRSERFDVIIVDTSGRHRQESELFDEMRQIQDAVEPDMVVLVLDGAIGQAAEAQSRAFKEASNFGAIIVTKLDGHAKGGGAISAVAATNTPIIFIGTGEHLHDLEKFSPQPFISKMLGMGDMQGLVERAQEMALANP-ER-QEKMMKKLEK-GEFTVRDLKDQLATVMGM----------------------------GPLSNLASMIPGMSEMM----GGGGEEDVSRRMKRVAFIFDSMTTEELDSDGSLFRDSVPSAKGKEKEK----------------------E-------ETTEKDPSNPMGLKEPNKRVLRVARGSGTSVQEVEELLAQHQMFAKMAKRMGGKNGLMGKLAARG--GAGGRPGGMPGLGGAGMPPLGPGGMPDLSKLSPSQMRAMQSMLPPGAREMMS-----RPG---------------------------AMEEMQKMMSG-------AG----------GLGGLGNLMG-------------------G---MG---GGMGD---ILKGM---MGGR----------------------------------------->Rhodosporidium_toruloides_NP11_48/1-579 gi|472581665|gb|EMS19388.1| signal recognition particle receptor subunit alphaMVLQDLGKRLNTAINSLTQ-------QSAIDEQ-----ALDAVLKSVCAALLESDVNVLLVKRLRDKVKGKVLPQLEEIQAKQGQDADAMQGNKAKQIIQKAIYDELVALVDPGDA------------------APPAFNPVKGKTQVIMMVGLQGAGKTTTCTKLATHYQRR------GLKVALVCADTFRAGAFDQLKQNATKAKVPFFGSYTETDPVSISLQGVTKFRSERFDVIIVDTSGRHRQESELFDEMRQIQDAVEPDMVVLVLDGAIGQAAEAQSRAFKEASNFGAIIVTKLDGHAKGGGAISAVAATNTPIIFIGTGEHLHDLEKFSPQPFISKMLGMGDMQGLVERAQEMALANP-ER-QEKMMKKLEK-GEFTVRDLKDQ-----------------------------------GPLSNLASMIPGMSEMM----GGGGEEDVSRRMKRVAFIFDSMTTEELDSDGSLFRDSVPSAKGKEKEK----------------------E-------ETTEKDPSNPMGLKEPNKRVLRVARGSGTSVQEVEELLAQHQMFAKMAKRMGGKNGLMGKLAARG--GAGGRPGGMPGLGGAGMPPLGPGGMPDLSKLSPSQMRAMQSMLPPGAREMMS-----RPG---------------------------AMEEMQKMMSG-------AG----------GLGGLGNLMG-------------------G---MG---GGMGD---ILKGM---MGGR----------------------------------------->Mixia_osmundae_IAM_14324_40/1-523 gi|358060767|dbj|GAA93538.1| hypothetical protein E5Q_00182MVLADLGKRINGALSQLSA-------ASVIDEQ-----VLDSILKEIATALFEADVNVKLVANLRNKVKTKVAPALGSS-------------GNKKSVIQRAVFDELVQLVDPGPE------------------AAAPFTPKKGQSNVIMFVGLQGAGKTTSCTKLAIHYKRR------GYKTCLVCADTFRAGAFDQLKQNATKAKIPFYGSYTETDPVVISAQGVHKFREERFDIIIVDTSGRHRQETELFEEMMQISRQVKPDQTIMVLDGAIGQAAESQSRAFKEAADFGAIIVTKMDGHAKGGGAISAVAATKTPIIFIGTGEHLHDLEKFSPEPFISKMLGMGDMQGLMETVQEVHQNNP-NQ-RAEMLKKLEA-GVFTVRDLKDQMSNIMSM----------------------------GPLSKIAQMIPGMGDML----GGENSEAASHTIKRMMYIFDAMTAEELESDGKLFHTSPTNTEYR-------------------------------------------EGQLREPNKRVLRIARGSGTSVQECEGLLAQHRMFAEMAKKAGGKGGWMQKAKQMQ--QGGGA------------LPQG--RMPS-----MD---QLSRMLPAGMAEQVRA-----MGGPEAMMK--QMG---------------GMAGLQNMMKG-------MGGGA---------------------------------------------------------------------------------------------------------->Mixia_osmundae_IAM_14324_41/1-528 gi|658163248|gb|KEI37964.1| hypothetical protein L969DRAFT_51658MVLADLGKRINGALSQLSA-------ASVIDEQARPFPVLDSILKEIATALFEADVNVKLVANLRNKVKTKVAPALGSS-------------GNKKSVIQRAVFDELVQLVDPGPE------------------AAAPFTPKKGQSNVIMFVGLQGAGKTTSCTKLAIHYKRR------GYKTCLVCADTFRAGAFDQLKQNATKAKIPFYGSYTETDPVVISAQGVHKFREERFDIIIVDTSGRHRQETELFEEMMQISRQVKPDQTIMVLDGAIGQAAESQSRAFKEAADFGAIIVTKMDGHAKGGGAISAVAATKTPIIFIGTGEHLHDLEKFSPEPFISKMLGMGDMQGLMETVQEVHQNNP-NQ-RAEMLKKLEA-GVFTVRDLKDQMSNIMSM----------------------------GPLSKIAQMIPGMGDML----GGENSEAASHTIKRMMYIFDAMTAEELESDGKLFHTSPTNTEYR-------------------------------------------EGQLREPNKRVLRIARGSGTSVQECEGLLAQHRMFAEMAKKAGGKGGWMQKAKQMQ--QGGGA------------LPQG--RMPS-----MD---QLSRMLPAGMAEQVRA-----MGGPEAMMK--QMG---------------GMAGLQNMMKG-------MGGGA---------------------------------------------------------------------------------------------------------->Trichosporon_asahii_var._asahii_CBS_2479_8/1-566 gi|401882649|gb|EJT46899.1| signal recognition particle 54 kDa proteinMVLADLGARLHGALNQFSK-------TSSVDET-----AIDALVKELCAALLESDVNVKLVSQLRAKVKAKVKRNLEDAEKAG------GREVNKKNAVQKAVFDELVALVDPGTE---------------------PYKPVKGKVNVIMAVGIQGAGKTTTCTKLAVHYQRR------GFRTCLVCADTFRAGAFDQLKQNATKAKIPFFGSYTETDPVTIAYQGVEKFRKERFDVIIVDTSGRHKQESELFEEMVSISKAVKPDMTVMVLDASIGQAAEGQSRAFKDSSDFGSIIVTKLDGHAKGGGAISAVAATKTPIIFLGTGEHLNDLEKFAPQPFISKLLGMGDIQGLMEHMQDMARANP-DG-QKDLAKKLGE-GKFTIRDWKDQLSNIMNM----------------------------GSLSKIASMVPGMSGMM----EQGGEEEAAAKLKRMIFITDAMRTDELDSDGLIF-----------------------------------------------------------------RVAQGSGTSVREVEELLAQARMMAGMARQAGGQNGWMSAMQKMQ--AAAGG------------KPLGPNGMPS-----QQQIQAMRNAMPPELLKKLRA---AGPGGAQKMMA--EMMGG----------GAPGGMDMGAMMKS-------MMGGGG----MPGMPGMGGGGEAMKA------------MGGMGG-MGGMGGGMPDMSQMMKMM---GGMGM-----G---------GR----------------------->Trichosporon_asahii_var._asahii_CBS_8904_9/1-557 gi|406700686|gb|EKD03851.1| signal recognition particle 54 kDa proteinMVLADLGARLHGALNQFSK-------TSSVDET-----AIDALVKELCAALLESDVNVKLVSQLRAKVKAKVKRNLEDAEKAG------GRE---------AVFDELVALVDPGTE---------------------PYKPVKGKVNVIMAVGIQGAGKTTTCTKLAVHYQRR------GFRTCLVCADTFRAGAFDQLKQNATKAKIPFFGSYTETDPVTIAYQGVEKFRKERFDVIIVDTSGRHKQESELFEEMVSISKAVKPDMTVMVLDASIGQAAEGQSRAFKDSSDFGSIIVTKLDGHAKGGGAISAVAATKTPIIFLGTGEHLNDLEKFAPQPFISKLLGMGDIQGLMEHMQDMARANP-DG-QKDLAKKLGE-GKFTIRDWKDQLSNIMNM----------------------------GSLSKIASMVPGMSGMM----EQGGEEEAAAKLKRMIFITDAMRTDELDSDGLIF-----------------------------------------------------------------RVAQGSGTSVREVEELLAQARMMAGMARQAGGQNGWMSAMQKMQ--AAAGG------------KPLGPNGMPS-----QQQIQAMRNAMPPELLKKLRA---AGPGGAQKMMA--EMMGG----------GAPGGMDMGAMMKS-------MMGGGG----MPGMPGMGGGGEAMKA------------MGGMGG-MGGMGGGMPDMSQMMKMM---GGMGM-----G---------GR----------------------->Tremella_mesenterica_DSM_1558_7/1-584 gi|589282370|ref|XP_007006915.1| hypothetical protein TREMEDRAFT_72197 [Tremella mesenterica DSM 1558] >gi|392574127|gb|EIW6726MVLADLGARLHGALSQLSR-------APVVDDQ-----VLDALLKELTAALLESDVNVKLVASLRNKVKTKVKKSLEESAAAG------GREVNRKNVVQKAVFDELVALVDPGVE---------------------PYKPIKGKANVLMAVGIQGAGKTTTCTKLAVHYQRR------GFRTCLVCADTFRAGAFDQLKQNATKAKIPFYGSYTETDPVAIASLGVEKFRKERFEVIIVDTSGRHKQESELFEEMVAISNAVKPDMTIMVLDASIGQAAEGQSRAFKDSADFGAIIVTKLDGHAKGGGAISAVAATKTPIIFLGTGEHMNDLERFAPQPFVSKLLGMGDMQGLMEHMQDMARANP-ER-QKEMAKKLEQ-GKFSLRDWREQLSSIMNM----------------------------GSLSKIASMIPGLPAGV----MDGSEEEATMKMKRLMFITDAMRQDELDSDGLIFFSFDKAGH------------------------------------------------PVGLNRRARRVARGSGTSLREVEELLAQARMMAGMARQAGGQNGWMSAMQKMQ--AAAGG------------KPLGPNGQPS-----PAQIEAMRRAMPPEMMRKLRS---MGPHGAQKMMQ--EMMGGSGAGPGAGPGAGPGAMDFGSMMRG-------MMGG---GGGGGGMPDMGQMQQAMQA------------MGG-------GGGGMPDMSQLMRMM---GGGR----------------------------------------->Cryptococcus_neoformans_var._grubii_H99_2/1-587 gi|405120876|gb|AFR95646.1| signal recognition particle protein SRP54MVLADLGTRLHGAWNQLSK-------ASVIDDK-----VIDGVLKELCAALLESDVNVKLVASLRTKVKAKVKKSLEESEKTG------GREANKKNVVQKAVFDELVALVDPGTE---------------------PYKPVKGKTNVLMAVGIQGAGKTTTCTKLAVHYQRR------GFRTCLVCADTFRAGAFDQLKQNATKAKIPFYGSYTETDPVAIASLGVEKFRKERFDVIVVDTSGRHKQESELFEEMVAIGAAVKPDMTLMVLDASIGQAAEGQSRAFKDSADFGAIIVTKLDGHAKGGGAISAVAATKTPIIFLGTGEHLNDLERFAPQPFISKLLGMGDMQGLVEHMQDMARANP-DR-QKDLAKKLEQ-GKFSIRDWREQLSNIMNM----------------------------GSISKIASMIPGLPAGI----MDGNEEEASAKLKRLIFITDAMRADELDSDGSIFVSYDKQGN------------------------------------------------PVGLNKRAKRVAKGSGTSLRELEDLLVQARMMAGMAKQAGGQNGWMSAMQKMQ--AAAGG------------KPLGPNGQPS-----PAQIEAMRKAMPPELVRKLRA---AGPQGAQKMMQ--DMMGGMGGMPGMGGAGGPGGMDLGNMMRM-------LGGGG---GGGGGMPDMSQMQEMMKG------------MGMGG----GGMGGMPDMSQLMKMM---GGSG----------------------------------------->Cryptococcus_neoformans_var._grubii_H99_3/1-578 gi|540384047|gb|AGV14434.1| signal recognition particle protein SRP54, variant 1MVLADLGTRLHGAWNQLSK-------ASVIDDK-----VIDGVLKELCAALLESDVNVKLVASLRTKVKAKVKKSLEESEKTG------GREANKKNVVQKAVFDELVALVDPGTE---------------------PYKPVKGKTNVLMAVGIQGAGKTTTCTKLAVHYQRR------GFRTCLVCADTFRAGAFDQLKQNATKAKIPFYGSYTETDPVAIASLGVEKFRKERFDVIVVDTSGRHKQESELFEEMVAIGAAVKPDMTLMVLDASIGQAAEGQSRAFKDSADFGAIIVTKLDGHAKGGGAISAVAATKTPIIFLGTGEHLNDLERFAPQPFISKLLGMGDMQGLVEHMQDMARANP-DR-QKDLAKKLEQ-GKFSIRDWREQLSNIMNM----------------------------GSISKIASMIPGLPAGI----MDGNEEEASAKLKRLIFITDAMRADELDSDGSIFVSYDKQGN------------------------------------------------PVGLNKRAKRVAKGSGTSLRELEDLLVQARMMAGMAKQAGGQNGWMSAMQKMQ--AAAGG------------KPLGPNGQPS-----PAQIEAMRKAMPPELVRKLRA---AGPQGAQKMMQ--DMMGGMGGMPGMGGAGGPGGMDLGNMMRM-------LGGGG---GGGGGMPDMSQMQEMMKG------------MGMGG----GGMGGMPGELP----------------------------------------------------->Cryptococcus_neoformans_var._neoformans_JEC21_4/1-591 gi|58267554|ref|XP_570933.1| Signal recognition particle 54 kDa protein [Cryptococcus neoformans var. neoformans JEC21] >gi|134MVLADLGTRLHGAWNQLSK-------ASVIDDK-----VIDGVLKELCAALLESDVNVKLVASLRTKVKTKVKKSLEESEKAG------GREANKKNVVQKAVFDELVALVDPGTE---------------------PYKPVKGKTNVLMAVGIQGAGKTTTCTKLAVHYQRR------GFRTCLVCADTFRAGAFDQLKQNATKAKIPFYGSYTETDPVAIASLGVEKFRKERFDVIIVDTSGRHKQESELFEEMVAIGAAVKPDMTLMVLDASIGQAAEGQSRAFKDSADFGAIIVTKLDGHAKGGGAISAVAATKTPIIFLGTGEHLNDLERFAPQPFISKLLGMGDMQGLVEHMQDMARANP-DR-QKDLAKKLEQ-GKFTIRDWREQLSNIMNM----------------------------GSISKIASMIPGLPAGI----MDGNEEEASAKLKRLIFITDAMRADELDSDGSIFVSYDKQGN------------------------------------------------PVGLNKRAKRVAKGSGTSLRELEDLLVQARMMAGMAKQAGGQNGWMSAMQKMQ--AAAGG------------KPLGPNGQPS-----PAQIEAMRKAMPPELVRKLRA---AGPQGAQKMMQ--DMMGGMGGMPGMGGAGGPGGMDLGNMMRM-------LGGGGGGGGGGGGMPDMSQMQEMMKN------------MGMGG---GGGMGGMPDMSQLMKMM---GGSG----------------------------------------->Cryptococcus_gattii_WM276_5/1-590 gi|321259271|ref|XP_003194356.1| signal recognition particle 54 kDa protein [Cryptococcus gattii WM276] >gi|317460827|gb|ADV225MVLADLGTRLHGAWNQLSK-------ASVIDDK-----VIDSVLKELCAALLESDVNVKLVASLRTKVKAKVKKSLEESEKAG------GREANKKNVVQKAVFDELVALVDPGTE---------------------PYKPVKGKTNVLMAVGIQGAGKTTTCTKLAVHYQRR------GFRTCLVCADTFRAGAFDQLKQNATKAKIPFYGSYTETDPVAIASLGVEKFRKERFDVIIVDTSGRHKQESELFEEMVAIGAAVKPDMTLMVLDASIGQAAEGQSRAFKDSADFGAIIVTKLDGHAKGGGAISAVAATKTPIIFLGTGEHLNDLERFAPQPFISKLLGMGDMQGLVEHMQDMARANP-DR-QKDLAKKLEQ-GKFTIRDWREQLSNIMNM----------------------------GSISKIASMIPGLPAGI----MDGNEEEASAKLKRLIFITDAMRADELDSDGSIFVSYDKEGN------------------------------------------------PVGLNKRAKRVAKGSGTSLRELEDLLVQARMMAGMAKQAGGQNGWMSAMQKMQ--AAAGG------------KPLGPNGQPS-----PAQIEAMRKAMPPELVRKLRA---AGPQGAQKMMQ--DMMGGMGGMPGMGGAGGPGGMDLGNMMRM-------LGGGGG-GGGGGGMPNMSQMQEMMKG------------MGMGG---GGGMGGMPDMSQLMKMM---GGSG----------------------------------------->Rhizoctonia_solani_AG-1_IA_39/1-597 gi|443926065|gb|ELU44808.1| signal recognition particle proteinMVLADLGRKLNDAFAQLQR-------APVVDDK-----VLDAILKDVCSALLESDVNVKLVASLRQKVKATAKAAFEGG-ADK------GKEANRKGVVQKAVFDELVRLVDPGVE---------------------PYKPVKGKSNVIMAVGLQGNGKTTTCTKARTPSHLTHSKLPHTHSPKNIACDTFRAGAFDQTRQSATKAKVAYFGSYTETDPVAIASQGVAKFKKERFDVIIVDTSGRHRQEAELFQEMVQIGEAVTPDMTVLVLDASIGQAAEAQSRAFKESSNFGAIIVTKMDGHAKGGGAISAVAATQTPIIFLGVGEHLTDLDKFSPQPFISKMLGMGDMQGLMETIQDMAMSNP-DK-HKEMAKKLEE-GKLSIRDWREQISNVMSM----------------------------GPISKLASMIPGLPAEL----LQGGDEEGSRRMKRMLFITDSMTAEELDSDGMIFLERGPDGT------------------------------------------------PTGLSRRVLRVARGSGTNVREVEETLTQYRMMANMAKAAGGKNGWMSAIQKMQ--QAAG-------------PPRGPNGMPT-----QQQIADMRRALPPGMLQNLQKMRQ--GGGIKEMMR--SMMGGANAD-------PQELEEMEAMMAQ-------MAKG------G-----------------GMPGLPGMGGMG-----------GMPDMSAMMRMM---GMGGM-----G---------GASKTQSLLMMSKNAVNDDTSSRRE>Rhizoctonia_solani_AG-3_Rhs1AP_34/1-570 gi|576989516|gb|EUC62235.1| signal recognition particle 54 kDa protein, putativeMVLTDLGRKINDAFAQLQR-------APVVDDK-----VLDAILKDVCAALLESDVNVKLVATLRQKVKATAKAAFEGG-ADK------GKEANRKGVVQKAVFDELVRLVDPGVE---------------------PYKPTKGKSNVIMAVGLQGNGKTTTCTKLAVYYQK------RGFKTCIVCADTFRAGAFDQTRQSATKAKVAYFGSYTETDPVAIASQGVAKFKKERFDVIIVDTSGRHRQETELFQEMVQIGEAVTPDMTVLVLDASIGQAAEAQSRAFKESSNFGAIIVTKMDGHAKGGGAISAVAATQTPIIFLGVGEHLTDLDKFSPQPFISKMLGLGDMQGLMETIQDMAMSNP-DK-HKEMAKKLEE-GKLSIRDWREQINNVMSM----------------------------GPISKLASMIPGLPAEL----LQGGDEEGTRRMKRMIFITDSMTAEELDSDGMVFLERGPDGT------------------------------------------------PTSLSRRVVRVAKGSGTSVREVEETLTQYRMMANMAKSAGGKNGWMSAIQKMQ--QAAG-------------PPRGPNGMPT-----QQQIAEMRRALPPSMLQNLQKMRQ--GGGIKEMMR--SMMGGADAD-------PQELEEMEAMMAQ-------MSKG------G-----------------GMPGMPGMGGLG----------GGMPDMSAMMRMM---GMGGM-----G---------GGR---------------------->Rhizoctonia_solani_123E_35/1-570 gi|660971673|gb|KEP55303.1| signal recognition particle protein SRP54MVLTDLGRKINDAFAQLQR-------APVVDDK-----VLDAILKDVCAALLESDVNVKLVSTLRQKVKATAKAAFEGG-ADK------GKEANRKGVVQKAVFDELVRLVDPGVE---------------------PYKPTKGKSNVIMAVGLQGNGKTTTCTKLAVYYQK------RGFKTCIVCADTFRAGAFDQTRQSATKAKVAYFGSYTETDPVAIASQGVAKFKKERFDVIIVDTSGRHRQETELFQEMVQIGEAVTPDMTVLVLDASIGQAAEAQSRAFKESSNFGAIIVTKMDGHAKGGGAISAVAATQTPIIFLGVGEHLTDLDKFSPQPFISKMLGLGDMQGLMETIQDMAMSNP-DK-HKEMAKKLEE-GKLSIRDWREQINNVMSM----------------------------GPISKLASMIPGLPAEL----LQGGDEEGTRRMKRMIFITDSMTAEELDSDGMIFLERGPDGT------------------------------------------------PTSLSRRVVRVAKGSGTSVREVEETLTQYRMMANMAKSAGGKNGWMSAIQKMQ--QAAG-------------PPRGPNGMPT-----QQQIAEMRRALPPSMLQNLQKMRQ--GGGIKEMMR--SMMGGADAD-------PQELEEMEAMMAQ-------MSKG------G-----------------GMPGMPGMGGLG----------GGMPDMSAMMRMM---GMGGM-----G---------GGR---------------------->Dacryopinax_sp._DJM-731_SS1_36/1-558 gi|402225128|gb|EJU05189.1| signal recognition particle proteinMVLADLGRRINSAISSLNR-------SPTIDDK-----QLDAALKEICTALLESDVNVKLVASLRSRVRTKVKAALEGG-ADK------AKEAQRRNVVQKTVFDELVSLVNPGVE---------------------PYKPKKGQSNIIMAVGLQGNGKTTTCTKLAVYYQKR------GFKSCIVCADTFRAGAFDQTRQSATKAKVAYFGSYTETDPVVIAAQGVEKFRKERFEIIIVDTSGRHKQEKDLFAEMEQISAAVKPDMTILVLDASIGQAAEAQSRAFKEAADFGAIIVTKMDGHAKGGGAISAVAATQTPIIFLGTGEHLSDLERFAPEPFISKMLGMGDIQGLVEQMQEMAQRDP-EK-QKEVMKNLEQ-GKFSIKDWREQMGNVMSM----------------------------GSISKFASMIPGLPD------MAGSEEEISSKFKRMIYMTDSMTAYELESDGLCFVDMEKDGKT-----------------------------------------------PIGVNWRAQRVARGSGTSVAEVEEMLFQYRMMANMAKGAGGKNGWIQAMQKMQ--AAAGG------------KGRGPNGMPS-----PAQIQAMQKALPPGMLQKLRS---GGMGGIQDMMR--QMMPGG----E-----MPDMAEMQRMMQG-------MGMGG-----------------------------GMPG--------MGGGRGMPNMADMMRMM---GGGR----------------------------------------->Schizophyllum_commune_H4-8_17/1-573 gi|302691416|ref|XP_003035387.1| hypothetical protein SCHCODRAFT_74858 [Schizophyllum commune H4-8] >gi|300109083|gb|EFJ00485.1MVLADLGRKLNAALSSLNR-------APVVDEK-----VLDATLKEITAALLESDVNVKLVASLRSKVKTKVKAIFEG--GDK------TKDVNRKNVIQKAVFDELVALVDPGVE---------------------PYKPKKGHTNVIMAVGLQGNGKTTTCTKLAVHYQKR------GFKSAIVCADTFRAGAFDQTRQSATKAKVAYFGSYTETDPVSIAAQGVAKFKKERFEVIIVDTSGRHKQEQELFEEMILIGQAVKPDMTILVLDASIGQAAEAQARAFKDSADFGAIIVTKMDGHAKGGGAISAVAATQTPIIFLGVGEHLHDLDRFAPQPFISKLLGLGDMQGLIEHMHDLTAQNP-DK-QKEMAKKFEQ-GKLSIRDWREQISNVMSM----------------------------GPISKIASMIPGLPAEM----LQGSDEEGSLRMKRMIYITDSMTAAELDSDGSMFMEWGKDGK------------------------------------------------PTGLTWRVTRVAKGSGTSVKEVEELLCQYRMMANMAKQAGGKNGWLQTMQKIQ--SVAGS------------KGLGANGMPT-----PAQIQAIQRSMPPGMLQQMRKQMQ-GGGGMQEMMK--AMMGQGGGD-------GPDMEEMQRMMAS-------MGQGG-----L----------------GGLGGLGGLGGLG----------GGMPDMSAMMKMM---GMGGG-----A---------GGR---------------------->Serpula_lacrymans_var._lacrymans_S7.9_14/1-578 gi|597940625|ref|XP_007324656.1| hypothetical protein SERLADRAFT_454209 [Serpula lacrymans var. lacrymans S7.9] >gi|336377467|gMVLADLGRKLNAAFSSLSR-------ATVVDEK-----VLDATLKEITAALLESDVNVKLVASLRQKVKVKVKAALDGGSSDK------SKEANRKNLVQKAVFDELVHLVDPGVE---------------------PYKPKKGQCNILMAVGLQGNGKTTTCTKLAVYYQKR------GFKSCIVCADTFRAGAFDQTRQSATKAKVAYFGSYTETDPVVIAAQGVAKFKKERFEVIIVDTSGRHKQESELFREMVQIGEAVKPHMTILILDASIGQAAEAQSRAFKDSADFGAIIVTKMDGHAKGGGAISAVAATKTPIIFLGVGEHLHDLDRFSPQPFISKLLGLGDMQGLMEHMQDLATQNP-DK-QKEMAKKLEE-GKLSIRDWREQIQNVMNM----------------------------GPISKIASMIPGLPQEL----LSGSDEEGSLRMKRMIYITDSMTSTELDSDGAVFMDMSKDGK------------------------------------------------PVGLTWRVTRVAKGSGTSVREVEELLCQYRMMANMAKQAGGKNGWLQAMQKMQ--SIAGG------------RGRGANGMPT-----PAQIQAMQRAMPPGALQQMQRQLRSGGGGMQEMLK--AMMQGQGGD-------QLDMEEMQRMMSQ-------MGS-G-----M----------------GGLGGLGGLPGMGG-------GMGGMPNMGEMFKMM---GMGGG-----G---------TGR---------------------->Serpula_lacrymans_var._lacrymans_S7.3_38/1-566 gi|336364891|gb|EGN93244.1| hypothetical protein SERLA73DRAFT_172158MVLADLGRKLNAAFSSLSR-------ATVVDEK-----VLDATLKEITAALLESDVNVKLVASLRQKVKVKVKAALDGGSSDK------SKEANRKNLVQKAVFDELVHLVDPGVE---------------------PYKPKKGQCNILMAVGLQGNGKTTTCTKLAVYYQKR------GFKSCIVCADTFRAGAFDQTRQSATKAKVAYFGSYTETDPVVIAAQGVAKFKKERFEVIIVDTSGRHKQESELFREMVQIGEAVKPHMTILILDASIGQAAEAQSRAFKDSADFGAIIVTKMDGHAKGGGAISAVAATKTPIIFLGVGEHLHDLDRFSPQPFISKLLGLGDMQGLMEHMQDLATQNP-DK-QKEMAKKTRR-GE------------TKHP----------------------------GPISKIASMIPGLPQEL----LSGSDEEGSLRMKRMIYITDSMTSTELDSDGAVFMDMSKDGK------------------------------------------------PVGLTWRVTRVAKGSGTSVREVEELLCQYRMMANMAKQAGGKNGWLQAMQKMQ--SIAGG------------RGRGANGMPT-----PAQIQAMQRAMPPGALQQMQRQLRSGGGGMQEMLK--AMMQGQGGD-------QLDMEEMQRMMSQ-------MGS-G-----M----------------GGLGGLGGLPGMGG-------GMGGMPNMGEMFKMM---GMGGG-----G---------TGR---------------------->Punctularia_strigosozonata_HHB-11173_SS5_10/1-562 gi|599114634|ref|XP_007385594.1| signal recognition particle protein [Punctularia strigosozonata HHB-11173 SS5] >gi|390597902|gMVLADLGRKLNAALSSLSR-------APVVDEK-----VLDATLKDICAALLESDVNVKLVSQLRSKVRAKVKASLES--GDK------VKDQNRRTVLQKAVFDELVNLVDPGVE---------------------PYKPKKGQSNVIMAVGLQGNGKTTTCTKLAVHYQKR------GFKSCIVCADTFRAGAFDQTRQSATKAKVAYFGSYTETDPVAIAAQGVTKFKKERFEVIIVDTSGRHKQESELFQEMVQIGEAVKPNMTILVLDASIGQAAEAQARAFKDSADFGAIIVTKMDGHAKGGGAISAVAATKTPIIFLGVGEHLADLDRFSPQPFISKLLGLGDMQGLMEHMQDLAQANP-DK-QKEIAKKLEE-GKLSIRDWREQISNIMNM----------------------------GPISKIASMIPGIPQEM----LAGGDEEGTNRMKRMIFITDSMTASELDSDGSPFMEFGKDGK------------------------------------------------PVGITWRVTRVARGSGTSVREVEELLCQYRMMANMAKQAGGKNGWLQTMQKMQ--QAAGG------------KGRGPNGMPT-----PAQIQAMQRSMPPGMLQQMQRQMRS--GGMAEMMK--AMMQGQGGD-------QFDMEEMQRMMSQ-------MGG-G-----L----------------G------GLGGLG--------GMPNMGNMAEMMKMM---GMGS----------------------------------------->Stereum_hirsutum_FP-91666_SS1_20/1-570 gi|597906394|ref|XP_007300496.1| signal recognition particle protein [Stereum hirsutum FP-91666 SS1] >gi|389748926|gb|EIM90103.MVLADLGRKLNAALSSLNR-------ASLVDDK-----VLDALLKEVCAALLESDVNVKLVSQLRQKVKAKVKASLESS-GDK------VKEVNRKNVVQKAIFDELVNLVDPGVE---------------------PYKPRKGHPNVIMAVGLQGNGKTTTCTKLAVHYQKR------GFKSCIVCADTFRAGAFDQTRQSATKAKVAYFGSYTETDPVSIAAQGVAKFKKEKFEVIIVDTSGRHKQEGELFEEMVQIGKAVKPDMTVLVMDASIGQAAEAQSSAFKESADFGAIIVTKMDGHAKGGGAISAVAATKTPIIFLGTGEHLTDLDKFSPQPFISKLLGMGDVQGLMEHMQDLATQNP-DR-QKEMAKKLEE-GKLSIRDWREQIQNVMNM----------------------------GPISKIASMIPGLPQDM----LAGTDEEGSMRMKRMIYITDSMTSAELDSDGSLFLEMGKDGK------------------------------------------------PVDLTWRVTRVARGSGTSVREVEELLCQYQMMANMAKQAGGKNGWLSAMQKMQ--SAAGG------------RGRGANGMPT-----QAQIQAMQRAMPPGMLQNMQKQMR-G-GGLQEMMK--TLMQSQGGG-------DMDMEEMQRMMSQ-------MGG-G-----M----------------GGLGGLGGLG-------------GGMPNMADMFKMM---GGMGG-----G---------SGGR--------------------->Heterobasidion_irregulare_TC_32-1_30/1-568 gi|695539190|ref|XP_009542689.1| signal recognition particle, subunit Srp54 [Heterobasidion irregulare TC 32-1] >gi|575070268|gMVLADLGRKLNAALSSFNR-------APVVDEK-----ALDALLKEVCNALLETDVNVKLVAQLRSKVKVKVKASLESS-GEK------VKETNRKNVVQKAVFDELVHLVDPGVE---------------------PYKPKKGHPNVIM----AGNGKTTTCTKLAVHYQKR------GFKSCIVCADTFRAGAFDQTRQSATKAKVAYFGSYTETDPVAIAAQGVAKFKKEKFEVIIVDTSGRHKQEGELFEEMVQIGNAVKPDMTVLILDASIGQAAEAQSRAFKDSADFGAIIVTKMDGHAKGGGAISAVAATRTPIIFLGTGEHLTDLDRFSPQPFISKLLGLGDVQGLMEHMQDLATQNP-DR-QKEMAKKLEE-GKLSIRDWREQIQNVMNL----------------------------GPMSKIASMIPGLPQEM----LQGSDEEGASRMKRMIYITDSMTSAELDSDGTLFMEVGKDGK------------------------------------------------PVGLTWRVTRVARGSGTSVREVEELLCQYRMMANMAKQAGGKNGWMQAMQKMQ--SAAGG------------RGRGAGGMPT-----PAQIQAMQRAMPPGMVQQMQRQMRSG-GGLQEMMK--TLMQSQGGD-------QMDMEEMQRMMSQ-------MGS-G-----M----------------GGLGALGGLG-------------GGMPGMADMFKMM---GMGGG-----G---------GGGGR-------------------->Pleurotus_ostreatus_PC15_23/1-570 gi|646312014|gb|KDQ33155.1| hypothetical protein PLEOSDRAFT_1091347MVLADLGRKLNAALSSLNR-------APVVDEK-----VLDATLKEITAALLESDVNVKLVASLRQKVKVKVKAALEG--GDK------SKETNRKHLIQKAVFDELVNLVDPGVE---------------------PYKPKKGQSNVIMAVGLQGNGKTTTCTKLAVHYQKR------GFRSAIVCADTFRAGAFDQTRQSATKAKVAYFGSYTETDPVAIAAQGVAKFKKERFEVIIVDTSGRHKQESELFEEMVQISEAVQPNMTVMILDASIGQAAEAQSRAFKESADFGAIIVTKMDGHAKGGGAISAVAATKTPIIFLGVGEHLHDLDRFSPQPFISKLLGLGDVQGLMEHMQDLASQNP-DK-QKEMAKKLEE-GKLSIRDWREQISNVMNM----------------------------GPLSKIASMIPGLPQDL----LQGSDEEGSLRLKRMIYMTDSMTASELDSDGSPFMEVGKDGK------------------------------------------------PIGLTWRVARVAKGSGTSVREVEELLCQYRMMANMAKQAGGKHGWLQAVQKMQ--SAAGG------------KGRGANGMPT-----QAQIQAMQRAMPPGMLQQMQRQMRSG-GGMQEMMK--AMMQGQGGD-------QLDMEELQRMMSS-------MG--------------------------GLGGLGGLGGLG--------GLGGMPNMGDMMKMM---GMGGG-----A---------PSR---------------------->Gloeophyllum_trabeum_ATCC_11539_12/1-572 gi|630346973|ref|XP_007862787.1| hypothetical protein GLOTRDRAFT_10579, partial [Gloeophyllum trabeum ATCC 11539] >gi|521728599MVLADLGRKLNAALSSLSR-------APVVDEK-----VLDATLKEICAALLESDVNVKLVASLRAKVKAKVKASLDSS-SDK------VKDVNKKNILQKAVFDELVHLVDPGVE---------------------PYKPVKGKPNVIMAVGLQGNGKTTTCTKLAVYYQKR------GFKSCIVCADTFRAGAFDQTRQSATKAKVAYFGSYTETDPVAIAAQGVAKFKKERFDVIIVDTSGRHKQESELFEEMVQIGEAVKPNMTVLILDASIGQAAEAQSRAFKETANFGAIIVTKMDGHAKGGGAISAVAATKTPIIFLGTGEHLTDLDRFSPQPFISKLLGLGDMQGLMEHMQDLAQQNP-DK-QKEMAKKLEE-GRLSIRDWREQIQNVMNM----------------------------GPISKIASMIPGLPQEM----LQGSDEEGAMRMKRMIYITDSMTASELDSDGTPFLELGKDGK------------------------------------------------PVGLTWRVTRVARGSGTSVREVEELLCQYRMMANMAKQAGGKHGWLQGIQKMQ--QAAGG------------RGRGPNGMPT-----PAQIEQMKRAMPPGMLQQMQKQMR-G-GGMQEMMK--AMMQGQGGD-------QLDMEEMQRMMSQ-------MGG-G-----L----------------GGLGGLGGLGGLG--------GMGGMPNMNDMFKMM---GMGGG-----G---------R------------------------>Laccaria_bicolor_S238N-H82_11/1-543 gi|170088384|ref|XP_001875415.1| predicted protein [Laccaria bicolor S238N-H82] >gi|164650615|gb|EDR14856.1| predicted proteinMVLADLGRRLNAALSSLNK-------APIVDEK-----VLDATLKEITAALLESDVNVKFVASLRQKVKAKVKATIESAAADK------SKETNRKNLIQKAVFDELVALVDPGVE---------------------PYKPKKGQPNVIMAVGLQGNGKTTTCTKLAVHYQKR------GFKSAIVCADTFRAGAFDQTRQSATKAKVAYFGSYTETDPVSIAAQGVAKFKKERFDVIIVDTSGRHKQESELFEEMVQIGEAVKPDMTVLILDASIGQAAEAQSRAFKESANFGAIIVTKMDGHAKGGGAISAVAATKTPIIFLGVGEHLHDLDRFSPQPFISKLLGLGDMQGLMEHMQDLATQNP-DK-QKEMAKKLEE-GKLSIRDWREQISNVMNM----------------------------GPISKIASMIPGLPQDM----LQGSDEEGSLRMKRMIYITDSMTAGELDSDGAPFMEVGKDGK------------------------------------------------PVGLTWRVTRVAKGSGTSVREVEELLCQYRMMATMAKQAGGKNGWLQAMQKMQ--SAAGG------------KGRGAGGMPT-----PAQIQAMQRSMPPGMLQQMQRQMRSG-M-----------------------------------MAS-------M-GNG-----L----------------GGLGGLGGLG------------GGGMPGMADMFKMM---GMGGA-----R---------------------------------->Coprinopsis_cinerea_okayama7#130_31/1-574 gi|169853092|ref|XP_001833227.1| signal recognition particle protein [Coprinopsis cinerea okayama7#130] >gi|116505605|gb|EAU885MVLADLGRKLNSALSSLNR-------APVIDEK-----VLDATLKEITAALLESDVNVKLVASLRQKVKAKVKAALDGQG-EK------KEPSNKKHLIQKAVFDELVALVDPGVE---------------------PYKPVKGRTNVIMAVGLQGNGKTTTCTKLAAYYQKR------GFKSAIVCADTFRAGAFDQTRQSCTKAKIAYFGSYTETNPVSIAQQGVQKFKKERFEVVIVDTSGRHKQERELFGEMIEISEAVKPDMTVLVLDASIGQAAEGQAKAFKESADFGAIIVTKMDGHAKGGGAISAVAATKTPIIFLGVGEHLHDLDRFSPQPFISKLLGLGDVQGLMEHMQDLATQNP-DK-QKEMAKKLEE-GKLSIRDWREQIQNVMNM----------------------------GPISKIASMIPGLPQEL----LQGSDEEGSMRMKRMIYITDSMTASELDSDGSPFLLTGKDGK------------------------------------------------PTGLTWRVTRVAKGSGTSVREVEELLCQYRMMANMAKQAGGKNGWLSAMQKMQ--QAAGG------------KGRGLNGMPT-----PQQIQAMQRAMPPGMLQQMQRQLRSG-GGMQEMMK--AMMQGQGGD-------QFDIEEMQRMMAQ-------MGGGG-----L----------------GGLGGLGGLGGLG--------GLGGMPNMADMFGKM---MGGMG-----G---------R------------------------>Coniophora_puteana_RWD-64-598_SS2_24/1-571 gi|628828823|ref|XP_007764715.1| signal recognition particle protein [Coniophora puteana RWD-64-598 SS2] >gi|392595811|gb|EIW85MVLADLGRKLNAALSSLNR-------APVVDEK-----VLDATLKEITAALLESDVNVKLVASLRSKVKVKVKASLES--ADK------AKDVARKNIIQKAVFDELVHLVDPGVE---------------------PYKPKKGQTNVIMAVGLQGNGKTTTCTKLAVYYQKR------GFKSCIVCADTFRAGAFDQTRQSATKAKVAYFGSYTETDPVAIAAQGVTKFKKERFDVIIVDTSGRHKQESELFQEMVQISEAVNPNMTVLVLDASIGQAAEAQSQAFKESADFGAIIVTKMDGHAKGGGAISAVAATQTPIIFLGVGEHLYDLDKFSPQPFISKLLGLGDMQGLMEHMHDLAMQNP-DK-QKDMAKKLEE-GKLSIRDWREQIQNVMNM----------------------------GPISKIASMIPGLPQEL----LAGSDEEGSLRMKRMIYITDSMTAEELDSDGSMFMEMAKDGK------------------------------------------------PIGLTWRVTRVARGSGTSVREVEELLCQYRMMANMAKQAGGKNGWLQAVQKMQ--QAGGG------------RGRGANGMPT-----PAQIQAMQRAMPPGMMQQMQRQLRSG-NGLQDMMR--TMMQGQGGD-------QFDMEEMQRMMGQ-------MG--G-----L----------------GGLGGLGGLPGMG--------RGGGMPNIGDMMNMM---GMGGG-----G---------R------------------------>Agaricus_bisporus_var._burnettii_JB137-S8_27/1-562 gi|597967923|ref|XP_007326596.1| hypothetical protein AGABI1DRAFT_111220 [Agaricus bisporus var. burnettii JB137-S8] >gi|409082MVLADLGRKLNAALSSLNR-------APVVDEK-----VLDVMLKEITAALLETDVNVKLVASLRNKVKHKVKALIEQNAGDK------AKEMNRKHLTQKAVFDELVSLVDPGVE---------------------PYKPKKGQCNVVMLVGLQGNGKTTTCTKLAVHYQRR------GFKSAIVCADTFRAGAFDQTRQSATRAKVAYFGSYTETDPVSIAAQGVSKFKKERFEVIIVDTSGRHKQESELFQEMVQIGDAVKPNMTVLVLDASIGQAAEAQSRAFKDSADFGAIIVTKMDGHAKGGGAISAVAATQTPIIFLGVGEHLHDLDRFSPQPFISKLLGLGDMQGLMEHMQDLATQNP-DK-QKEMAKKLEE-GKLSIRDWREQIQNVMNM----------------------------GPISKIASMIPGLPQEM----LQGSDEEGSQRMKRMIYITDSMTAGELDSDGSPFMESGKDGK------------------------------------------------PTGLTWRVTRVARGSGTSVREVEELLCQYRMMANMAKQAGGKNGWLQAMQKVQ--QAAGS------------KGRGPNGMPT-----PAQIQAMRRAMPPGMMQQMQRQMRNG-GGMEEMMK--AMMQGQGGD-------QGDVEEMQRMMAQ-------MGG-G-----M----------------GGLGGLGGLGGL-----------------GDMMKMM---GMGR----------------------------------------->Agaricus_bisporus_var._bisporus_H97_28/1-562 gi|568446816|ref|XP_006458073.1| hypothetical protein AGABI2DRAFT_190440 [Agaricus bisporus var. bisporus H97] >gi|426200103|gbMVLADLGRKLNAALSSLNR-------APVVDEK-----VLDVMLKEITAALLETDVNVKLVASLRNKVKHKVKALIEQNAGDK------AKEMNRKHLTQKAVFDELVSLVDPGVE---------------------PYKPKKGQCNVIMLVGLQGNGKTTTCTKLAVHYQRR------GFKSAIVCADTFRAGAFDQTRQSATRAKVAYFGSYTETDPVSIAAQGVSKFKKERFEVIIVDTSGRHKQESELFQEMVQIGDAVKPNMTVLVLDASIGQAAETQSRAFKDSADFGAIIVTKMDGHAKGGGAISAVAATQTPIIFLGVGEHLHDLDRFSPQPFVSKLLGLGDMQGLMEHMQDLATQNP-DK-QKEMAKKLEE-GKLSIRDWREQIQNVMNM----------------------------GPISKIASMIPGLPQEM----LQGSDEEGSQRMKRMIYITDSMTAGELDSDGSPFMESGKDGK------------------------------------------------PTGLTWRVTRVARGSGTSVREVEELLCQYRMMANMAKQAGGKNGWLQAMQKVQ--QAAGS------------KGRGPNGMPT-----PAQIQAMRRAMPPGMMQQMQRQMRNG-GGMEEMMK--AMMQGQGGD-------QGDVEEMQRMMAQ-------MGG-G-----M----------------GGLGGLGGLGGL-----------------GDMMKMM---GMGR----------------------------------------->Galerina_marginata_CBS_339.88_15/1-568 gi|648168012|gb|KDR81665.1| hypothetical protein GALMADRAFT_239776MVLGDLGRKLNAALSALNR-------APVVDEK-----VLDATLKEITAALLESDVNVKLVASLRQKVKAKVKATLEGNA-DK------SKEANKKNMMQKAVFDELVALVDPGVE---------------------PYKPKKGQSNVIMAVGLQGNGKTTTCTKLAVHYQKR------GFKSAIVCADTFRAGAFDQTRQSATKAKVAYFGSYTETDPVSIAAQGVAKFKKERFDVIIVDTSGRHKQESELFEEMVQIGEAVKPNMTVLILDASIGQAAEAQSRAFKDAADFGAIIVTKMDGHAKGGGAISAVAATKTPIIFLGVGEHLHDLDRFSPQPFISKLLGLGDMQGLMEHMQDLATQNP-DK-QKEMAKKLEE-GKLSIRDWREQISNVMNM----------------------------GPISKIASMIPGLPQEL----LQGSDEEGSLRMKRMIYITDSMTASELDSDGSPFMEMGKDGK------------------------------------------------PIGLTWRVTRVARGSGTSVREVEELLCQYRMMANMAKQAGGKNGWLSAMQKMQ--SAAGG------------KGRGAGGMPT-----PAQIQAMQRAMPPGMLQQMQRQLRGG-GGMQEMMN--AMMQGQGGD-------QMDMEEMQRMMSQ-------MGG-G-----L----------------GGLGGLGGLGGLG-------------GGMGDMLKMM---GMGGG-----G---------R------------------------>Moniliophthora_roreri_MCA_2997_18/1-567 gi|630183556|ref|XP_007846154.1| signal recognition particle 54 kda protein [Moniliophthora roreri MCA 2997] >gi|554913321|gb|EMVLADLGRKLNAALSSLNR-------APVIDEK-----VLDATLKEITAALLESDVNVKLVASLRQKVKAKVKSSLEGSSTDK------AKDVNRKNLIQRAVFDELVSLVDPGVE---------------------PYKPKKGQSNVIMAVGLQGNGKTTTCTKLAVHYQKR------GFKSAIVCADTFRAGAFDQTRQSATKAKVAYFGSYTETDPVAIAAQGVAKFKKERFEVIIVDTSGRHKQESELFEEMVQISEAVKPNMTVLILDASIGQAAEAQSRAFKESADFGAIIVTKMDGHAKGGGAISAVAATKTPIIFLGVGEHLHDLDRFSPQPFISKLLGLGDVQGLMEHMQDLATQNP-DK-QKEMAKKLEE-GKLSIRDWREQIQNVMNM----------------------------GPISKIASMIPGLPQEL----LQGSDEEGTLRMKRMIFITDSMTAMELDSDGTPFMEMGKDGK------------------------------------------------PIGLTWRVTRVARGSGTSVREVEELLCQYRMMANMAKQAGGKNGWLSAMQKMQ--AAAGG------------KGRGANGMPT-----PAQIQAMQRALPPGTLQQMQRQMRSG-GGMQEMMR--AMMQGQGGD-------QMDMEEMQRMMAS-------MGN-G-----L----------------GGLGGLGGLGGMP--------------NMSEMFKLM---GMGGN-----R---------------------------------->Piriformospora_indica_DSM_11827_37/1-563 gi|353227294|emb|CCA77807.1| probable signal recognition particle subunit SRP54MVLADLGRKLNSALSNLNK-------SPVVDEK-----VLDELLKEITKALLESDVNVKLVQTLKKQVKAKVKPALES--TDK------SKEGQRKSIIQKAIFDELVQLVDPGVE---------------------PYKPKKGHPNVIMAVGLQGNGKTTTCTKLAVHYQKR------GFKCCIVCADTFRAGAFDQTRQSATKAKVHFYGSYTETDPVAIASEGVAKFKKERFEVIIVDTSGRHRQESELFEEMKQIQAAVKPDMTVLVLDASIGQAAEAQSRAFKESADFGAIIVTKMDGHAKGGGAISAVAATKTPIIFLGVGEHLNDLDRFSPEPFISQLLGMGDLRGLMEHMSDLARQNP-DR-QKEMTKAFEE-GKLSIRDWREQMQNIMSM----------------------------GPLSKITSKIPGMAGLMEGLAGEGTDEEASRRLKRLICITDSMTPAELDSDGMLFLRIGKDNK------------------------------------------------PTGVTKRVLRVAKGSGASVREVEEVLMQHRMMANVAKQMGGKNGMLQALKGMG--GAGRG------------RGAGPGGMPS-----PAQIQAMRRMMPPGMMQQIQQMQRSG-MTPEQMMR--NMLGGAGGDGD-----MPNMEEMQRMMSQ-------MGMGG-----GG--------------PGGIGSMF-------------------------SRMM---GAMGN-----N---------------------------------->Botryobasidium_botryosum_FD-172_SS1_32/1-576 gi|646290484|gb|KDQ11691.1| hypothetical protein BOTBODRAFT_35122MVLADLGKKINSALAQLNK-------APVIDDK-----TLDSLLKGICAALLESDVNVKLVATLRQRVKANVKSAIEAT-GDK------SNESNRRQIVQKAIFDELVKLVDPGVE---------------------PYKPKKGRPNVIMAVGLQGNGKTTTCTKLAVYYQRR------GFKSCIVCADTFRAGAFDQTRQSATKAKVAYFGSYTETDPVVIAAQGVDKFKKERFEVIIVDTSGRHKQESELFEEMVQIGKAVKPDMTVLVLDASIGQAAEAQSRAFKESADFGAIIVTKMDGHAKGGGAISAVAATNTPIIFLGVGEQMHDLDRFAPEPFISQMLGMGDVQGLMEHMQTVAMQNP-DK-QKEMAKKFEE-GKLSIRDWREQISNVMGM----------------------------GSLSKIANMIPGFPAEM----LQGSEEDASKRMKRMIFITDSMSAQELDSDGMLFMTTGSDGK------------------------------------------------PSGLSWRVMRVAKGSGTSVREVEELLCQYRMMANMAKQAGGKNGWLQAMQKMQ--AAGGG------------RGRGANGMPT-----PAQIQAMQRSMPPGMLQQLRQ---SG-GNMQEMMK--TMMGGQVSD-------E-EMKEMQQMMSQ-------MGVGR-----GGGMPNLSSLMSGLGGLGGLGGLGGLGGLGGMGG--AGGAGGRR--------------------------------------------------------->Auricularia_delicata_TFB-10046_SS5_33/1-567 gi|598015723|ref|XP_007338514.1| signal recognition particle protein [Auricularia delicata TFB-10046 SS5] >gi|393245418|gb|EJD5MVLADLGRKLNNALSQLNR-------APVVDEK-----VLDALLKEICAALLESDVNVKLVSQLRSKVKTKAKAAFEAG-ADK------GKETNRRNVVHKAIFDELVQLVDPGVE---------------------PFKPKKGQPNVIMAVGLQGNGKTTTCTKLAAYYQRR------GFKSCIVCADTFRAGAFDQTRQSATKAKVSFYGSYTETDPVAIAAQGVSKFKKERFEVIIVDTSGRHRQESELFQEMVQIGEAVKPDMTVLVLDASIGQAAEAQSRAFKESSDFGAIIVTKMDGHAKGGGAISAVAATKTPIIFLGVGEHLTDLDKFSPQPFISKLLGMGDVQGLMEHMHDVAMQNP-EK-QKEMAKKFEE-GKLSIRDWKEQVQNVMSM----------------------------GPISKIASMIPGLPQEM----LEGSDEDGARRLKRLIFITDSMTATELDSDGSMFMQMGKDGK------------------------------------------------PTGLTWRVTRVAKGSGTSVREVEEVLCQYRMMAHMAKQAGGKKGWLQAAQKMQ--AAAGG------------RGRGLNGMPS-----PAQIQAMQRAMPPGMQQQLRQ--GGF-GGIQEMMK--AMMGGEGGP---------EMEEMQRMMAQ-------MGG-G-----M-------------GG---LPGMG---G------------GGMPNLGDMFRMM---GGAGR-----G---------AGAGRR------------------->Fomitiporia_mediterranea_MF3/22_29/1-578 gi|595778430|ref|XP_007267758.1| signal recognition particle protein [Fomitiporia mediterranea MF3/22] >gi|393216852|gb|EJD0234MVLADLGRKLNAALSSLRA-------APVVDDK-----VIDALLKEVCAALLESDVNVKLVSQLRSKVKAKVKAQFEAG-ADR------GKEQNRKAVVQKAIFDELVQLVDPGVD---------------------SYKPRKGHPNVIMAVGLQGNGKTTTCTKLAVHYQKR------GFKSCIVCADTFRAGAFDQTRQSATKAKVAYFGSYTETDPVTIAAQGVAKFKKEKFEVIIVDTSGRHKQESELFQEMVQIGEAVKPDMTVLVLDASIGQAAEAQSRAFKESADFGAIIVTKMDGHAKGGGAISAVAATKTPIIFLGVGEHLTDLDRFSPQPFISKLLGLGDVQGLMEHMHDLANANP-EK-QKEMAKKLEE-GKLSIRDWREQIQNIMNM----------------------------GPLSKIASMIPGFPQDM----LQGSDEEGTLRMKRMLYITDSMSAQELDSDGSLFLTFGKDGK------------------------------------------------PDGITWRVTRVARGSGTSVREVEELLCQYRMMANMAKQAGGKNGWMQAVQKMQ--AAAGG------------KGRGAGGMPS-----PAQIQAMQRAMPPGMMQQMQRAMRGG-GGMQEMMK--TMMRGQGGD-------QFDMEEMQRMMSQ-------MGG-G-----M-------------GGLGGLGGLG---GLGGGG---TGGTGGMPNMADMFKMM---GMGGG-----N---------R------------------------>Dichomitus_squalens_LYAD-421_SS1_22/1-571 gi|597972187|ref|XP_007361140.1| signal recognition particle protein [Dichomitus squalens LYAD-421 SS1] >gi|395333501|gb|EJF658MVLADLGRKLNAALSSLNR-------APVVDDK-----VLDALLKEVCAALLESDVNVKLVAQLRQKVKTKVKAALESG-ADK------GKEQNRRHVVQKAVFDELVQLVDPGVE---------------------PYKPKKGHPNVIMAVGLQGNGKTTTCTKLAVYYQRR------GFKSCIVCADTFRAGAFDQTRQSATKAKVAYFGSYTETDPVTIAAQGVAKFKKERFDVIIVDTSGRHKQESELFREMVQIGEAVQPDQTVMVLDASIGQAAEAQARAFKESANFGAIIVTKMDGHAKGGGAISAVAATKTPIIFLGVGEHLHDLDKFSPQPFISKLLGLGDMQGLVEHMQDIATQNP-DK-QKEMAKKLEE-GKLSIRDWREQIQNVMNM----------------------------GPLSKIASMIPGLPQDL----LQGSDEEGTLRMKRMIYITDSMTAAELDSDGSMFMEWGKDGK------------------------------------------------PTGLTWRVQRVARGSGTSVREVEDLLCQYRMMANMAKQAGGKNGWLQAIQKMQ--AAAGG------------RGRGANGMPT-----PAQIQAMQRAMPPGMLQQLQRQMRGG-GGMQEMMK--AMMQGQGGD-------QFDMEEMQRMMQQ-------MGG----------------------GLGGLGGLG---GLG----------GGMPNMGDMFKMM---G-MG-----------------GMGRGGR----------------->Trametes_versicolor_FP-101664_SS1_16/1-578 gi|636628307|ref|XP_008045288.1| signal recognition particle protein [Trametes versicolor FP-101664 SS1] >gi|392558546|gb|EIW51MVLADLGRKLNAALSSLNR-------ASVIDDK-----VLDALLKEVCAALLESDVNVKLVAQLRQKVKAKVKAAIEGG-ADK------GKEQNRRNVVQKAIFDELVGLVDPGVE---------------------PYKPKKGQSNVIMAVGLQGNGKTTTCTKLAVYYQKR------GFKSCIVCADTFRAGAFDQTRQSATKAKVAYFGSYTETDPVSIAAQGVAKFKKERFDVIIVDTSGRHKQESELFKEMVQIGEAVKPNMTVMILDASIGQAAEAQAHAFKESANFGAIIVTKMDGHAKGGGAISAVAATKTPIIFLGVGEHLHDLDKFSPQPFISKLLGMGDVQGLVEHMQDMATQNP-DK-QKEMAKKLEE-GKLSIRDWREQIQNVMNM----------------------------GPLSKIASMIPGLPQDM----LQGSDEEGSLRMKRMIYITDSMTAAELDSDGALFMEWAKDGK------------------------------------------------PTGLTWRVTRVARGSGTSVREVEDLLCQYRMMANMAKQAGGKNGWLSAMQKMQ--AAAGG------------RGRGANGMPT-----QAQIQAMQRAMPPGMLQQMQRQMRSG-GGMQEMMK--AMMQGQGGD-------QFDMEEMQRMMGQ-------MGGGG-----L-------------GGLGGLGGLG---GLG----------GGMPNMGDMMKMM---GGMGG-----M---------PGMGRGR------------------>Jaapia_argillacea_MUCL_33604_21/1-567 gi|646396697|gb|KDQ60949.1| hypothetical protein JAAARDRAFT_31942MVLADLGRKLNAALSSLNR-------APVVDEK-----VLDATLKEVCAALLESDVNVKLVSSLRQKVKTKVKASLEAG-SDK------LKDGNKKNILQKAVFDELVHLVDPGVE---------------------PYKPKKGQPNVIMAVGLQGNGKTTTCTKLAVYYQKR------GYKSCIVCADTFRAGAFDQTRQSATKAKVAYFGSYTETDPVTIAAQGVSKFKKERFDVIIVDTSGRHKQESELFEEMVQISEAVKPHMTVLILDASIGQAAEAQSRAFKDSADFGAIIVTKMDGHAKGGGAISAVAATKTPIIFLGTGEHLTDLDRFSPQPFISKLLGMGDMQGLMEHMQDLAQQNP-DK-QKEMAKKLEE-GKLSIRDWREQIQNVMNM----------------------------GPISKIASMIPGLPQDM----LQGSDEDGAQRLKRMIYISDSMTAAELDSDGSPFMEMGKDGK------------------------------------------------PIGLTCRVTRVAKGSGTSVREVEELLCQYRMMSNMAKQAGGKNGWLQAIQKMQ--SAAGG------------RGRGPNGMPT-----PTQIEAMKRAMPPGMLQQMQRQMRG--GGMQEMMK--AMMQGQGGD-------QMDMEEMQRMMSQ-------MG-GG-----L-------------GG------LG---GLG----------SGMPNMSDMFKMM---G-MG-----------------GMGGGGR----------------->Phanerochaete_carnosa_HHB-10118-sp_26/1-564 gi|599373772|ref|XP_007395680.1| hypothetical protein PHACADRAFT_255925 [Phanerochaete carnosa HHB-10118-sp] >gi|409045874|gb|EMVLADLGRKLNAALSSLNR-------APIVDEK-----VLDTLLKDVCAALLETDVNVKLVAQLRQKVKAKVKASLESG-ADK------GKDVNRKSIVQKAIFDELVQLVDPGVE---------------------PYKPKKGQSNVIMAVGLQGNGKTTTCTKLAVYYQKR------GFKSCIVCADTFRAGAFDQTRQSATKAKVAYFGSYTETDPVAIAAQGVAKFKKERFDVIIVDTSGRHKQESELFQEMVQIGEAVKPNMTIMILDASIGQAAEAQARAFKESSNFGAIIVTKMDGHAKGGGAISAVAVTKTPIIFLGVGEHLHDLEKFSPQPFISKLLGLGDVQGLMEHMQDIATQNP-DK-QKEIAKKLEE-GKLSIRDWREQIQNVMSM----------------------------GPISKIASMIPGLPQEI----VQGSDEEGSMRMKRMIYISDSMTAAELDSDGSPFMEFGKDGK------------------------------------------------PIGLTSRVTRVARGSGTSVREVEELLCQYRMMANMAKQAGGKNGWLQAAQKMQ--QAAGG------------RGRGVNGTPT-----PAQIQAMQRAMPPGMLQQLQRTMRGG-GGMQEMMK--AMMQGQGGD-------AADMEEMQRLMSQ-------MG-GG-----L-------------GG------LG---GLG----------GGMPNMGDMFKMM---G-MG-----------------GGR--------------------->Ceriporiopsis_subvermispora_B_25/1-571 gi|449547302|gb|EMD38270.1| hypothetical protein CERSUDRAFT_113440MVLADLGRKLNAALSSLNR-------APVVDEK-----VLDALLKEVCAALLESDVNVKLVASLRQKVKAKVKVALEGG-ADK------GKEVNRKSVIQKAVYDELVQLVDPGVE---------------------PYKPKKGQPNVIMAVGLQGNGKTTTCTKLAVYYQKR------GFKTSIVCADTFRAGAFDQTRQSATKAKVAYFGSYTETDPVAIAAQGVAKFKKERFDVIIVDTSGRHKQESELFQEMVQIGQAVKPNMTVLILDASIGQAAEAQSRAFKESADFGAIIVTKMDGHAKGGGAISAVAVTKTPIIFLGVGEHLHDLDRFSPQPFISKLLGLGDVQGLMEHMHDLATQNP-DK-QKEIAKKLEE-GKLSIRDWREQIQNVMNM----------------------------GPISKIASMIPGLPQEM----MQGSDEESSLRMKRMIYIVDSMTSSELDSDGSPFMEVGKDGK------------------------------------------------PIGLTWRVTRVARGSGTSVREVEELLCQYRMMANMAKQAGGKNGWLQTIQKMQ--SAAGG------------RGRGANGMPT-----PAQIQAMQRAMPPGMLQQMQRQMRSG-GGMQEMMK--AMMQGQGGD-------QFDMEEMQRMMSQ-------MG-GG-----L-------------GGLGGLGGLG---GLG----------GGMPNMGDMFKMM---G-MG-----------------RGGR-------------------->Fomitopsis_pinicola_FP-58527_SS1_19/1-569 gi|527297732|gb|EPS98546.1| hypothetical protein FOMPIDRAFT_1042441MVLADLGKKLNAALSSLNR-------APVVDEK-----VLDALLKEVCAALLESDVNVKLVASLRQKVKAKVKASLEGG-ADK------GKDTNRKTVVQKAIFDELVQLVDPGVE---------------------PYKPKKGQSNVIMAVGLQGNGKTTTCTKLAVYYQKR------GFKSCIVCADTFRAGAFDQTRQSATKAKVAYFGSYTETDPVAIAAQGVSKFKKERFDVIIVDTSGRHKQESELFEEMVQIGEAVKPNMTVLVLDASIGQAAEAQARAFKDSADFGAIIVTKMDGHAKGGGAISAVAVTKTPIIFLGVGEHLHDLDKFAPQPFISKLLGLGDVQGLMEHMQDIATQNP-DK-QKEMAKKLEE-GKLSIRDWREQIHNVMNM----------------------------GPISKIASMIPGLPQDM----LQGSDEEGAQRMKRMIFITDSMTSRELDSDGTPFLELGKDGK------------------------------------------------PVGLTWRVTRVARGSGTSVREVEELLCQYRMMANMAKQAGGKNGWLQAAQKMQ--AAAGG------------RGRGANGTPT-----PAQIQAMQRAMPPGMLQQLQRQMRGG-GGMQEMMK--AMMQGQGGD-------QFDMEEMQRMMSQ-------MG-GG-----L-------------GGLG---GLG---GLG----------GGMPNMGDMFKMM---G-MG-----------------GGAPR-------------------
